# Design and Synthesis of Brefeldin A-Isothiocyanate Derivatives with Selectivity and Their Potential for Cervical Cancer Therapy

**DOI:** 10.3390/molecules28114284

**Published:** 2023-05-23

**Authors:** Mingying Wang, Xiaoyuan Chen, Ying Qu, Qingyinglu Ma, Huaqi Pan, Haonan Li, Huiming Hua, Dahong Li

**Affiliations:** 1 Key Laboratory of Structure-Based Drug Design & Discovery, Ministry of Education, School of Traditional Chinese Materia Medica, Shenyang Pharmaceutical University, Shenyang 110016, China; 18609881870@163.com (M.W.); nicchen852@163.com (X.C.); quying0822@163.com (Y.Q.); mqyl17808001756@163.com (Q.M.); lihaonan073@163.com (H.L.); 2 Institute of Applied Ecology, Chinese Academy of Sciences, Shenyang 110016, China; panhq@iae.ac.cn

**Keywords:** brefeldin A, isothiocyanates, cervical cancer, apoptosis

## Abstract

Brefeldin A has a wide range of anticancer activity against a variety of tumor cells. Its poor pharmacokinetic properties and significant toxicity seriously hinder its further development. In this manuscript, 25 brefeldin A-isothiocyanate derivatives were designed and synthesized. Most derivatives showed good selectivity between HeLa cells and L-02 cells. In particular, **6** exhibited potent antiproliferative activity against HeLa cells (IC_50_ = 1.84 μM) with no obvious cytotoxic activity to L-02 (IC_50_ > 80 μM). Further cellular mechanism tests indicated that **6** induced HeLa cell cycle arrest at G1 phase. Cell nucleus fragmentation and decreased mitochondrial membrane potential suggested **6** could induce apoptosis in HeLa cells through the mitochondrial-dependent pathway.

## 1. Introduction

In 2020, there were approximately 19.3 million new cancer cases and nearly 10 million cancer deaths in 185 countries worldwide [1]. The top ten new cancer cases were breast (11.7%), lung (11.4%), colorectal (10.0%), prostate (7.3%), stomach (5.6%), liver (5.1%), esophagus (3.1%), cervical (3.1%), thyroid (3.0%) and bladder (3.0%) cancers in turn. The top ten cancer deaths were lung (18.0%), colorectal (9.4%), liver (8.3%), stomach (7.7%), breast (6.9%), esophagus (5.5%), pancreas (4.7%), prostate (3.8%), cervical (3.4%) and leukemia (3.1%) cancers in turn. Compared with 2020, the number of new cancer cases worldwide in 2040 is expected to increase by 47% to 28.4 million. At present, small molecule chemotherapy drugs widely used as anticancer agents in clinic still have common side effects that cannot be ignored [2]. For example, doxorubicin causes cardiotoxicity and neurotoxicity [3], cisplatin causes nephrotoxicity [4] and 5-fluorouracil causes intestinal mucositis and severe diarrhea [5]. Both usual adverse events and frequent drug resistance have promoted the development of new anticancer agents.

**Figure 1:**
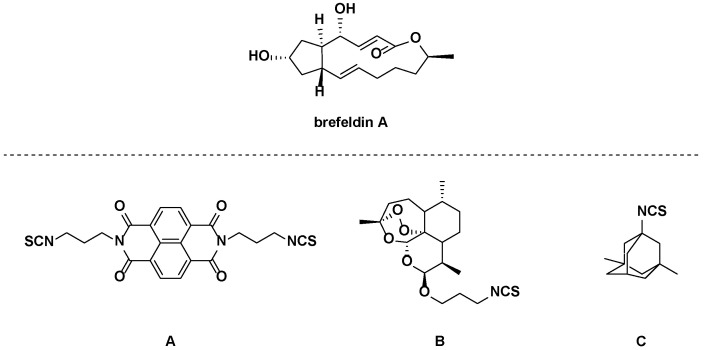
The chemical structures of brefeldin A and representative ITC derivatives.

Natural products play important roles in drug development. In the field of anticancer drugs, 39% of 260 small molecules approved therapeutic agents originated from unaltered natural products or natural product derivatives [6]. Brefeldin A (BFA, Figure 1) is a naturally occurring macrolide antibiotic that revealed significant cytotoxic effects against various mammalian cancer cell lines [7]. BFA and its derivatives are isolated from the fermentation broth of *Penicillium decumbens* [8], *Curvularia lunata* [9], *Penicillium camemberti* [10], *Eupenicillium brefeldianum* [11], *Penicillium janthinellum* [12], etc. BFA promotes endoplasmic reticulum stress by inhibiting protein transport from endoplasmic reticulum to Golgi apparatus, leading to the accumulation of misfolded proteins in endoplasmic reticulum [13]. Further studies have shown that BFA acts to stabilize the ARF-GDP-Sec7 domain protein complex and noncompetitively inhibits the activation of ADP-ribosylation factor 1 (ARF1) by guanine nucleotide exchange factors (GEFs) [14,15]. As a prospective anticancer agent, BFA exhibited its activity through different mechanisms of action, including disrupting cell cycle, cell apoptosis and blocking the mitophagy process [16,17,18]. However, its poor pharmacokinetic properties and remarkable toxicity seriously hindered its clinical application [19,20,21,22]. Hence, BFA is a promising lead compound to be modified and BFA derivatives were designed and synthesized to overcome its defects [23,24,25,26].

Isothiocyanates (ITCs) are produced by enzymatic hydrolysis of glucosinolates, important secondary metabolites in *Brassicaceae* [27,28]. Representative ITCs with anticancer activity include allyl isothiocyanate (AITC), erucin, sulforaphane (SFN), sulforaphene, 6-(methylsulfinyl)hexyl isothiocyanate, benzyl isothiocyanate (BITC) and phenethyl isothiocyanate (PEITC) [29,30]. ITCs exert anticancer effects or cancer prevention by regulating different signaling pathways and protein expression [30,31,32,33,34]. 1,4,5,8-naphthalenetetracarboxylic diimide-ITC derivative **A** (0.5 μM) (Figure 1) induced apoptosis by activating caspase-8, decreased mitochondrial membrane potential and induced cell cycle arrest in G1 phase in human T lymphoblastoid cell line Jurkat [35]. In a series of synthesized artemisinin-ITC derivatives, all derivatives showed more effective cytotoxicity against glioblastoma multiforme U87 cells than the lead compound dihydroartemisinin in vitro [29]. Among them, compound **B** (IC_50_ = 7.41 μM) (Figure 1) with the strongest cytotoxic activity induced cell apoptosis by up-regulating the expression levels of caspase-9, caspase-3, cytochrome c, proapoptotic protein Bax, and down-regulating the expression levels of anti-apoptotic protein Bcl-2. Furthermore, compound **B** led to autophagic cell death and inhibited cell migration.

In addition, ITCs have been shown to be hydrogen sulfide (H_2_S) donors that slowly transport exogenous H_2_S [36]. H_2_S, as the third gasotransmitter discovered after carbon monoxide and nitric oxide, participates in regulating various physiological and pathological processes [37,38,39,40]. Some studies have shown that ITCs reduced adverse reactions and side effects of marketed drugs. ITCs alleviated paclitaxel or oxaliplatin-induced neuropathic pain through their H_2_S-releasing properties and activation of Kv7 channels [41]. SFN was involved in protecting the gastrointestinal mucosa from oxidative damage induced by non-steroidal anti-inflammatory drugs [42]. AITC protected acetaminophen-induced liver injury by activating NRF2 to reduce spontaneous degradation of hepatocytes [43]. Based on the principle of prodrug, compound **C** (Figure 1) was synthesized by replacing the free amino group of memantine with isothiocyanate [44]. Compound **C** released H_2_S through a *L*-cysteine-mediated mechanism, had a protective effect on neuronal inflammation and induced a sharp decline in reactive oxygen species (ROS) production. Moreover, compound **C** reduced A*β*(1-42) self-induced aggregation, protected human neurons and rat microglia from A*β* oligomer-induced damage and promoted autophagy. Besides, erucin and SFN-rivastigmine hybrids demonstrated antioxidant and neuroprotective activities in human neuronal cells [45].

Based on the above-mentioned discoveries, we designed and synthesized 25 novel BFA-ITC derivatives to reduce the toxicity of BFA. Specifically, 4-carboxyphenyl isothiocyanate was chosen from ITCs and it was directly or through different linkers connected to the 4-OH and/or 7-OH of BFA. The antiproliferative activities of BFA, 4-carboxyphenyl isothiocyanate, positive drug paclitaxel and all target derivatives against human liver cancer HepG2, triple-negative breast cancer MDA-MB-231, melanoma A375, lung cancer A549, cervical cancer HeLa and normal liver L-02 cell lines were tested. Significantly, **6** exhibited potent antiproliferative activity against HeLa cells (IC_50_ = 1.84 μM) with no obvious cytotoxic activity to L-02 (IC_50_ > 80 μM), the selectivity index between L-02 and HeLa (SI_HeLa_) was more than 43. Thus, the effects of **6** on cell cycle, apoptosis and mitochondrial membrane potential were further studied in HeLa cell line. As one of the common female malignant tumors, the incidence of cervical cancer is still high, especially in low-middle-income countries. The treatment of cervical cancer is mainly based on surgery and radiotherapy, but the cure rate of advanced patients is very low. According to our in vitro results, **6** shows a good anticancer effect on cervical cancer HeLa cells and potent selectivity, which seems to have the potential for cervical cancer therapy. In summary, **6** is worthy of further study as a potential cervical cancer therapeutic agent.

## 2. Results and Discussion

### 2.1. Chemistry

**Scheme 1:**
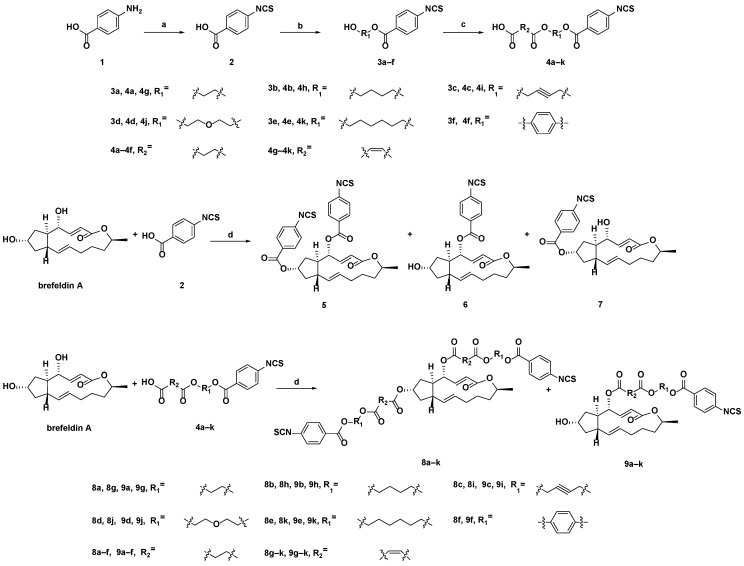
Synthesis of **5**, **6**, **7**, **8a**–**k** and **9a**–**k**. Reagents and conditions: (**a**) 1,1′-thiocarbonyldiimidazole, TEA, anhydrous DCM, 0 °C, 1 h, then hydrochloric acid, *n*-hexane, rt, 2 h; (**b**) corresponding diol, DMAP, EDCI, anhydrous DCM, rt, 12 h; (**c**) succinic anhydride or maleic anhydride, TEA, DMAP, anhydrous DCM, rt, 12 h; (**d**) DMAP, EDCI, anhydrous DCM, rt, 12 h.

The synthesis strategy of the target compounds is shown in Scheme 1. 4-Carboxyphenyl isothiocyanate **2** was synthesized by the reaction of 4-aminobenzoic acid with 1,1′-thiocarbonyldiimidazole in the mixture of anhydrous dichloromethane (DCM) and triethylamine (TEA). Under the catalysis of 4-dimethylaminopyridine (DMAP) and *N*-(3-dimethylaminopropyl)-*N*’’-ethylcarbodiimidehydrochloride (EDCI), 4-carboxyphenyl isothiocyanate reacted with ethylene glycol, 1,4-butanediol, 2-butyne-1,4-diol, diethylene glycol, 1,6-hexanediol and hydroquinone in DCM at room temperature to obtain intermediates **3a**–**f**. **3a**–**f** were reacted with succinic anhydride or maleic anhydride at room temperature in the presence of DMAP and TEA in DCM to obtain intermediates **4a**–**k**. The intermediates **2** and **4a**–**k** were dissolved in DCM, and EDCI, DMAP and BFA were added in turn to obtain the target derivatives **5**, **6**, **7**, **8a**–**k** and **9a**–**k** at room temperature.

### 2.2. Biological Evaluation

#### 2.2.1. Antiproliferative Activity

As shown in Table 1, all derivatives, the lead compound BFA, **2** and positive control paclitaxel were tested for antiproliferative activity in HepG2, MDA-MB-231, A375, A549 and HeLa tumor cells and human liver cells L-02. Except for 4,7-OH disubstituted BFA derivatives **5** and **8a**–**k**, all derivatives showed good antiproliferative activity against five tumor cell lines. For most active compounds, the lowest IC_50_ value occurred on the HeLa cell line, the antiproliferative activity of **9b** and **9j** towards HeLa cells was similar to that shown on A375 and MDA-MBA-231 cell lines. The IC_50_ values of 4-OH monosubstituted derivatives **6** and **9a**–**k** and 7-OH monosubstituted derivative **7** against HeLa cells were 1.84, 2.69, 2.28, 3.63, 0.99, 0.81, 13.12, 3.63, 9.68, 6.59, 1.71, 2.39 and 3.02 μM, respectively. The structure-activity relationship analysis showed that the cytotoxic activity of **9f** with hydroquinone as the linker was the weakest, compared with **9a**–**e**. The cytotoxic activity of derivatives **9a**–**e** linked by succinic anhydride was generally higher than that of derivatives **9g**–**k** linked by maleic anhydride. By comparing the antiproliferative activity of derivatives **9a**–**e** linked with succinic anhydride, it was found that **9d** (R_1_ = diethylene glycol) had the best cytotoxic activity. Among the derivatives **9g**–**k** with maleic anhydride as the linker, **9j** with the same diethylene glycol linker had the best cytotoxic activity. While **6** and **7** were directly linked by the lead compound BFA and **2** through ester bonds, and their activity against cancer cell lines was quite different. Specifically, the antiproliferative activity of **6** against five tumor cell lines was generally stronger than that of **7**, and the toxicity to L-02 cell line was much weaker than that of **7**.

Although the antiproliferative activity of the derivatives against five tumor cell lines was not comparable to that of BFA and paclitaxel, their SI_HeLa_ were generally good, especially for **6**, **8d**, **8h**, **9d**, **9e** and **9k**, whose SI_HeLa_ were all greater than 9, suggesting good selectivity. Among them, **6** showed the best selectivity to HeLa cells, and its IC_50_ value to HeLa cells was 1.84 μM. In L-02 cells, **6** showed no obvious cytotoxic activity and its IC_50_ value to L-02 cells was greater than 80 μM. Thus, **6** was selected for preliminary antiproliferative mechanism study in HeLa cells.

#### 2.2.2. **6** Induced HeLa Cell Cycle Arrest in G1 Phase

**Figure 2:**
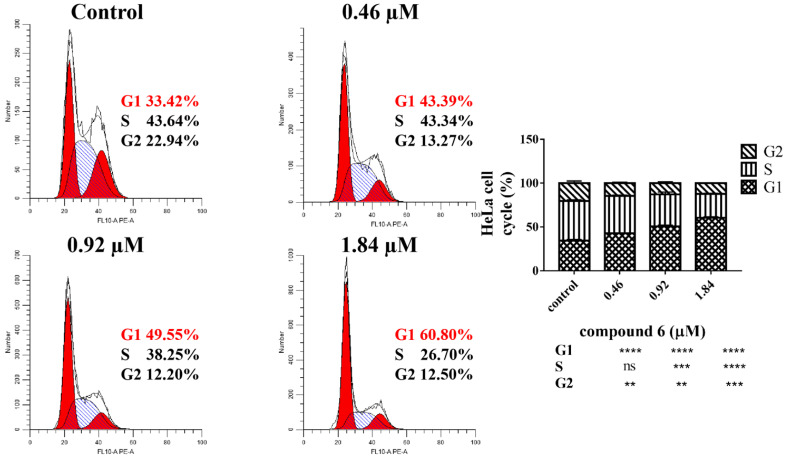
**6** induced HeLa cell cycle arrest in G1 phase. HeLa cells were fixed with 70% ethanol after 48 h administration of **6** (0, 0.46, 0.92 and 1.84 µM). PI staining was performed after RNase A treatment, and red fluorescence at the excitation wavelength of 488 nm was recorded by flow cytometry. Data are represented as mean ± SD of three independent experiments. ** *p* < 0.01, *** *p* < 0.001, **** *p* < 0.0001, ns—not significant.

Propidium iodide (PI) flow cytometric assay was performed to test the DNA distribution at each stage to evaluate the regulatory effect of **6** on HeLa cell cycle. As shown in Figure 2, after HeLa cells were incubated with **6** (0, 0.46, 0.92 and 1.84 µM) for 48 h, cells arrested in G1 phase gradually increased from 33.42% of the control group to 43.39%, 49.55% and 60.80%, respectively. Accordingly, cells in S phase and G2 phase were down-regulated from 43.64% and 22.94% to 26.70% and 12.50%, respectively. **6** induced HeLa cell cycle arrest in G1 phase, indicating that HeLa cells could not pass through the checkpoint at the end of G1 phase for subsequent DNA replication and mitosis, which may lead to apoptosis.

#### 2.2.3. **6** Induced HeLa Cell Nucleus Fragmentation

**Figure 3:**
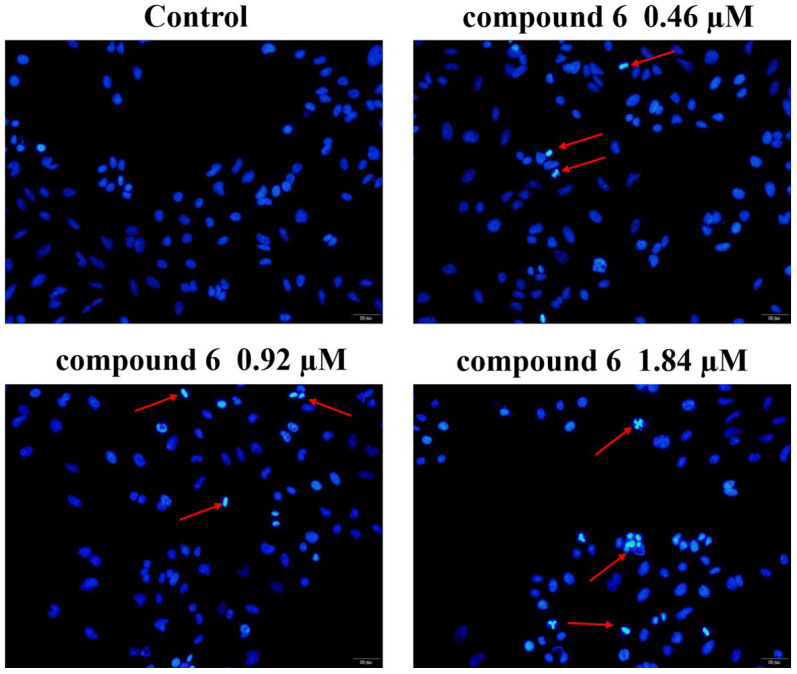
**6** induced chromatin condensation and nuclear fragmentation in HeLa cells. HeLa cells were fixed with paraformaldehyde after 48 h administration of **6** (0, 0.46, 0.92 and 1.84 µM) and then an appropriate amount of Hoechst 33258 staining solution was added and placed at room temperature for 10 min. Observation of staining by fluorescence microscope.

Deviant morphological characteristics such as pyknosis and nuclear fragmentation often occur in the early stage of apoptosis. As shown in Figure 3, Hoechst33258 staining result showed that with the increase of **6** (0, 0.46, 0.92 and 1.84 µM), the bright spots with higher fluorescence intensity gradually appeared in the HeLa cells, indicating the occurrence of nuclear fragmentation. Hoechst33258 staining indicated that **6** induced HeLa cell apoptosis.

#### 2.2.4. **6** Induced Apoptosis in HeLa Cells

**Figure 4:**
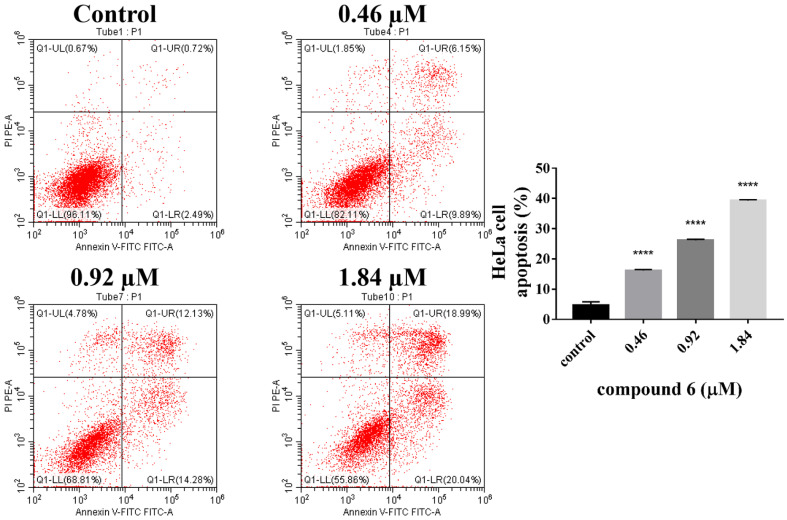
**6** induced apoptosis in HeLa cells. HeLa cells were collected by 0.25% trypsin (without ethylenediaminetetraacetic acid (EDTA)) digestion after 48 h administration of **6** (0, 0.46, 0.92 and 1.84 µM). Annexin V-FITC and PI were added successively in dark for 15 min, and apoptosis was detected by flow cytometry. Data are represented as mean ± SD of three independent experiments. **** *p* < 0.0001.

Annexin V-fluorescein isothiocyanate (FITC) and PI were used to further detect apoptosis. As shown in Figure 4, after HeLa cells were incubated with **6** (0, 0.46, 0.92 and 1.84 µM) for 48 h, the percentage of cells in the early and late stages of apoptosis increased from 3.21% to 16.04%, 26.41% and 39.03%, respectively. Combined with the results of Hoechst33258 staining, it was confirmed that **6** induced apoptosis of HeLa cells in a dose-dependent manner.

#### 2.2.5. **6** Decreased Mitochondrial Membrane Potential in HeLa Cells

**Figure 5:**
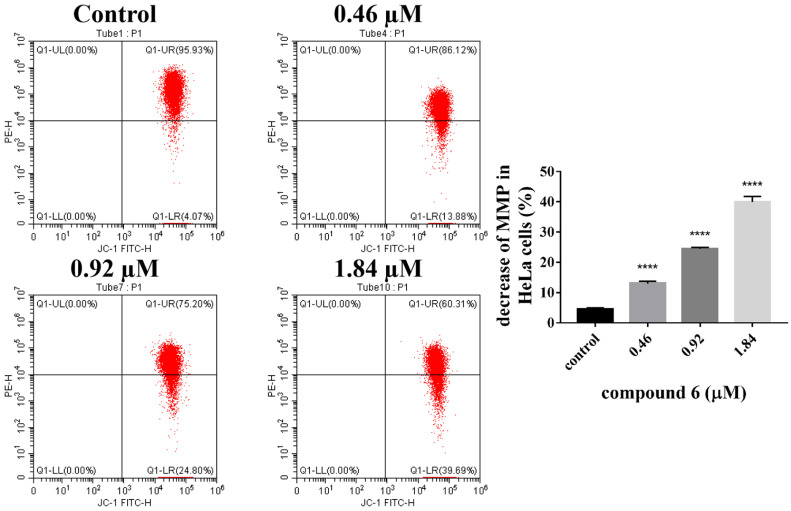
**6** induced the decrease of mitochondrial membrane potential in HeLa cells. Cells were collected by 0.25% trypsin (without EDTA) digestion after 48 h administration of **6** (0, 0.46, 0.92 and 1.84 µM). HeLa cells were suspended in a mixture of 1×Incubation Buffer and JC-1, and incubated for 20 min. The fluorescence changes of JC-1 were detected by flow cytometry. Data are represented as mean ± SD of three independent experiments. **** *p* < 0.0001.

The destruction of mitochondrial membrane potential is a landmark event in the early stage of apoptosis. 5,5′,6,6′-tetrachloro-1,1′,3,3′-tetraethylbenzimi-dazolylcarbocyanine iodide (JC-1) is a membrane permeable cationic dye that selectively enters mitochondria and emits green fluorescence in cells with decreased mitochondrial membrane potential. As shown in Figure 5, with the increase of **6** (0, 0.46, 0.92 and 1.84 µM), the green fluorescent indicating that HeLa cells gradually increased from 4.07% to 13.88%, 24.80% and 39.69%, indicating HeLa cells with decreased mitochondrial membrane potential increased significantly with the increase of **6** concentration.

## 3. Materials and Methods

### 3.1. Chemistry

BFA was provided by Dr. Huaqi Pan from Shenyang Institute of Applied Ecology, Chinese Academy of Sciences, and the specific production process referred to the patent [46]. Anhydrous DCM was prepared based on standard methods, and other chemical materials and reagents were purchased from commercial suppliers. The reaction products were purified by 200–300 mesh silica gel column chromatography. The analysis of thin-layer chromatography was carried out on silica gel GF254 plate. With tetramethylsilane (TMS) as the internal standard, ^1^H nuclear magnetic resonance (NMR) and ^13^C NMR spectra of all derivatives were measured on a Bruker (Billerica, MA, USA) 600 MHz spectrometer (AVANCE III HD 600 MHz), and the chemical shifts data were reported in *δ*. High-resolution electrospray ionization mass spectrometry (HR-ESIMS) spectra were determined on an Waters (Milford, MA, USA) XEVO G2-XS QToF apparatus.

#### 3.1.1. Synthesis of Intermediates **4a**–**k**

4-Aminobenzoic acid (1.2 g, 8.8 mmol) and 1,1′-thiocarbonyldiimidazole (2.1 g, 11.8 mmol) were dissolved in 30 mL anhydrous DCM, and then TEA (1.4 mL, 10.0 mmol) was added to react for 1 h under ice bath. 12 mL of *n*-hexane solution containing 3 mL hydrochloric acid was added and the mixture was keep at room temperature for 2 h. After 2 h, the reaction mixture was poured into 30 mL of ice water. After the solid was completely precipitated, the cake was filtered and washed several times, and the rice white powder 4-carboxyphenyl isothiocyanate **2** (1.5 g) was obtained, crude yield: 95%.

**2** (179.2 mg, 1.0 mmol) and ethylene glycol (112 μL, 2.0 mmol) were dissolved in 20 mL DCM, and EDCI (575.1 mg, 3.0 mmol) and a catalytic amount of DMAP (7.2 mg, 0.06 mmol) were added to react for 12 h at room temperature. After the reaction was completed, 40 mL water was added to the reaction solution, extracted three times with DCM, the combined DCM was washed once with saturated sodium chloride solution, dried with Na_2_SO_4_, filtered, concentrated under reduced pressure, and purified by silica gel column chromatography (petroleum ether (PE): ethyl acetate (EA) = 4:1) to obtain colorless powder intermediate **3a** 130.2 mg, yield: 60%.

**3a** (130.2 mg, 0.6 mmol) and succinic anhydride (100.1 mg, 1.0 mmol) were dissolved in 10 mL DCM, and then TEA (278 μL, 2.0 mmol) and a catalytic amount of DMAP (7.2 mg, 0.06 mmol) were added at room temperature. After 12 h, 20 mL of water was added, extracted three times with DCM, the combined DCM was washed once with saturated sodium chloride solution, dried with Na_2_SO_4_, filtered and concentrated under reduced pressure to obtain intermediate **4a** 160.5 mg, crude yield: 85%.

Referring to the above reaction, ethylene glycol was replaced with 1,4-butanediol, 2-butyne-1,4-diol, diethylene glycol, 1,6-hexanediol or hydroquinone to obtain intermediates **3b**–**f**. The structures of **3a**–**f** were characterized by ^1^H NMR (Figures S1–S6). Then **3a**–**f** were reacted with succinic anhydride or maleic anhydride to obtain intermediates **4a**–**k**. The crude yields of **4b**–**k** were between 64–88%.

##### 2-Hydroxyethyl 4-Isothiocyanatobenzoate (**3a**)

White powder, yield: 60%. ^1^H NMR (CDCl_3_, 600 MHz), *δ*: 8.05 (2H, dt, *J* = 8.6, 1.8 Hz, Ar-H), 7.27 (2H, dt, *J* = 8.7, 1.7 Hz, Ar-H), 4.46 (2H, m, -COOCH_2_-), 3.96 (2H, m, -OCH_2_-).

##### 4-Hydroxybutyl 4-Isothiocyanatobenzoate (**3b**)

White powder, yield: 54%. ^1^H NMR (CDCl_3_, 600 MHz), *δ*: 8.02 (2H, dt, *J* = 8.8, 2.0 Hz, Ar-H), 7.26 (2H, dt, *J* = 8.7, 1.9 Hz, Ar-H), 4.36 (2H, t, *J* = 6.6 Hz, -COOCH_2_-), 3.72 (2H, t, *J* = 6.5 Hz, -OCH_2_-), 1.87 (2H, m, -CH_2_-), 1.72 (2H, m, -CH_2_-).

##### 4-Hydroxybut-2-yn-1-yl 4-Isothiocyanatobenzoate (**3c**)

White powder, yield: 65%. ^1^H NMR (CDCl_3_, 600 MHz), *δ*: 8.05 (2H, dt, *J* = 8.7, 2.0 Hz, Ar-H), 7.27 (2H, dt, *J* = 8.7, 2.0 Hz, Ar-H), 4.96 (2H, t, *J* = 1.8 Hz, -COOCH_2_C≡), 4.33 (2H, t, *J* = 1.8 Hz, -OCH_2_C≡).

##### 2-(2-Hydroxyethoxy)ethyl 4-Isothiocyanatobenzoate (**3d**)

White powder, yield: 67%. ^1^H NMR (CDCl_3_, 600 MHz), *δ*: 8.05 (2H, dt, *J* = 8.6, 1.9 Hz, Ar-H), 7.27 (2H, dt, *J* = 8.7, 1.9 Hz, Ar-H), 4.50 (2H, m, -COOCH_2_-), 3.84 (2H, m, -OCH_2_-), 3.76 (2H, m, -OCH_2_-), 3.65 (2H, m, -OCH_2_-).

##### 6-Hydroxyhexyl 4-Isothiocyanatobenzoate (**3e**)

White powder, yield: 64%. ^1^H NMR (CDCl_3_, 600 MHz), *δ*: 8.02 (2H, dt, *J* = 8.7, 1.9 Hz, Ar-H), 7.26 (2H, dt, *J* = 8.7, 2.0 Hz, Ar-H), 4.31 (2H, t, *J* = 6.7 Hz, -COOCH_2_-), 3.65 (2H, t, *J* = 6.5 Hz, -OCH_2_-), 1.78 (2H, m, -CH_2_-), 1.60 (2H, m, -CH_2_-), 1.46 (4H, m, 2×-CH_2_-).

##### 4-Hydroxyphenyl 4-Isothiocyanatobenzoate (**3f**)

White powder, yield: 36%. ^1^H NMR (DMSO-*d*_6_, 600 MHz), *δ*: 9.51 (1H, s, Ar-OH), 8.14(2H, dt, *J* = 8.7, 1.9 Hz, Ar-H), 7.60 (2H, dt, *J* = 8.7, 1.9 Hz, Ar-H), 7.06 (2H, dt, *J* = 8.9, 2.9 Hz, Ar-H), 6.81 (2H, dt, *J* = 9.0, 2.9 Hz, Ar-H).

#### 3.1.2. Synthesis of Compounds **5**–**7**

BFA (56.1 mg, 0.2 mmol) and 4-carboxyphenyl isothiocyanate (53.8 mg, 0.3 mmol) were dissolved in 10 mL of DCM, and EDCI (191.7 mg, 1.0 mmol) and DMAP (2.4 mg, 0.02 mmol) were added to react for 12 h at room temperature. After the reaction was completed, 20 mL of water was added, extracted three times with DCM, the combined DCM was washed once with saturated sodium chloride solution, dried with Na_2_SO_4_, filtered, concentrated under reduced pressure, and gradient eluted with silica gel column chromatography (PE:EA = 10:1–4:1) to obtain compounds **5**–**7**, respectively. The structures of **5**–**7** were characterized by ^1^H NMR, ^13^C NMR, and HR-ESIMS (Figures S7–S15).

##### (1*R*,2*E*,6*S*,10*E*,11a*S*,13*S*,14a*R*)-6-methyl-4-oxo-1,6,7,8,9,11a,12,13,14,14a-decahydro-4*H*-cyclopenta[f][1]oxacyclotridecine-1,13-diyl bis(4-isothiocyanatobenzoate) (**5**)

White oil, yield: 23%. ^1^H NMR (CDCl_3_, 600 MHz), *δ*: 8.05 (2H, d, *J* = 8.6 Hz, Ar-H), 8.00 (2H, d, *J* = 8.6 Hz, Ar-H), 7.35 (1H, dd, *J* = 15.7, 3.4 Hz, H-3), 7.28 (4H, m, Ar-H), 5.79 (2H, m, H-2, H-11), 5.56 (1H, ddd, *J* = 10.4, 3.1, 1.7 Hz, H-4), 5.40 (1H, m, H-7), 5.30 (1H, dd, *J* = 15.1, 9.6 Hz, H-10), 4.88 (1H, m, H-15), 2.67–0.93 (15H, m, H-5, H-6a, H-6b, H-8a, H-8b, H-9, H-12a, H-12b, H-13a, H-13b, H-14a, H-14b, -CH_3_); ^13^C NMR (CDCl_3_, 150 MHz), *δ*: 165.5, 164.8, 164.2, 146.6, 138.1, 138.0, 136.2, 135.8, 135.4, 131.6, 131.2(×2), 131.0 (×2), 128.2, 127.9, 125.8 (×2), 125.7 (×2), 118.8, 77.1, 76.3, 72.0, 50.1, 44.2, 40.1, 38.5, 34.1, 31.8, 26.5, 20.7; HR-ESIMS *m/z* calcd for C_32_H_30_N_2_NaO_6_S_2_ [M + Na]^+^ 625.1437, found 625.1489.

##### (1*R*,2*E*,6*S*,10*E*,11a*S*,13*S*,14a*R*)-13-Hydroxy-6-methyl-4-oxo-1,6,7,8,9,11a,12,13,14,14a-decahydro-4*H*-cyclopenta[f][1]oxacyclotridecin-1-yl 4-isothiocyanatobenzoate (**6**)

White oil, yield: 12%. ^1^H NMR (CDCl_3_, 600 MHz), *δ*: 8.06 (2H, d, *J* = 8.6 Hz, Ar-H), 7.33 (1H, dd, *J* = 15.7, 3.3 Hz, H-3), 7.29 (2H, d, *J* = 8.6 Hz, Ar-H), 5.74 (2H, m, H-2, H-11), 5.56 (1H, ddd, *J* = 10.5, 3.1, 1.9 Hz, H-4), 5.34 (1H, dd, *J* = 15.3, 9.6 Hz, H-10), 4.85 (1H, m, H-15), 4.34 (1H, m, H-7), 2.55–0.90 (15H, m, H-5, H-6a, H-6b, H-8a, H-8b, H-9, H-12a, H-12b, H-13a, H-13b, H-14a, H-14b, -CH_3_); ^13^C NMR (CDCl_3_, 150 MHz), *δ*: 165.6, 164.3, 146.9, 138.1, 136.3, 136.1, 131.2 (×2), 130.8, 128.0, 125.8 (×2), 118.4, 77.3, 72.4, 71.9, 49.7, 44.3, 43.2, 41.1, 34.1, 31.8, 26.6, 20.8; HR-ESIMS *m/z* calcd for C_24_H_27_NNaO_5_S [M + Na]^+^ 464.1502, found 464.1505.

##### (1*R*,2*E*,6*S*,10*E*,11a*S*,13*S*,14a*R*)-1-Hydroxy-6-methyl-4-oxo-1,6,7,8,9,11a,12,13,14,14a-decahydro-4*H*-cyclopenta[f][1]oxacyclotridecin-13-yl 4-isothiocyanatobenzoate (**7**)

White oil, yield: 7%. ^1^H NMR (CDCl_3_, 600 MHz), *δ*: 8.00 (2H, dt, *J* = 8.6, 2.0 Hz, Ar-H), 7.37 (1H, dd, *J* = 15.7, 3.1 Hz, H-3), 7.27 (2H, dt, *J* = 8.6, 2.0 Hz, Ar-H), 5.94 (1H, dd, *J* = 15.7, 1.9 Hz, H-2), 5.75 (1H, m, H-11), 5.40 (1H, m, H-7), 5.24 (1H, dd, *J* = 15.2, 9.4 Hz, H-10), 4.88 (1H, m, H-15), 4.18 (1H, m, H-4), 2.50–0.92 (15H, m, H-5, H-6a, H-6b, H-8a, H-8b, H-9, H-12a, H-12b, H-13a, H-13b, H-14a, H-14b, -CH_3_); ^13^C NMR (CDCl_3_, 150 MHz), *δ*: 166.0, 164.9, 151.1, 137.9, 135.8, 135.7, 131.2, 131.0 (×2), 129.0, 125.7 (×2), 117.9, 76.3, 75.8, 71.7, 52.4, 44.0, 40.2, 38.8, 34.1, 31.8, 26.6, 20.8; HR-ESIMS m/z calcd for C_24_H_27_NNaO_5_S [M + Na]^+^ 464.1502, found 464.1498.

#### 3.1.3. Synthesis of Compounds **8a**–**k** and **9a**–**k**

BFA (56.1 mg, 0.2 mmol) and **4a** (160.5 mg) were dissolved in 10 mL of DCM, EDCI (191.7 mg, 1.0 mmol) and a catalytic amount of DMAP (2.4 mg, 0.02 mmol) were added to react for 12 h at room temperature. After the reaction was completed, 20 mL of water was added, extracted three times with DCM the combined DCM was washed once with saturated sodium chloride solution, dried with Na_2_SO_4_, filtered, concentrated under reduced pressure, and gradient eluted with silica gel column chromatography (DCM:MeOH = 400:1–150:1) to obtain compounds **8a** and **9a**, respectively. All other derivatives were obtained by referring to the synthesis method of compounds **8a** and **9a**. The structures of **8a**–**k** and **9a**–**k** were characterized by ^1^H NMR, ^13^C NMR, and HR-ESIMS (Figures S16–S81).

##### Bis(2-((4-isothiocyanatobenzoyl)oxy)ethyl) *O*,*O*’-((1*R*,2*E*,6*S*,10*E*,11a*S*,13*S*,14a*R*)-6-methyl-4-oxo-1,6,7,8,9,11a,12,13,14,14a-decahydro-4*H*-cyclopenta[f][1]oxacyclotridecine-1,13-diyl) disuccinate (**8a**)

Colorless oil, yield: 25%. ^1^H NMR (CDCl_3_, 600 MHz), *δ*: 8.03 (4H, m, Ar-H), 7.27 (4H, m, Ar-H), 7.20 (1H, dd, *J* = 15.7, 3.4 Hz, H-3), 5.71 (2H, m, H-2, H-11), 5.22 (2H, m, H-4, H-10), 5.10 (1H, m, H-7), 4.85 (1H, m, H-15), 4.51 (4H, m, 2×-COOCH_2_-), 4.44 (4H, m, 2×-COOCH_2_-), 2.64 (8H, m, 4×-COCH_2_-), 2.46–0.89 (15H, m, H-5, H-6a, H-6b, H-8a, H-8b, H-9, H-12a, H-12b, H-13a, H-13b, H-14a, H-14b, -CH_3_); ^13^C NMR (CDCl_3_, 150 MHz), *δ*: 172.1, 171.8, 171.6, 171.1, 165.5, 165.1 (×2), 146.6, 138.0 (×2), 135.9, 135.8, 135.5, 131.3, 131.2 (×4), 128.3 (×2), 125.7 (×4), 118.5, 76.6, 75.6, 71.8, 62.9 (×2), 62.4, 62.3, 49.7, 44.0, 40.0, 38.2, 34.1, 31.8, 29.2, 28.9 (×2), 28.8, 26.5, 20.7; HR-ESIMS *m/z* calcd for C_44_H_46_N_2_NaO_14_S_2_ [M + Na]^+^ 913.2283, found 913.2337.

##### (1*R*,2*E*,6*S*,10*E*,11a*S*,13*S*,14a*R*)-13-Hydroxy-6-methyl-4-oxo-1,6,7,8,9,11a,12,13,14,14a-decahydro-4*H*-cyclopenta[f][1]oxacyclotridecin-1-yl (2-((4-isothiocyanatobenzoyl)oxy)ethyl) succinate (**9a**)

Colorless oil, yield: 18%. ^1^H NMR (CDCl_3_, 600 MHz), *δ*: 8.04 (2H, d, *J* = 8.5 Hz, Ar-H), 7.28 (2H, d, *J* = 8.5 Hz, Ar-H), 7.22 (1H, dd, *J* = 15.8, 3.4 Hz, H-3), 5.70 (2H, m, H-2, H-11), 5.27 (2H, m, H-4, H-10), 4.85 (1H, m, H-15), 4.52 (2H, t, *J* = 4.7 Hz, -COOCH_2_-), 4.45 (2H, m, -COOCH_2_-), 4.30 (1H, m, H-7), 2.70 (4H, m, 2×-COCH_2_-), 2.45–0.89 (15H, m, H-5, H-6a, H-6b, H-8a, H-8b, H-9, H-12a, H-12b, H-13a, H-13b, H-14a, H-14b, -CH_3_); ^13^C NMR (CDCl_3_, 150 MHz), *δ*: 171.8, 171.1, 165.6, 165.2, 146.9, 138.0, 136.3, 135.9, 131.3 (×2), 130.7, 128.3, 125.7 (×2), 118.3, 76.7, 72.3, 71.9, 63.0, 62.3, 49.5, 44.3, 43.1, 40.9, 34.1, 31.8, 29.0, 28.9, 26.6, 20.8; HR-ESIMS *m/z* calcd for C_30_H_35_NNaO_9_S [M + Na]^+^ 608.1925, found 608.1930.

##### Bis(4-((4-isothiocyanatobenzoyl)oxy)butyl) *O*,*O*’-((1*R*,2*E*,6*S*,10*E*,11a*S*,13*S*,14a*R*)-6-methyl-4-oxo-1,6,7,8,9,11a,12,13,14,14a-decahydro-4*H*-cyclopenta[f][1]oxacyclotridecine-1,13-diyl) disuccinate (**8b**)

Colorless oil, yield: 28%. ^1^H NMR (CDCl_3_, 600 MHz), *δ*: 8.02 (4H, m, Ar-H), 7.27 (4H, m, Ar-H), 7.21 (1H, dd, *J* = 15.6, 3.2 Hz, H-3), 5.71 (2H, m, H-2, H-11), 5.24 (2H, m, H-4, H-10), 5.13 (1H, m, H-7), 4.85 (1H, m, H-15), 4.34 (4H, m, 2×-COOCH_2_-), 4.16 (4H, m, 2×-COOCH_2_-), 2.64 (8H, m, 4×-COCH_2_-), 2.47–0.89 (23H, m, H-5, H-6a, H-6b, H-8a, H-8b, H-9, H-12a, H-12b, H-13a, H-13b, H-14a, H-14b, -CH_3_, 4×-CH_2_-); ^13^C NMR (CDCl_3_, 150 MHz), *δ*: 172.2, 172.0, 171.7, 171.2, 165.5, 165.3 (×2), 146.7, 137.9, 137.8, 135.7, 135.6, 135.6, 131.2, 131.0 (×4), 128.8, 128.7, 125.6 (×4), 118.5, 76.6, 75.5, 71.8, 64.8, 64.7, 64.3, 64.1, 49.7, 44.0, 40.0, 38.2, 34.1, 31.7, 29.3, 29.0 (×3), 26.5, 25.3(×4), 20.7; HR-ESIMS *m/z* calcd for C_48_H_54_N_2_NaO_14_S_2_ [M + Na]^+^ 969.2909, found 969.2952.

##### (1*R*,2*E*,6*S*,10*E*,11a*S*,13*S*,14a*R*)-13-Hydroxy-6-methyl-4-oxo-1,6,7,8,9,11a,12,13,14,14a-decahydro-4*H*-cyclopenta[f][1]oxacyclotridecin-1-yl (4-((4-isothiocyanatobenzoyl)oxy)butyl) succinate (**9b**)

Colorless oil, yield: 15%. ^1^H NMR (CDCl_3_, 600 MHz), δ: 8.03 (2H, d, *J* = 8.5 Hz, Ar-H), 7.27 (2H, d, *J* = 8.5 Hz, Ar-H), 7.22 (1H, dd, ***J*** = 15.7, 3.3 Hz, H-3), 5.69 (2H, m, H-2, H-11), 5.29 (2H, m, H-4, H-10), 4.85 (1H, m, H-15), 4.33 (3H, m, H-7, -COOCH_2_-), 4.17 (2H, t, *J* = 6.1 Hz, -COOCH_2_-), 2.67 (4H, m, 2×-COCH_2_-), 2.45–0.89 (19H, m, H-5, H-6a, H-6b, H-8a, H-8b, H-9, H-12a, H-12b, H-13a, H-13b, H-14a, H-14b, -CH_3_, 2×-CH_2_-); ^13^C NMR (CDCl_3_, 150 MHz), δ: 172.0, 171.2, 165.6, 165.4, 147.0, 137.8, 136.3, 135.7, 131.0 (×2), 130.7, 128.8, 125.7 (×2), 118.3, 76.7, 72.3, 71.8, 64.8, 64.2, 49.5, 44.3, 43.1, 40.9, 34.0, 31.8, 29.0 (×2), 26.6, 25.3 (×2), 20.8; HR-ESIMS *m/z* calcd for C_32_H_39_NNaO_9_S [M + Na]^+^ 636.2238, found 636.2252.

##### Bis(4-((4-isothiocyanatobenzoyl)oxy)but-2-yn-1-yl) *O*,*O*’-((1*R*,2*E*,6*S*,10*E*,11a*S*,13*S*,14a*R*)-6-methyl-4-oxo-1,6,7,8,9,11a,12,13,14,14a-decahydro-4*H*-cyclopenta[f][1]oxacyclotridecine-1,13-diyl) disuccinate (**8c**)

Colorless oil, yield: 13%. ^1^H NMR (CDCl_3_, 600 MHz), *δ*: 8.05 (4H, m, Ar-H), 7.28 (4H, m, Ar-H), 7.21 (1H, dd, *J* = 15.7, 3.4 Hz, H-3), 5.71 (2H, m, H-2, H-11), 5.23 (2H, m, H-4, H-10), 5.14 (1H, m, H-7), 4.95 (4H, m, 2×-COOCH_2_C≡), 4.86 (1H, m, H-15), 4.76 (4H, m, 2×-COOCH_2_C≡), 2.69 (6H, m, 3×-COCH_2_-), 2.61 (2H, m, -COCH_2_-), 2.48–0.91 (15H, m, H-5, H-6a, H-6b, H-8a, H-8b, H-9, H-12a, H-12b, H-13a, H-13b, H-14a, H-14b, -CH_3_); ^13^C NMR (CDCl_3_, 150 MHz), *δ*: 171.5 (×2), 171.2, 171.1, 165.5, 164.6 (×2), 146.6, 138.2, 138.1, 136.1 (×2), 135.6, 131.3 (×5), 127.9, 127.8, 125.7 (×4), 118.5, 81.0 (×2), 80.7 (×2), 76.7, 75.6, 71.8, 52.9, 52.8, 52.4, 52.3, 49.7, 44.0, 40.0, 38.2, 34.1, 31.7, 29.2, 28.8 (×3), 26.5, 20.7; HR-ESIMS *m/z* calcd for C_48_H_46_N_2_NaO_14_S_2_ [M + Na]^+^ 961.2283, found 961.2315.

##### (1*R*,2*E*,6*S*,10*E*,11a*S*,13*S*,14a*R*)-13-Hydroxy-6-methyl-4-oxo-1,6,7,8,9,11a,12,13,14,14a-decahydro-4*H*-cyclopenta[f][1]oxacyclotridecin-1-yl (4-((4-isothiocyanatobenzoyl)oxy)but-2-yn-1-yl) succinate (**9c**)

Colorless oil, yield: 32%. ^1^H NMR (CDCl_3_, 600 MHz), *δ*: 8.06 (2H, d, *J* = 8.3 Hz, Ar-H), 7.28 (2H, d, *J* = 8.3 Hz, Ar-H), 7.22 (1H, dd, *J* = 15.7, 3.3 Hz, H-3), 5.69 (2H, m, H-2, H-11), 5.29 (2H, m, H-4, H-10), 4.96 (2H, s, -COOCH_2_C≡), 4.85 (1H, m, H-15), 4.77 (2H, s, -COOCH_2_C≡), 4.31 (1H, m, H-7), 2.71 (4H, m, 2×-COCH_2_-), 2.45–0.90 (15H, m, H-5, H-6a, H-6b, H-8a, H-8b, H-9, H-12a, H-12b, H-13a, H-13b, H-14a, H-14b, -CH_3_); ^13^C NMR (CDCl_3_, 150 MHz), *δ*: 171.3, 171.0, 165.6, 164.7, 146.9, 138.1, 136.3, 136.1, 131.3 (×2), 130.7, 127.9, 125.7 (×2), 118.4, 81.0, 80.7, 72.4, 71.9, 52.9, 52.4, 49.5, 44.3, 43.1, 40.9, 34.1, 31.8, 28.9, 28.8, 26.6, 20.8; HR-ESIMS *m/z* calcd for C_32_H_35_NNaO_9_S [M + Na]^+^ 632.1925, found 632.1942.

##### Bis(2-(2-((4-isothiocyanatobenzoyl)oxy)ethoxy)ethyl) *O*,*O*’-((1*R*,2*E*,6*S*,10*E*,11a*S*,13*S*,14a*R*)-6-methyl-4-oxo-1,6,7,8,9,11a,12,13,14,14a-decahydro-4*H*-cyclopenta[f][1]oxacyclotridecine-1,13-diyl) disuccinate (**8d**)

Colorless oil, yield: 17%. ^1^H NMR (CDCl_3_, 600 MHz), *δ*: 8.04 (4H, m, Ar-H), 7.27 (4H, m, Ar-H), 7.21 (1H, dd, *J* = 15.7, 3.4 Hz, H-3), 5.71 (2H, m, H-2, H-11), 5.23 (2H, m, H-4, H-10), 5.13 (1H, m, H-7), 4.85 (1H, m, H-15), 4.47 (4H, m, 2×-COOCH_2_-), 4.26 (4H, m, 2×-COOCH_2_-), 3.82 (4H, m, 2×-OCH_2_-), 3.73 (4H, m, 2×-OCH_2_-), 2.63 (8H, m, 4×-COCH_2_-), 2.48–0.91 (15H, m, H-5, H-6a, H-6b, H-8a, H-8b, H-9, H-12a, H-12b, H-13a, H-13b, H-14a, H-14b, -CH_3_); ^13^C NMR (CDCl_3_, 150 MHz), *δ*: 172.2, 171.9, 171.6, 171.1, 165.5, 165.3 (×2), 146.7, 137.9, 137.8, 135.8, 135.7, 135.6, 131.2, 131.1 (×4), 128.6, 128.5, 125.7 (×4), 118.5, 76.6, 75.5, 71.8, 69.0 (×4), 64.3, 64.2, 63.8, 63.7, 49.7, 44.0, 40.0, 38.2, 34.1, 31.7, 29.2, 28.9 (×3), 26.5, 20.7; HR-ESIMS *m/z* calcd for C_48_H_54_N_2_NaO_16_S_2_ [M + Na]^+^ 1001.2807, found 1001.2847.

##### (1*R*,2*E*,6*S*,10*E*,11a*S*,13*S*,14a*R*)-13-Hydroxy-6-methyl-4-oxo-1,6,7,8,9,11a,12,13,14,14a-decahydro-4*H*-cyclopenta[f][1]oxacyclotridecin-1-yl (2-(2-((4-isothiocyanatobenzoyl)oxy)ethoxy)ethyl) succinate (**9d**)

Colorless oil, yield: 25%. ^1^H NMR (CDCl_3_, 600 MHz), *δ*: 8.05 (2H, d, *J* = 8.3 Hz, Ar-H), 7.28 (2H, d, *J* = 8.3 Hz, Ar-H), 7.23 (1H, dd, *J* = 16.0, 2.5 Hz, H-3), 5.69 (2H, m, H-2, H-11), 5.28 (2H, m, H-4, H-10), 4.85 (1H, m, H-15), 4.48 (2H, m, -COOCH_2_-), 4.29 (3H, m, H-7, -COOCH_2_-), 3.83 (2H, m, -OCH_2_-), 3.74 (2H, m, -OCH_2_-), 2.67 (4H, m, 2×-COCH_2_-), 2.44–0.92 (15H, m, H-5, H-6a, H-6b, H-8a, H-8b, H-9, H-12a, H-12b, H-13a, H-13b, H-14a, H-14b, -CH_3_); ^13^C NMR (CDCl_3_, 150 MHz), *δ*: 172.0, 171.1, 165.6, 165.4, 147.0, 137.8, 136.3, 135.7, 131.2 (×2), 130.6, 128.5, 125.7 (×2), 118.3, 76.7, 72.3, 71.8, 69.0 (×2), 64.2, 63.7, 49.5, 44.3, 43.1, 41.0, 34.0, 31.8, 29.0, 28.9, 26.6, 20.8; HR-ESIMS *m/z* calcd for C_32_H_39_NNaO_10_S [M + Na]^+^ 652.2187, found 652.2188.

##### Bis(6-((4-isothiocyanatobenzoyl)oxy)hexyl) *O*,*O*’-((1*R*,2*E*,6*S*,10*E*,11a*S*,13*S*,14a*R*)-6-methyl-4-oxo-1,6,7,8,9,11a,12,13,14,14a-decahydro-4*H*-cyclopenta[f][1]oxacyclotridecine-1,13-diyl) disuccinate (**8e**)

Colorless oil, yield: 23%. ^1^H NMR (CDCl_3_, 600 MHz), *δ*: 8.02 (4H, m, Ar-H), 7.27 (4H, m, Ar-H), 7.22 (1H, dd, *J* = 15.7, 3.4 Hz, H-3), 5.71 (2H, m, H-2, H-11), 5.25 (2H, m, H-4, H-10), 5.14 (1H, m, H-7), 4.85 (1H, m, H-15), 4.31 (4H, m, 2×-COOCH_2_-), 4.10 (4H, m, 2×-COOCH_2_-), 2.64 (8H, m, 4×-COCH_2_-), 2.49–0.90 (31H, m, H-5, H-6a, H-6b, H-8a, H-8b, H-9, H-12a, H-12b, H-13a, H-13b, H-14a, H-14b, -CH_3_, 8×-CH_2_-); ^13^C NMR (CDCl_3_, 150 MHz), *δ*: 172.3, 172.0, 171.7, 171.2, 165.5, 165.4 (×2), 146.8, 137.8, 137.7, 135.7, 135.6, 135.5, 131.2, 131.0 (×4), 129.0, 128.9, 125.6 (×4), 118.5, 76.6, 75.5, 71.8, 65.3, 65.2, 64.7, 64.6, 49.7, 44.1, 40.0, 38.2, 34.1, 31.8, 29.3, 29.1, 29.0 (×2), 28.6 (×2), 28.5 (×2), 26.5, 25.7 (×2), 25.6 (×2), 20.7; HR-ESIMS *m/z* calcd for C_52_H_62_N_2_NaO_14_S_2_ [M + Na]^+^ 1025.3535, found 1025.3533.

##### (1*R*,2*E*,6*S*,10*E*,11a*S*,13*S*,14a*R*)-13-Hydroxy-6-methyl-4-oxo-1,6,7,8,9,11a,12,13,14,14a-decahydro-4*H*-cyclopenta[f][1]oxacyclotridecin-1-yl (6-((4-isothiocyanatobenzoyl)oxy)hexyl) succinate (**9e**)

Colorless oil, yield: 26%. ^1^H NMR (CDCl_3_, 600 MHz), *δ*: 8.02 (2H, d, *J* = 8.5 Hz, Ar-H), 7.27 (2H, d, *J* = 8.5 Hz, Ar-H), 7.23 (1H, dd, *J* = 15.7, 3.4 Hz, H-3), 5.69 (2H, m, H-2, H-11), 5.29 (2H, m, H-4, H-10), 4.84 (1H, m, H-15), 4.31 (3H, m, H-7, -COOCH_2_-), 4.11 (2H, t, *J* = 6.1 Hz, -COOCH_2_-), 2.66 (4H, m, 2×-COCH_2_-), 2.45–0.89 (23H, m, H-5, H-6a, H-6b, H-8a, H-8b, H-9, H-12a, H-12b, H-13a, H-13b, H-14a, H-14b, -CH_3_, 4×-CH_2_-); ^13^C NMR (CDCl_3_, 150 MHz), *δ*: 172.1, 171.3, 165.6, 165.5, 147.0, 137.7, 136.3, 135.6, 131.0 (×2), 130.7, 129.0, 125.6 (×2), 118.3, 76.6, 72.3, 71.8, 65.3, 64.7, 49.5, 44.3, 43.1, 40.9, 34.0, 31.8, 29.1, 29.0, 28.5 (×2), 26.6, 25.6 (×2), 20.8; HR-ESIMS *m/z* calcd for C_34_H_43_NNaO_9_S [M + Na]^+^ 664.2551, found 664.2558.

##### Bis(4-((4-isothiocyanatobenzoyl)oxy)phenyl) *O*,*O*’-((1*R*,2*E*,6*S*,10*E*,11a*S*,13*S*,14a*R*)-6-methyl-4-oxo-1,6,7,8,9,11a,12,13,14,14a-decahydro-4*H*-cyclopenta[f][1]oxacyclotridecine-1,13-diyl) disuccinate (**8f**)

Colorless oil, yield: 14%. ^1^H NMR (CDCl_3_, 600 MHz), *δ*: 8.17 (4H, m, Ar-H), 7.33 (4H, m, Ar-H), 7.22 (5H, m, H-3, Ar-H), 7.15 (4H, m, Ar-H), 5.72 (2H, m, H-2, H-11), 5.32 (1H, ddd, *J* = 10.1, 3.1, 1,6 Hz, H-4), 5.23 (1H, dd, *J* = 15.3, 9.6 Hz, H-10), 5.18 (1H, m, H-7), 4.86 (1H, m, H-15), 2.89 (4H, m, 2×-COCH_2_-), 2.79 (2H, m, -COCH_2_-), 2.70 (2H, m, -COCH_2_-), 2.51–0.91 (15H, m, H-5, H-6a, H-6b, H-8a, H-8b, H-9, H-12a, H-12b, H-13a, H-13b, H-14a, H-14b, -CH_3_); ^13^C NMR (CDCl_3_, 150 MHz), *δ*: 171.5, 171.0, 170.8, 170.5, 165.5, 163.8 (×2), 148.2 (×2), 148.1 (×2), 146.6, 138.3 (×2), 136.4 (×2), 135.6, 131.7 (×4), 131.3, 127.8 (×2), 125.9 (×4), 122.5 (×8), 118.6, 76.7, 75.8, 71.8, 49.8, 44.1, 40.0, 38.3, 34.1, 31.7, 29.3 (×2), 29.1, 28.9, 26.5, 20.7; HR-ESIMS *m/z* calcd for C_52_H_46_N_2_NaO_14_S_2_ [M + Na]^+^ 1009.2283, found 1009.2352.

##### (1*R*,2*E*,6*S*,10*E*,11a*S*,13*S*,14a*R*)-13-Hydroxy-6-methyl-4-oxo-1,6,7,8,9,11a,12,13,14,14a-decahydro-4*H*-cyclopenta[f][1]oxacyclotridecin-1-yl (4-((4-isothiocyanatobenzoyl)oxy)phenyl) succinate (**9f**)

Colorless oil, yield: 8%. ^1^H NMR (CDCl_3_, 600 MHz), *δ*: 8.18 (2H, d, *J* = 8.6 Hz, Ar-H), 7.33 (2H, d, *J* = 8.6 Hz, Ar-H), 7.22 (3H, m, H-3, Ar-H), 7.17 (2H, m, Ar-H), 5.71 (2H, m, H-2, H-11), 5.30 (2H, m, H-4, H-10), 4.86 (1H, m, H-15), 4.28 (1H, m, H-7), 2.87 (4H, m, 2×-COCH_2_-), 2.45–0.90 (15H, m, H-5, H-6a, H-6b, H-8a, H-8b, H-9, H-12a, H-12b, H-13a, H-13b, H-14a, H-14b, -CH_3_); ^13^C NMR (CDCl_3_, 150 MHz), *δ*: 171.0, 170.6, 165.6, 163.9, 148.2, 148.1, 146.9, 138.3, 136.4, 136.3, 131.7 (×2), 130.7, 127.8, 125.9 (×2), 122.5 (×4), 118.4, 76.8, 72.3, 71.9, 49.5, 44.3, 43.1, 41.0, 34.1, 31.8, 29.2, 29.0, 26.6, 20.8; HR-ESIMS *m/z* calcd for C_34_H_35_NNaO_9_S [M + Na]^+^ 656.1925, found 656.1943.

##### Bis(2-((4-isothiocyanatobenzoyl)oxy)ethyl) *O*,*O*’-((1*R*,2*E*,6*S*,10*E*,11a*S*,13*S*,14a*R*)-6-methyl-4-oxo-1,6,7,8,9,11a,12,13,14,14a-decahydro-4*H*-cyclopenta[f][1]oxacyclotridecine-1,13-diyl) dimaleate (**8g**)

Colorless oil, yield: 38%. ^1^H NMR (CDCl_3_, 600 MHz), *δ*: 8.03 (4H, m, Ar-H), 7.28 (4H, m, Ar-H), 7.22 (1H, dd, *J* = 15.8, 2.8 Hz, H-3), 6.88 (4H, m, 4×-C=CH-), 5.71 (2H, m, H-2, H-11), 5.37 (1H, d, *J* = 9.8 Hz, H-4), 5.22 (2H, m, H-7, H-10), 4.87 (1H, m, H-15), 4.57 (8H, m, 4×-COOCH_2_-), 2.56–0.91 (15H, m, H-5, H-6a, H-6b, H-8a, H-8b, H-9, H-12a, H-12b, H-13a, H-13b, H-14a, H-14b, -CH_3_); ^13^C NMR (CDCl_3_, 150 MHz), *δ*: 165.3, 165.1 (×2), 164.6, 164.3, 164.2, 163.5, 145.8, 138.1, 138.0, 136.0 (×2), 135.2, 134.3, 133.9, 133.3, 133.0, 131.7, 131.2 (×4), 128.1 (×2), 125.8 (×2), 125.7 (×2), 118.9, 76.4, 72.0, 63.1, 63.0, 62.7 (×2), 49.6, 44.0, 39.9, 38.3, 34.1, 31.8, 26.4, 20.7; HR-ESIMS *m/z* calcd for C_44_H_42_N_2_NaO_14_S_2_ [M + Na]^+^ 909.1970, found 909.2030.

##### (1*R*,2*E*,6*S*,10*E*,11a*S*,13*S*,14a*R*)-13-Hydroxy-6-methyl-4-oxo-1,6,7,8,9,11a,12,13,14,14a-decahydro-4*H*-cyclopenta[f][1]oxacyclotridecin-1-yl (2-((4-isothiocyanatobenzoyl)oxy)ethyl) maleate(**9g**)

Colorless oil, yield: 14%. ^1^H NMR (CDCl_3_, 600 MHz), *δ*: 8.05 (2H, d, *J* = 8.6 Hz, Ar-H), 7.29 (2H, d, *J* = 8.6 Hz, Ar-H), 7.24 (1H, m, H-3), 6.92 (2H, m, 2×-C=CH-), 5.70 (2H, m, H-2, H-11), 5.33 (2H, m, H-4, H-10), 4.86 (1H, m, H-15), 4.58 (4H, m, 2×-COOCH_2_-), 4.33 (1H, m, H-7), 2.50–0.92 (15H, m, H-5, H-6a, H-6b, H-8a, H-8b, H-9, H-12a, H-12b, H-13a, H-13b, H-14a, H-14b, -CH_3_); ^13^C NMR (CDCl_3_, 150 MHz), *δ*: 165.5, 165.1, 164.4, 163.6, 146.3, 138.0, 136.2, 136.0, 133.7, 133.6, 131.2 (×2), 130.8, 128.1, 125.8 (×2), 118.6, 77.5, 72.4, 72.0, 63.1, 62.8, 49.5, 44.3, 43.2, 41.0, 34.1, 31.8, 26.6, 20.8; HR-ESIMS *m/z* calcd for C_30_H_33_NNaO_9_S [M + Na]^+^ 606.1768, found 606.1790.

##### Bis(4-((4-isothiocyanatobenzoyl)oxy)butyl) *O*,*O*’-((1*R*,2*E*,6*S*,10*E*,11a*S*,13*S*,14a*R*)-6-methyl-4-oxo-1,6,7,8,9,11a,12,13,14,14a-decahydro-4*H*-cyclopenta[f][1]oxacyclotridecine-1,13-diyl) dimaleate (**8h**)

Colorless oil, yield: 28%. ^1^H NMR (CDCl_3_, 600 MHz), *δ*: 8.02 (4H, m, Ar-H), 7.26 (5H, m, H-3, Ar-H), 6.87 (4H, m, 4×-C=CH-), 5.73 (2H, m, H-2, H-11), 5.39 (1H, d, *J* = 10.3 Hz, H-4), 5.24 (2H, m, H-7, H-10), 4.87 (1H, m, H-15), 4.33 (8H, m, 4×-COOCH_2_-), 2.56–0.91 (23H, m, H-5, H-6a, H-6b, H-8a, H-8b, H-9, H-12a, H-12b, H-13a, H-13b, H-14a, H-14b, -CH_3_, 4×-CH_2_-); ^13^C NMR (CDCl_3_, 150 MHz), *δ*: 165.3 (×3), 164.8, 164.6, 164.3, 163.6, 145.9, 137.9, 137.8, 135.7 (×2), 135.2, 134.5, 133.8, 133.6, 132.8, 131.6, 131.0 (×4), 128.7 (×2), 125.7 (×4), 118.9, 77.2, 76.3, 72.0, 64.9, 64.8, 64.6 (×2), 49.7, 44.1, 40.0, 38.3, 34.1, 31.7, 26.4, 25.3 (×4), 20.7; HR-ESIMS *m/z* calcd for C_48_H_50_N_2_NaO_14_S_2_ [M + Na]^+^ 965.2596, found 965.2650.

##### (1*R*,2*E*,6*S*,10*E*,11a*S*,13*S*,14a*R*)-13-Hydroxy-6-methyl-4-oxo-1,6,7,8,9,11a,12,13,14,14a-decahydro-4*H*-cyclopenta[f][1]oxacyclotridecin-1-yl (4-((4-isothiocyanatobenzoyl)oxy)butyl) maleate (**9h**)

Colorless oil, yield: 25%. ^1^H NMR (CDCl_3_, 600 MHz), *δ*: 8.03 (2H, d, *J* = 8.6 Hz, Ar-H), 7.26 (3H, m, H-3, Ar-H), 6.89 (2H, m, 2×-C=CH-), 5.70 (2H, m, H-2, H-11), 5.33 (2H, m, H-4, H-10), 4.86 (1H, m, H-15), 4.33 (5H, m, H-7, 2×-COOCH_2_-), 2.50–0.91 (19H, m, H-5, H-6a, H-6b, H-8a, H-8b, H-9, H-12a, H-12b, H-13a, H-13b, H-14a, H-14b, -CH_3_, 2×-CH_2_-); ^13^C NMR (CDCl_3_, 150 MHz), *δ*: 165.5, 165.4, 164.7, 163.8, 146.3, 137.8, 136.2, 135.7, 134.3, 133.0, 131.0 (×2), 130.8, 128.7, 125.7 (×2), 118.5, 77.4, 72.3, 72.0, 64.9, 64.7, 49.5, 44.3, 43.2, 41.0, 34.1, 31.8, 26.6, 25.3 (×2), 20.8; HR-ESIMS *m/z* calcd for C_32_H_37_NNaO_9_S [M + Na]^+^ 634.2081, found 634.2088.

##### Bis(4-((4-isothiocyanatobenzoyl)oxy)but-2-yn-1-yl) *O*,*O*’-((1*R*,2*E*,6*S*,10*E*,11a*S*,13*S*,14a*R*)-6-methyl-4-oxo-1,6,7,8,9,11a,12,13,14,14a-decahydro-4*H*-cyclopenta[f][1]oxacyclotridecine-1,13-diyl) dimaleate (**8i**)

Colorless oil, yield: 26%. ^1^H NMR (CDCl_3_, 600 MHz), *δ*: 8.05 (4H, m, Ar-H), 7.28 (4H, m, Ar-H), 7.23 (1H, *J* = 15.7, 3.5 Hz, H-3), 6.89 (4H, m, 4×-C=CH-), 5.73 (2H, m, H-2, H-11), 5.38 (1H, d, *J* = 10.3, 3.2, 1.7 Hz, H-4), 5.24 (2H, m, H-7, H-10), 4.97 (4H, m, 2×-COOCH_2_C≡), 4.88 (5H, m, H-15, 2×-COOCH_2_C≡), 2.56–0.93 (15H, m, H-5, H-6a, H-6b, H-8a, H-8b, H-9, H-12a, H-12b, H-13a, H-13b, H-14a, H-14b, -CH_3_); ^13^C NMR (CDCl_3_, 150 MHz), *δ*: 165.3, 164.6 (×2), 164.1, 164.0, 163.8, 163.4, 145.8, 138.2, 138.1, 136.2, 136.1, 135.2, 134.7, 133.7, 133.5, 132.6, 131.7, 131.3 (×4), 127.8 (×2), 125.7 (×4), 118.9, 81.3, 81.2, 80.4 (×2), 76.4, 72.0, 53.0, 52.9, 52.8 (×2), 49.7, 44.0, 39.9, 38.4, 34.1, 31.7, 26.4, 20.7; HR-ESIMS *m/z* calcd for C_48_H_42_N_2_NaO_14_S_2_ [M + Na]^+^ 957.1970, found 957.2020.

##### (1*R*,2*E*,6*S*,10*E*,11a*S*,13*S*,14a*R*)-13-Hydroxy-6-methyl-4-oxo-1,6,7,8,9,11a,12,13,14,14a-decahydro-4*H*-cyclopenta[f][1]oxacyclotridecin-1-yl (4-((4-isothiocyanatobenzoyl)oxy)but-2-yn-1-yl) maleate (**9i**)

Colorless oil, yield: 22%. ^1^H NMR (CDCl_3_, 600 MHz), *δ*: 8.06 (2H, dt, *J* = 8.6, 2.0 Hz, Ar-H), 7.28 (2H, dt, *J* = 8.6, 2.0 Hz, Ar-H), 7.25 (1H, m, H-3), 6.93 (2H, m, 2×-C=CH-), 5.70 (2H, m, H-2, H-11), 5.36 (1H, ddd, *J* = 10.5, 3.2, 1.8 Hz, H-4), 5.31 (1H, dd, *J* = 15.2, 9.7 Hz, H-10), 4.98 (2H, t, *J* = 1.7 Hz, -COOCH_2_C≡), 4.87 (3H, m, H-15, -COOCH_2_C≡), 4.33 (1H, m, H-7), 2.49–0.89 (15H, m, H-5, H-6a, H-6b, H-8a, H-8b, H-9, H-12a, H-12b, H-13a, H-13b, H-14a, H-14b, -CH_3_); ^13^C NMR (CDCl_3_, 150 MHz), *δ*: 165.4, 164.6, 163.9, 163.6, 146.2, 138.1, 136.2, 136.1, 133.9, 133.4, 131.3 (×2), 130.9, 127.8, 125.8 (×2), 118.6, 81.3, 80.4, 77.5, 72.3, 72.0, 53.0, 52.8, 49.5, 44.3, 43.2, 41.0, 34.1, 31.8, 26.6, 20.8; HR-ESIMS *m/z* calcd for C_32_H_33_NNaO_9_S [M + Na]^+^ 630.1768, found 630.1790.

##### Bis(2-(2-((4-isothiocyanatobenzoyl)oxy)ethoxy)ethyl) *O*,*O*’-((1*R*,2*E*,6*S*,10*E*,11a*S*,13*S*,14a*R*)-6-methyl-4-oxo-1,6,7,8,9,11a,12,13,14,14a-decahydro-4*H*-cyclopenta[f][1]oxacyclotridecine-1,13-diyl) dimaleate (**8j**)

Colorless oil, yield: 18%. ^1^H NMR (CDCl_3_, 600 MHz), *δ*: 8.03 (4H, m, Ar-H), 7.26 (5H, m, H-3, Ar-H), 6.86 (4H, m, 4×-C=CH-), 5.72 (2H, m, H-2, H-11), 5.39 (1H, d, *J* = 10.3 Hz, H-4), 5.24 (2H, m, H-7, H-10), 4.87 (1H, m, H-15), 4.48 (4H, m, 2×-COOCH_2_-), 4.38 (4H, m, 2×-COOCH_2_-), 3.81 (8H, m, 4×-OCH_2_-), 2.56–0.93 (15H, m, H-5, H-6a, H-6b, H-8a, H-8b, H-9, H-12a, H-12b, H-13a, H-13b, H-14a, H-14b, -CH_3_); ^13^C NMR (CDCl_3_, 150 MHz), *δ*: 165.3 (×3), 164.8, 164.5, 164.2, 163.6, 145.9, 137.9, 137.8, 135.8, 135.7, 135.3, 134.3, 134.0, 133.3, 133.0, 131.7, 131.1 (×4), 128.5 (×2), 125.7 (×4), 118.9, 76.3, 72.0, 69.1 (×2), 68.8 (×2), 64.3, 64.2, 64.1 (×2), 49.7, 44.1, 40.0, 38.3, 34.1, 31.7, 26.5, 20.7; HR-ESIMS *m/z* calcd for C_48_H_50_N_2_NaO_16_S_2_ [M + Na]^+^ 997.2494, found 997.2576.

##### (1*R*,2*E*,6*S*,10*E*,11a*S*,13*S*,14a*R*)-13-Hydroxy-6-methyl-4-oxo-1,6,7,8,9,11a,12,13,14,14a-decahydro-4*H*-cyclopenta[f][1]oxacyclotridecin-1-yl (2-(2-((4-isothiocyanatobenzoyl)oxy)ethoxy)ethyl) maleate (**9j**)

Colorless oil, yield: 29%. ^1^H NMR (CDCl_3_, 600 MHz), *δ*: 8.04 (2H, dt, *J* = 8.6, 2.0 Hz, Ar-H), 7.27 (2H, dt, *J* = 8.6, 2.0 Hz, Ar-H), 7.25 (1H, dd, *J* = 15.6, 3.4 Hz, H-3), 6.90 (2H, m, 2×-C=CH-), 5.70 (2H, m, H-2, H-11), 5.34 (2H, m, H-4, H-10), 4.86 (1H, m, H-15), 4.49 (2H, t, *J* = 4.8 Hz, -COOCH_2_-), 4.40 (2H, t, *J* = 4.7 Hz, -COOCH_2_-), 4.33 (1H, m, H-7), 3.82 (4H, m, 2×-OCH_2_-), 2.49–0.92 (15H, m, H-5, H-6a, H-6b, H-8a, H-8b, H-9, H-12a, H-12b, H-13a, H-13b, H-14a, H-14b, -CH_3_); ^13^C NMR (CDCl_3_, 150 MHz), *δ*: 165.5, 165.3, 164.6, 163.7, 146.3, 137.8, 136.2, 135.7, 134.1, 133.2, 131.1 (×2), 130.8, 128.5, 125.7 (×2), 118.6, 77.4, 72.4, 72.0, 69.1, 68.8, 64.3, 64.2, 49.5, 44.3, 43.1, 41.0, 34.1, 31.8, 26.6, 20.8; HR-ESIMS *m/z* calcd for C_32_H_37_NNaO_10_S [M + Na]^+^ 650.2030, found 650.2050.

##### Bis(6-((4-isothiocyanatobenzoyl)oxy)hexyl) *O*,*O*’-((1*R*,2*E*,6*S*,10*E*,11a*S*,13*S*,14a*R*)-6-methyl-4-oxo-1,6,7,8,9,11a,12,13,14,14a-decahydro-4*H*-cyclopenta[f][1]oxacyclotridecine-1,13-diyl) dimaleate (**8k**)

Colorless oil, yield: 27%. ^1^H NMR (CDCl_3_, 600 MHz), *δ*: 8.02 (4H, m, Ar-H), 7.26 (5H, m, H-3, Ar-H), 6.86 (4H, m, 4×-C=CH-), 5.73 (2H, m, H-2, H-11), 5.38 (1H, ddd, *J* = 10.3, 3.2, 1.7 Hz, H-4), 5.23 (2H, m, H-7, H-10), 4.87 (1H, m, H-15), 4.32 (4H, m, 2×-COOCH_2_-), 4.22 (4H, m, 2×-COOCH_2_-), 2.56–0.93 (31H, m, H-5, H-6a, H-6b, H-8a, H-8b, H-9, H-12a, H-12b, H-13a, H-13b, H-14a, H-14b, -CH_3_, 8×-CH_2_-); ^13^C NMR (CDCl_3_, 150 MHz), *δ*: 165.4 (×2), 165.3, 164.9, 164.7, 164.4, 163.7, 145.9, 137.8, 137.7, 135.6, 135.5, 135.3, 134.7, 133.8, 133.6, 132.6, 131.6, 131.0 (×4), 128.9 (×2), 125.6 (×4), 118.8, 77.1, 76.3, 72.0, 65.4, 65.3, 65.2 (×2), 49.7, 44.1, 40.0, 38.3, 34.1, 31.8, 28.6 (×2), 28.4 (×2), 26.5, 25.7 (×2), 25.6 (×2), 20.7; HR-ESIMS *m/z* calcd for C_52_H_58_N_2_NaO_14_S_2_ [M + Na]^+^ 1021.3222, found 1021.3292.

##### (1*R*,2*E*,6*S*,10*E*,11a*S*,13*S*,14a*R*)-13-Hydroxy-6-methyl-4-oxo-1,6,7,8,9,11a,12,13,14,14a-decahydro-4*H*-cyclopenta[f][1]oxacyclotridecin-1-yl (6-((4-isothiocyanatobenzoyl)oxy)hexyl) maleate (**9k**)

Colorless oil, yield: 21%. ^1^H NMR (CDCl_3_, 600 MHz), *δ*: 8.03 (2H, d, *J* = 8.5 Hz, Ar-H), 7.27 (3H, m, H-3, Ar-H), 6.88 (2H, m, 2×-C=CH-), 5.71 (2H, m, H-2, H-11), 5.33 (2H, m, H-4, H-10), 4.86 (1H, m, H-15), 4.33 (3H, m, H-7, -COOCH_2_-), 4.23 (2H, t, *J* = 6.6 Hz, -COOCH_2_-), 2.49–0.91 (23H, m, H-5, H-6a, H-6b, H-8a, H-8b, H-9, H-12a, H-12b, H-13a, H-13b, H-14a, H-14b, -CH_3_, 4×-CH_2_-); ^13^C NMR (CDCl_3_, 150 MHz), *δ*: 165.5 (×2), 164.7, 163.8, 146.4, 137.7, 136.2, 135.6, 134.5, 132.8, 131.0 (×2), 130.8, 128.9, 125.7 (×2), 118.5, 77.4, 72.3, 72.0, 65.4, 65.2, 49.5, 44.3, 43.1, 41.0, 34.1, 31.8, 28.6, 28.4, 26.6, 25.7, 25.6, 20.8; HR-ESIMS *m/z* calcd for C_34_H_41_NNaO_9_S [M + Na]^+^ 662.2394, found 662.2433.

### 3.2. Cell Viability Analysis

CCK-8 assay was used to detect the cytotoxic activity of all derivatives against HepG2, MDA-MB-231, A375, A549 and HeLa tumor cells and human liver cells L-02. All cells were cultured in Dulbecco’s Modified Eagle’s Medium (DMEM) (Burlington, MA, USA) or Roswell Park Memorial Institute (RPMI)-1640 medium containing 10% fetal bovine serum and 1% penicillin/streptomycin in an incubator at 37 °C and 5% CO_2_. Cells were digested with 0.25% trypsin with 0.02% EDTA (in PBS), and centrifuged to collect cells. Cell suspension with a concentration of 8 × 10^4^ cells/mL was prepared after cell counting. 100 μL cell suspension was added to each well of the 96-well plate and then placed in an incubator overnight. Most of the derivatives were configured into 80 mM, 20 mM or 4 mM stock solution in DMSO, and the stock solution concentration of BFA and paclitaxel was 0.4 mM. The stock solutions were dilute it in the medium (DMSO < 1‰). The medium in each well was discarded, and 100 μL of gradient diluted derivative solution was added (the control group was added with serum-free medium). After 48 h, the medium in each well was discarded, 100 μL serum-free medium containing 10% CCK-8 was added to each well and incubated for 1 h, the absorbance value at 450 nm wavelength was measured by a microplate reader, and the inhibition rate was calculated. The IC_50_ values of the derivatives for each cell were calculated by GraphPad.Prism. v7.0.

### 3.3. Cell Cycle Analysis

Cell cycle was detected by PI single staining method. Cell suspension with a concentration of 2 × 10^5^ cells/mL was prepared after cell counting. 1 mL cell suspension was added to each well of the 6-well plate and then placed in an incubator overnight. The medium in each well was discarded, and 1 mL of gradient diluted derivative solution **6** (0, 0.46, 0.92 and 1.84 µM) was added (the control group was added with serum-free medium). According to the manual of Cell Cycle Detection Kit KGA511, after 48 h, HeLa cells were collected and fixed with 70% ethanol for 2 h. After centrifugation, 100 μL RNase A (working concentration 20 μg/mL) was added and placed in a 37 °C water bath for 30 min. Then 400 μL PI staining (working concentration 50 μg/mL) was added and mixed, centrifugal tube was placed in the dark at 4 °C for 30 min. Red fluorescence at the excitation wavelength of 488 nm was recorded by flow cytometry.

### 3.4. Hoechst 33258 Staining Assay

HeLa cells in 6-well plates were collected after administration. According to the manual of Apoptotic Cell Hoechst 33258 Detection Kit KGA211, HeLa cells were fixed with paraformaldehyde for 30 min. After centrifugation, HeLa cells were soaked in PBS for 5 min and washed 3 times. An appropriate amount of Hoechst 33258 staining solution (working concentration 0.5 μg/mL) was added so that fully covers the HeLa cells and then the cells were left at room temperature for 10 min. After dyeing, HeLa cells were soaked in PBS for 5 min and washed 3 times. Observation under an Olympus (Tokyo, Japan) IX73 fluorescence microscope after sealing with an anti-fluorescence quenching sealing solution (glycerol:PBS = 1:9).

### 3.5. Cell Apoptosis Analysis

According to the manual of Annexin V-FITC/PI Apoptosis Detection Kit KGA105, HeLa cells in 6-well plates were collected by 0.25% trypsin without EDTA (in PBS) digestion after 48 h administration by the above method. After washing with PBS 3 times, 500 μL Binding Buffer was added to suspend HeLa cells. Then Annexin V-FITC and PI were added successively in dark for 15 min, and HeLa cell apoptosis was detected by BD FACSVerse flow cytometer.

### 3.6. Mitochondrial Membrane Potential Analysis

Mitochondrial membrane potential was detected by the JC-1 staining method. According to the manual of JC-1 Apoptosis Detection Kit KGA601, HeLa cells in 6-well plates were collected by 0.25% trypsin without EDTA (in PBS) digestion after 48 h administration. The Incubation Buffer was prepared according to the instructions, mixing and preheating to 37 °C. 500 μL 1 × Incubation Buffer was mixed with 1 μL JC-1 (working concentration 10 μg/mL) to suspend HeLa cells and then the cells were incubated for 20 min. Cells were collected by centrifugation at room temperature, washed twice with 1 × Incubation Buffer and then detected by BD FACSVerse flow cytometer.

## 4. Conclusions

By using BFA as a lead compound, 25 novel BFA-isothiocyanate derivatives **5**, **6**, **7**, **8a**–**k** and **9a**–**k** were designed and synthesized. All derivatives were tested for their cytotoxic activity against HepG2, MDA-MB-231, A375, A549 and HeLa tumor cell lines and human liver cells L-02. Their selectivity index between L-02 and HeLa was generally good, suggesting good selectivity. Among them, **6** showed an antiproliferative effect against HeLa cells with an IC_50_ value of 1.84 μM, and showed the best selectivity between L-02 and HeLa (SI_HeLa_ > 43) with an IC_50_ value to L-02 cells greater than 80 μM. Further cellular mechanism tests found that **6** induced HeLa cell cycle arrest in G1 phase, cell nucleus fragmentation, and decreased mitochondrial membrane potential in HeLa cells. In short, **6** might induce apoptosis of HeLa cells through a mitochondrial-dependent pathway. Therefore, as a candidate anticancer compound, **6** is worthy of further research such as exploring its anticancer signaling pathways and anticancer activity and safety in vivo.

## Figures and Tables

**Table 1:** The antiproliferative effects of the target compounds, parent compounds and positive control against different cell lines.

Compound	^a^ IC_50_ (μM)						^b^ SI_HeLa_
	HepG2	MDA-MB-231	A375	A549	HeLa	L-02	
BFA	0.12 ± 0.01	0.054 ± 0.001	0.039 ± 0.001	0.056 ± 0.002	0.044 ± 0.004	0.094 ± 0.004	2.14
paclitaxel	0.55 ± 0.03	0.33 ± 0.03	0.059 ± 0.001	0.014 ± 0.002	0.034 ± 0.002	0.13 ± 0.00	3.82
**2**	>100	82.06 ± 2.70	42.99 ± 3.34	79.25 ± 2.52	45.73 ± 1.74	67.82 ± 2.00	1.48
**5**	>40	>40	>40	20.9 ± 1.42	>40	>80	^c^ NC
**6**	20.76 ± 1.22	6.83 ± 0.61	3.08 ± 0.43	7.18 ± 0.51	1.84 ± 0.08	>80	>43.48
**7**	>40	17.03 ± 1.21	5.78 ± 0.10	20.35 ± 1.31	3.02 ± 0.23	8.60 ± 0.71	2.85
**8a**	>40	>40	>40	>40	11.98 ± 1.47	>80	>6.68
**8b**	>40	>40	>40	>40	16.41 ± 1.35	>80	>4.88
**8c**	>40	>40	>40	>40	39.03 ± 1.43	>80	>2.05
**8d**	>40	>40	22.77 ± 1.58	>40	7.67 ± 0.33	>80	>10.43
**8e**	>40	>40	>40	>40	26.78 ± 0.70	>80	>2.99
**8f**	>40	>40	>40	>40	>40	>80	^c^ NC
**8g**	>40	>40	>40	>40	9.74 ± 0.37	>80	>8.21
**8h**	>40	>40	>40	>40	8.48 ± 0.72	>80	>9.43
**8i**	>40	>40	>40	>40	>40	>80	^c^ NC
**8j**	>40	>40	>40	23.79 ± 0.02	30.59 ± 2.12	21.27 ± 0.16	0.70
**8k**	>40	>40	>40	>40	>40	>80	^c^ NC
**9a**	5.93 ± 0.03	8.04 ± 0.01	2.07 ± 0.09	8.29 ± 0.10	2.69 ± 0.30	15.48 ± 1.22	5.75
**9b**	6.54 ± 0.18	2.23 ± 0.04	2.79 ± 0.25	2.84 ± 0.02	2.28 ± 0.04	10.56 ± 0.60	4.63
**9c**	8.84 ± 0.28	9.77 ± 0.22	10.41 ± 0.16	13.19 ± 0.03	3.63 ± 0.15	17.37 ± 0.44	4.79
**9d**	2.34 ± 0.31	2.05 ± 0.27	2.93 ± 0.12	2.85 ± 0.10	0.99 ± 0.02	9.18 ± 0.13	9.27
**9e**	5.77 ± 0.07	1.56 ± 0.05	3.87 ± 0.35	2.59 ± 0.13	0.81 ± 0.03	7.87 ± 0.33	9.72
**9f**	27.73 ± 0.47	32.08 ± 1.03	21.31 ± 0.33	16.18 ± 0.06	13.12 ± 0.45	25.1 ± 0.82	1.91
**9g**	12.8 ± 0.15	7.51 ± 0.07	11.88 ± 0.54	10.42 ± 0.59	3.63 ± 0.23	20.88 ± 0.49	5.75
**9h**	13.27 ± 0.10	5.71 ± 0.20	11.2 ± 0.29	8.54 ± 0.26	9.68 ± 0.24	27.81 ± 1.39	2.87
**9i**	11.87 ± 0.32	10.00 ± 0.26	9.10 ± 0.46	10.3 ± 0.35	6.59 ± 0.75	28.14 ± 0.14	4.27
**9j**	3.69 ± 0.14	2.27 ± 0.03	1.5 ± 0.05	4.61 ± 0.12	1.71 ± 0.02	10.76 ± 0.57	6.29
**9k**	3.28 ± 0.27	2.61 ± 0.08	7.27 ± 0.57	8.34 ± 0.24	2.39 ± 0.10	23.95 ± 1.23	10.02

^a^ IC_50_: half inhibitory concentrations measured by the Cell Counting Kit-8 (CCK-8) assay. The values are expressed as average ± standard deviation of three independent experiments. ^b^ SI_HeLa_: selectivity index between L-02 and HeLa. It was calculated as: SI = IC_50(L-02)_/IC_50(HeLa)_. ^c^ NC: not calculated.

## Data Availability

Data are contained within the article or Supplementary Materials.

## Supplementary Materials

The following supporting information can be downloaded at: https://www.mdpi.com/article/10.3390/molecules28114284/s1, Figures S1–S6: ^1^H NMR of **3a**–**f**, Figures S7–S81: ^1^H NMR, ^13^C NMR and HR-ESIMS of compounds **5**, **6**, **7**, **8a**–**k** and **9a**–**k**.

## Abbreviations

AITC: allyl isothiocyanate; ARF1, ADP-ribosylation factor 1; BFA, brefeldin A; BITC, benzyl isothiocyanate; CCK-8, Cell Counting Kit-8; DCM, dichloromethane; DMAP, 4-dimethylaminopyridine; DMEM, Dulbecco’s Modified Eagle’s Medium; DMSO, dimethyl sulfoxide; EA, ethyl acetate; EDCI, *N*-(3-dimethylaminopropyl)-*N*’’-ethylcarbodiimidehydrochloride; EDTA, ethylenediaminetetraacetic acid; GEFs, guanine nucleotide exchange factors; FITC, fluorescein isothiocyanate; H_2_S, hydrogen sulfide; ITCs, isothiocyanates; JC-1, 5,5′,6,6′-tetrachloro-1,1′,3,3′-tetraethylbenzimi-dazolylcarbocyanine iodide; NMR, nuclear magnetic resonance; NRF2, nuclear factor erythroid-2 related factor 2; PE, petroleum ether; PEITC, phenethyl isothiocyanate; PI, propidium iodide; ROS, reactive oxygen species; rt, room temperature; RPMI, Roswell Park Memorial Institute; SFN, sulforaphane; SI, selectivity index; TEA, triethylamine; TMS, tetramethylsilane.
