# Supplementary material for: Design and Synthesis of Brefeldin A-Isothiocyanate Derivatives with Selectivity and Their Potential for Cervical Cancer Therapy
Source: Molecules. 2023 May 23;28(11):4284. doi: 10.3390/molecules28114284 (PMC10254530; doi:10.3390/molecules28114284)
Supplement: Supplementary file 1 [file molecules-28-04284-s001.zip › molecules-2395884-supplementary.pdf]

Design and synthesis brefeldin A-isothiocyanate derivatives with high selectivity and their potential for cervical cancer therapy

Mingying Wang[a], Xiaoyuan Chen[a], Ying Qu[a], Qingyinglu Ma[a], Huaqi Pan[b], Haonan Li[a], Huiming Hua[a,*], Dahong Li[a,*]

[a]*Key Laboratory of Structure-Based Drug Design & Discovery, Ministry of Education, and School of Traditional Chinese Materia Medica, Shenyang Pharmaceutical University, 103 Wenhua Road, Shenyang 110016, China*

[b]*Institute of Applied Ecology, Chinese Academy of Sciences, Shenyang, 110016, China*

[*]*Corresponding author. E-mail address: lidahong0203@163.com (D. Li); huimhua@163.com (H. Hua)*

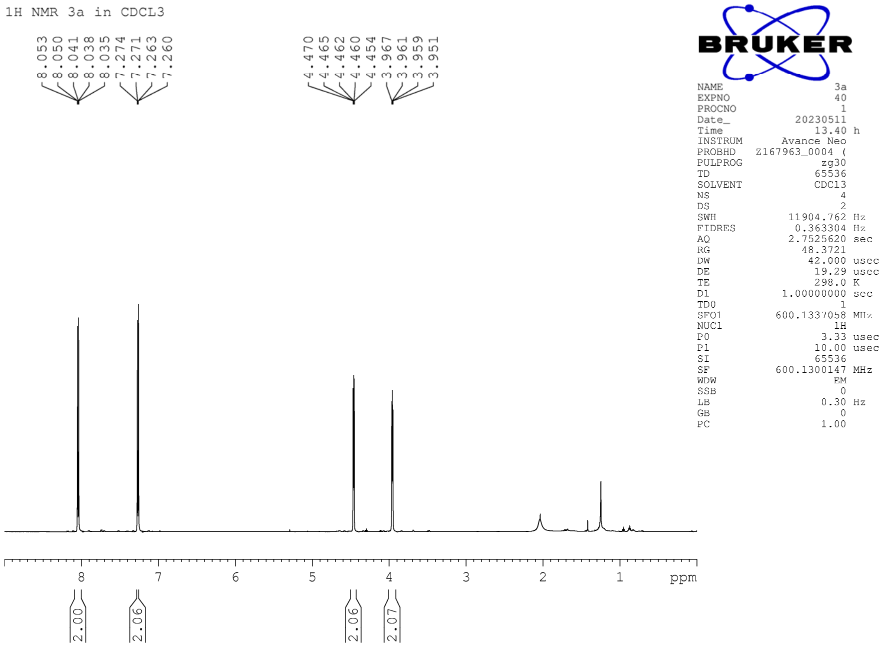

Figure S1. ^{1}H NMR of compound **3a.**

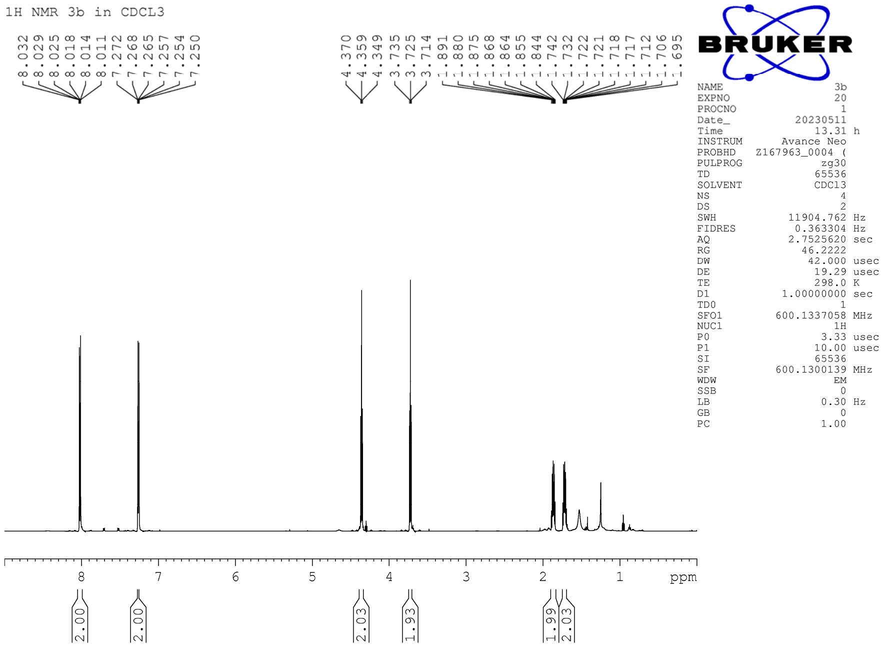

Figure S2. ^{1}H NMR of compound **3b.**

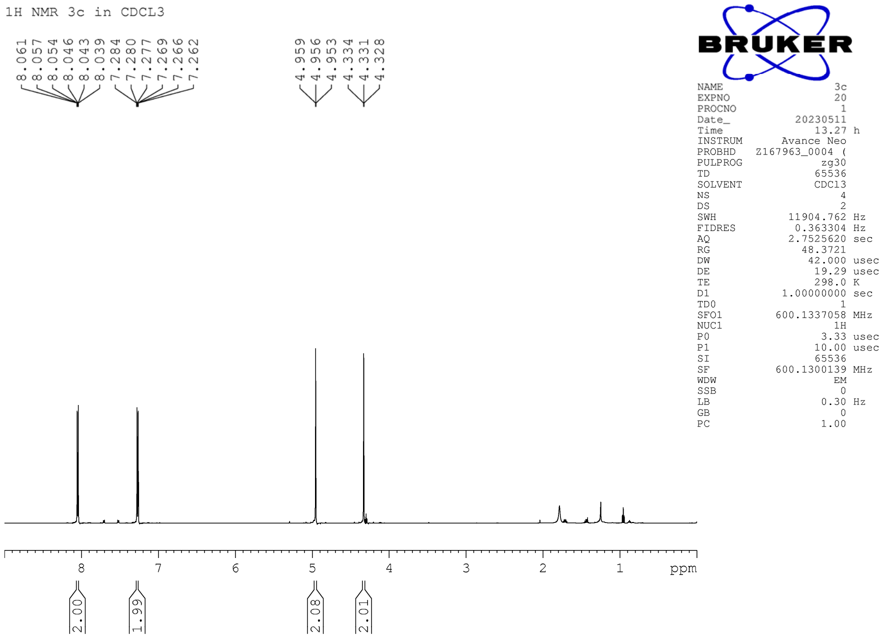

Figure S3. ^{1}H NMR of compound **3c.**

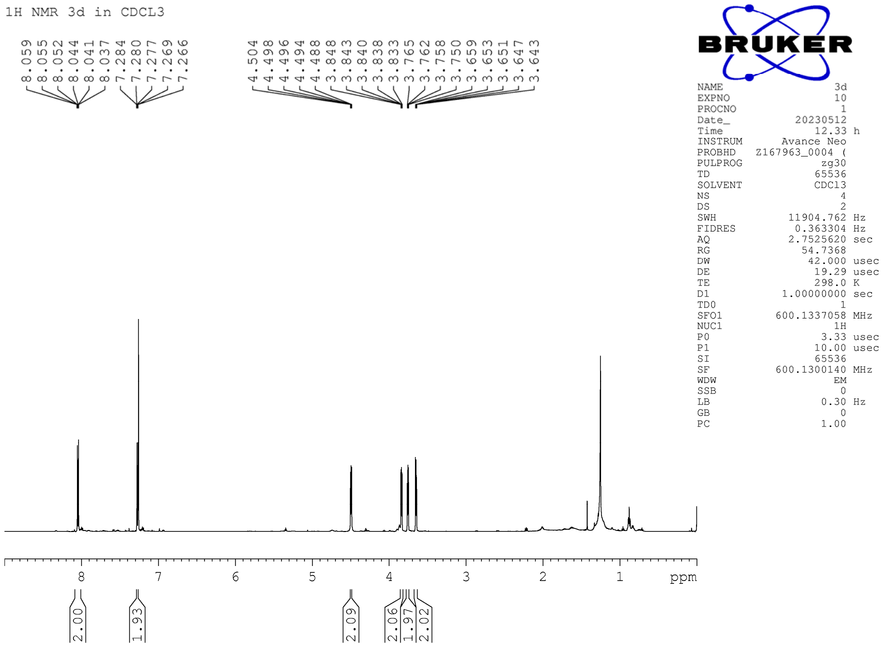

Figure S4. ^{1}H NMR of compound **3d.**

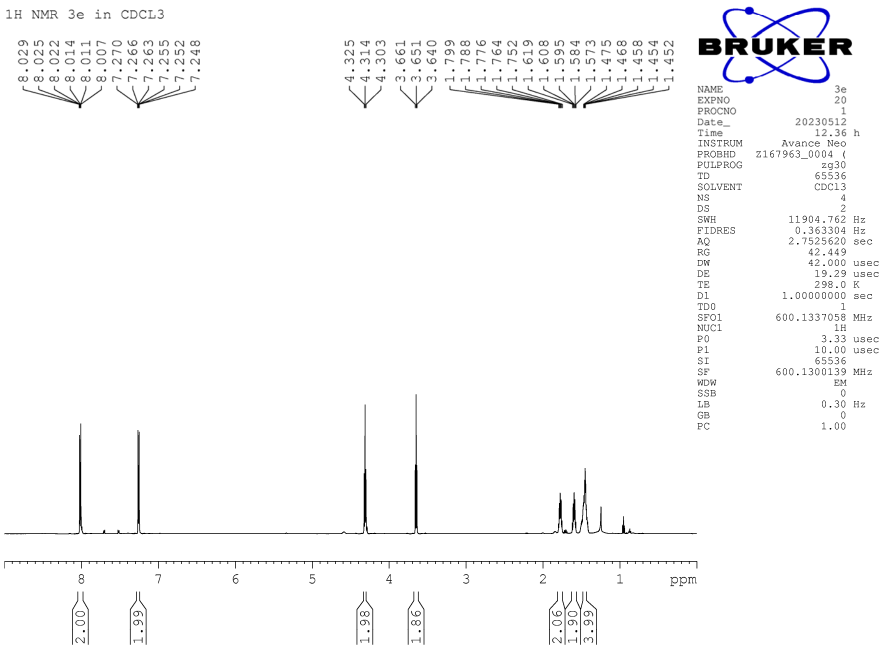

Figure S5. ^{1}H NMR of compound **3e.**

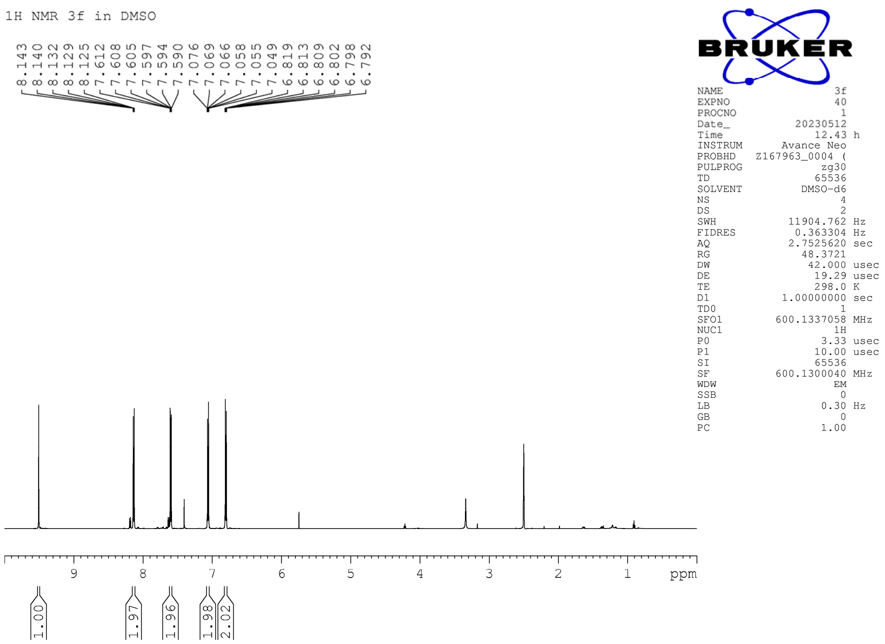

Figure S6. ^{1}H NMR of compound **3f.**

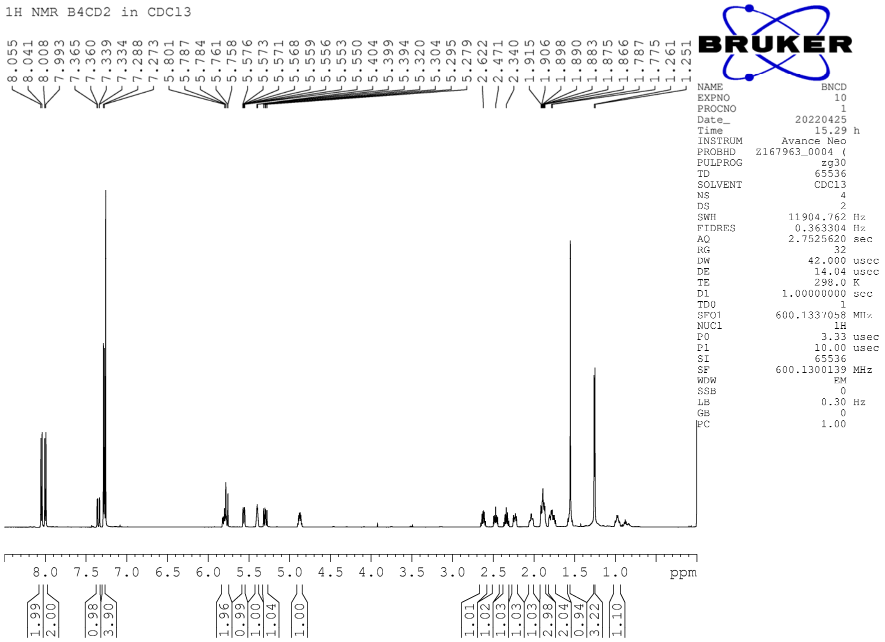

Figure S7. ^{1}H NMR of compound **5.**

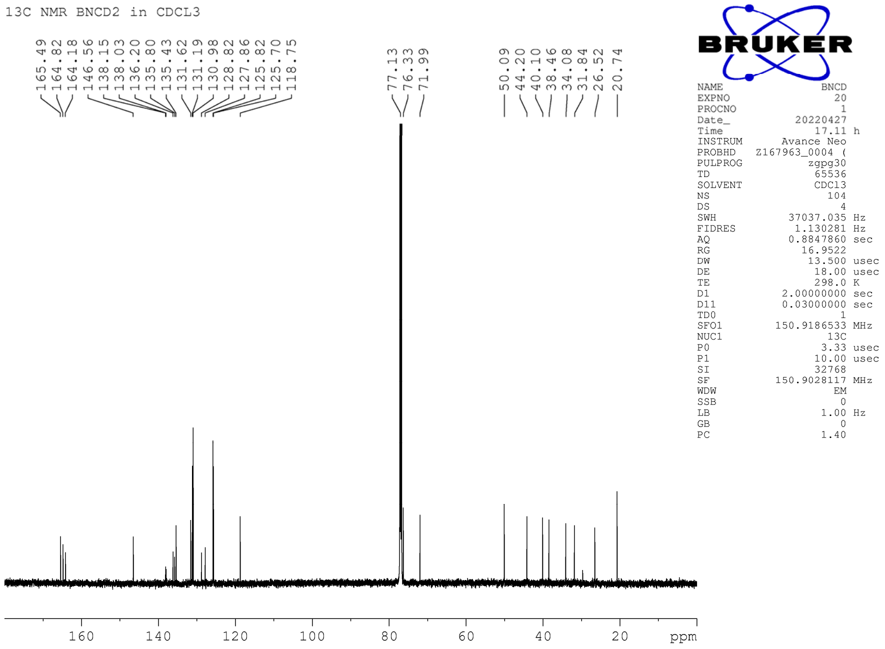

Figure S8. ^{13}C NMR of compound **5.**

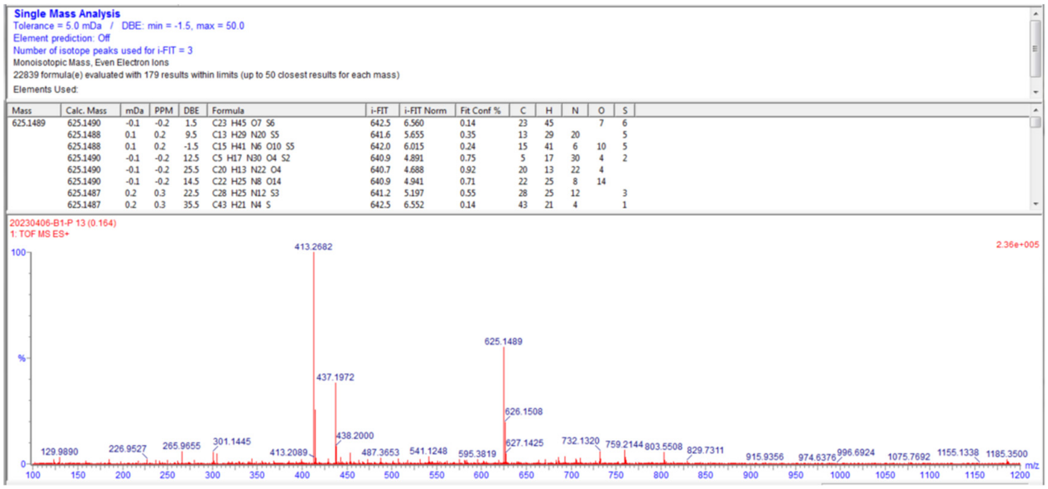

Mass	Calc. Mass	mDa	PPM	DBE	Formula	i-FIT	i-FIT Norm	Fit Conf %	C	H	N	O	S
625.1489	625.1490	-0.1	-0.2	1.5	C23 H45 O7 S6	642.5	6.560	0.14	23	45		7	6
	625.1488	0.1	0.2	9.5	C13 H29 N20 S5	641.6	5.655	0.35	13	29	20		5
	625.1488	0.1	0.2	-1.5	C15 H41 N6 O10 S5	642.0	6.015	0.24	15	41	6	10	5
	625.1490	-0.1	-0.2	12.5	C5 H17 N30 O4 S2	640.9	4.891	0.75	5	17	30	4	2
	625.1490	-0.1	-0.2	25.5	C20 H13 N22 O4	640.7	4.688	0.92	20	13	22	4	
	625.1490	-0.1	-0.2	14.5	C22 H25 N8 O14	640.9	4.941	0.71	22	25	8	14	
	625.1487	0.2	0.3	22.5	C28 H25 N12 S3	641.2	5.197	0.55	28	25	12		3
	625.1487	0.2	0.3	35.5	C43 H21 N4 S	642.5	6.552	0.14	43	21	4		1

Figure S9. HR-ESIMS of compound **5.**

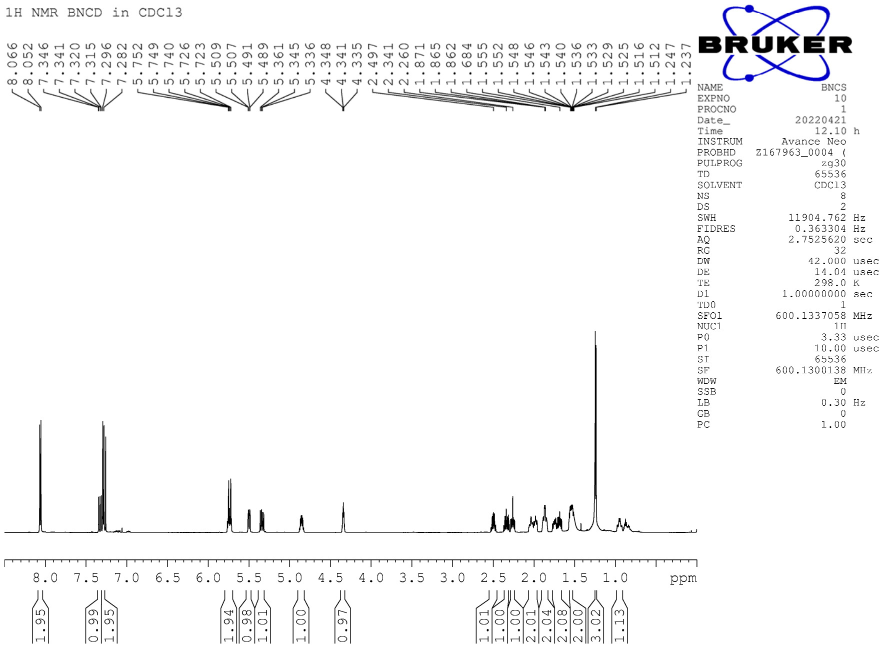

Figure S10. ^{1}H NMR of compound **6.**

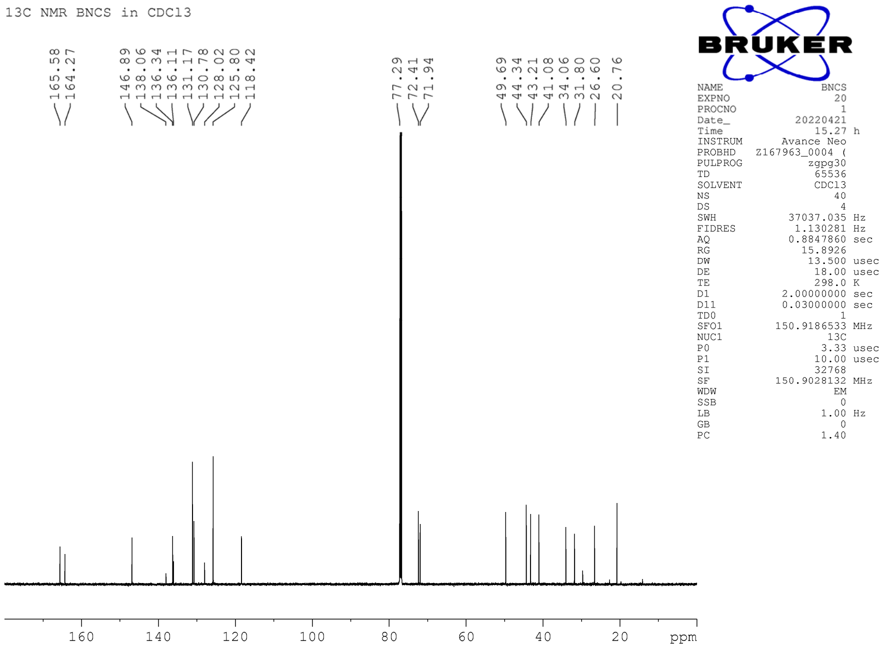

Figure S11. ^{13}C NMR of compound **6.**

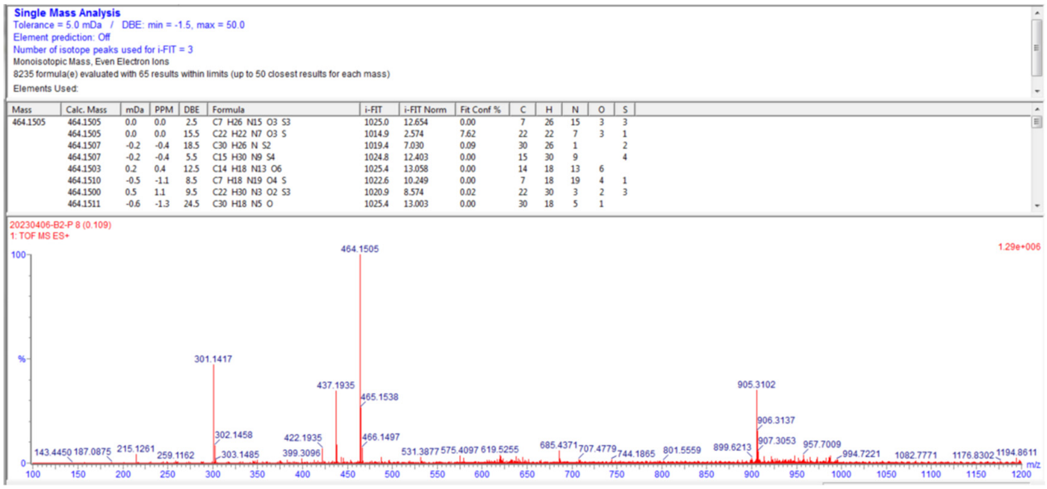
Single Mass Analysis
Tolerance = 5.0 mDa / DBE: min = -1.5, max = 50.0
Element prediction: Off
Number of isotope peaks used for i-FIT = 3
Monoisotopic Mass, Even Electron Ions
8235 formula(e) evaluated with 65 results within limits (up to 50 closest results for each mass)
Elements Used:

Mass	Calc. Mass	mDa	PPM	DBE	Formula	i-FIT	i-FIT Norm	Fit Conf %	C	H	N	O	S
464.1505	464.1505	0.0	0.0	2.5	C7 H26 N15 O3 S3	1025.0	12.654	0.00	7	26	15	3	3
	464.1505	0.0	0.0	15.5	C22 H22 N7 O3 S	1014.9	2.574	7.62	22	22	7	3	1
	464.1507	-0.2	-0.4	18.5	C30 H26 N S2	1019.4	7.030	0.09	30	26	1		2
	464.1507	-0.2	-0.4	5.5	C15 H30 N9 S4	1024.8	12.403	0.00	15	30	9		4
	464.1503	0.2	0.4	12.5	C14 H18 N13 O6	1025.4	13.058	0.00	14	18	13	6	
	464.1510	-0.5	-1.1	8.5	C7 H18 N19 O4 S	1022.6	10.249	0.00	7	18	19	4	1
	464.1500	0.5	1.1	9.5	C22 H30 N3 O2 S3	1020.9	8.574	0.02	22	30	3	2	3
	464.1511	-0.6	-1.3	24.5	C30 H18 N5 O	1025.4	13.003	0.00	30	18	5	1	

Figure S12. HR-ESIMS of compound **6.**

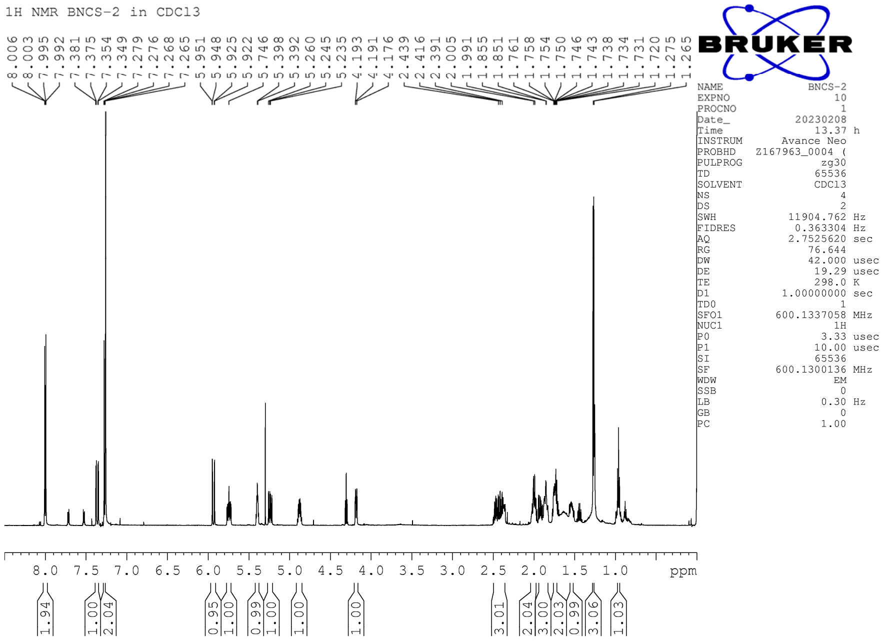

Figure S13. ^{1}H NMR of compound **7.**

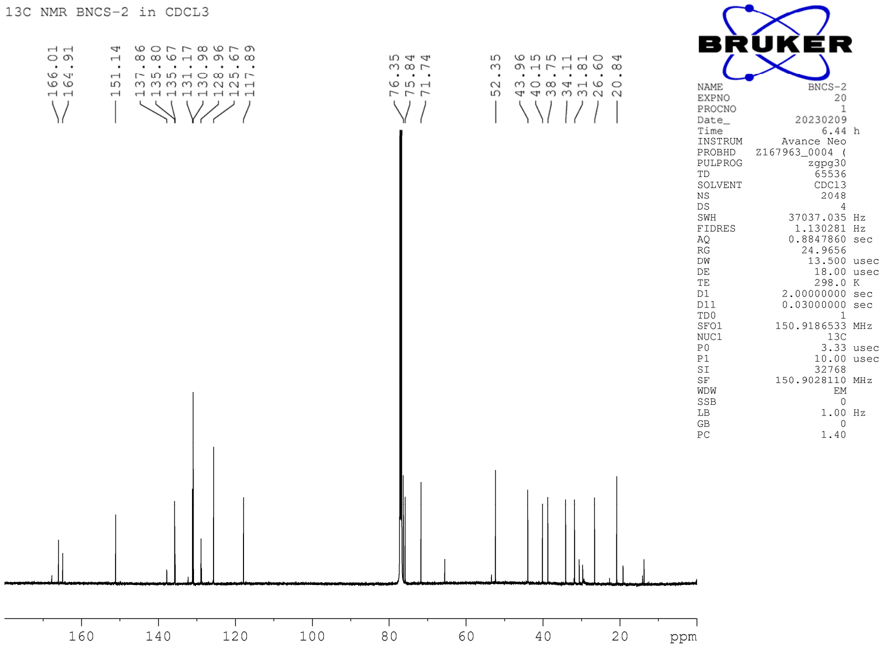

Figure S14. ^{13}C NMR of compound **7.**

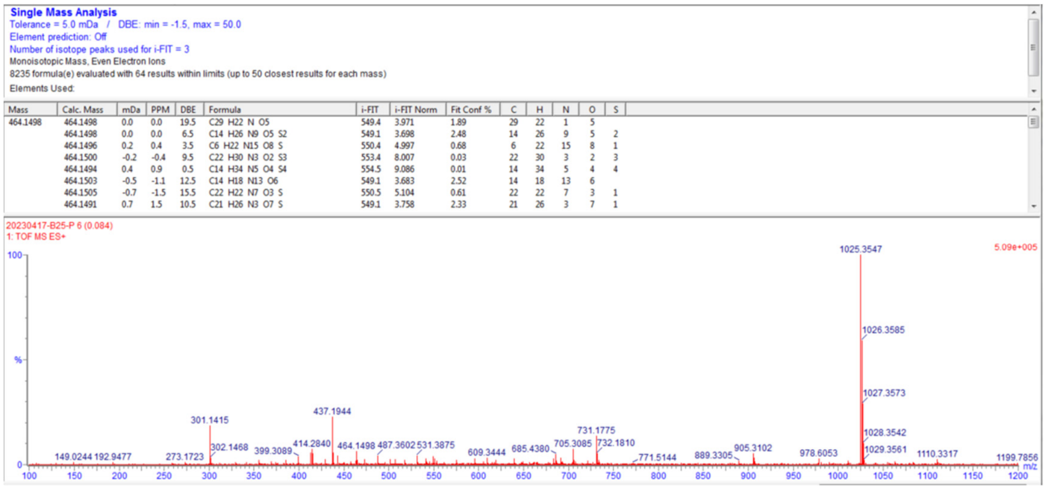

Figure S15. HR-ESIMS of compound **7.**

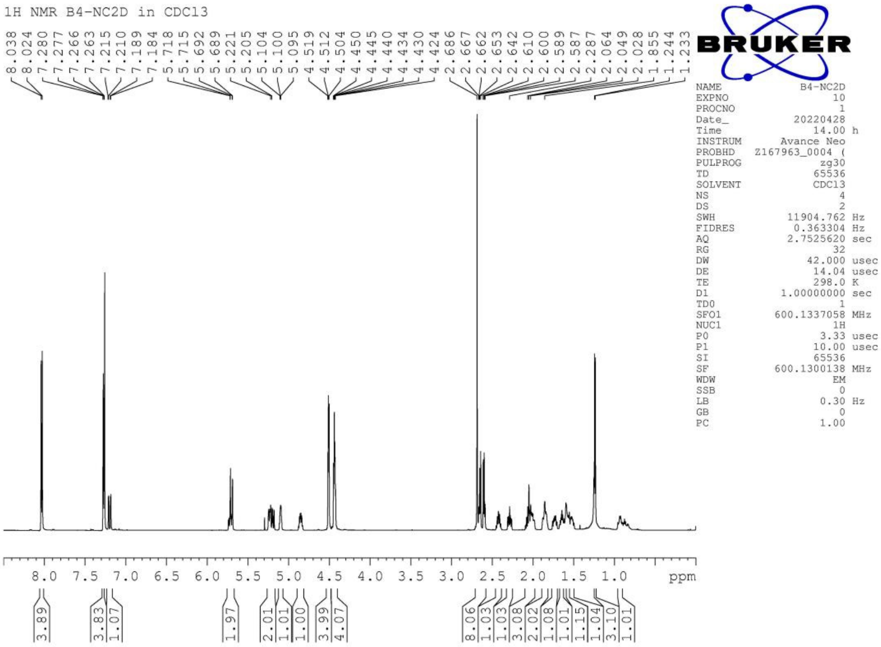

Figure S16. ^{1}H NMR of compound **8a.**

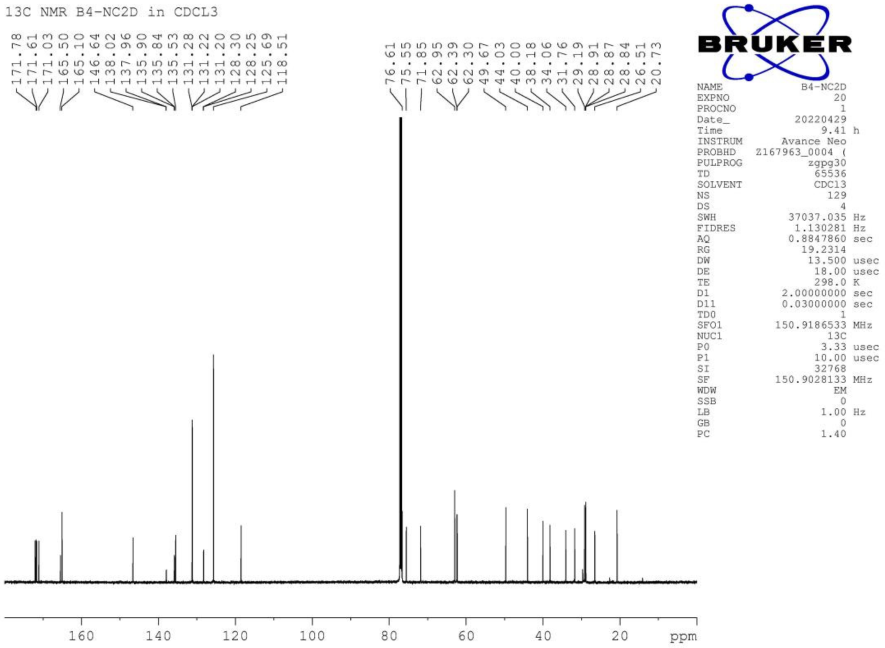

Figure S17. ^{13}C NMR of compound **8a.**

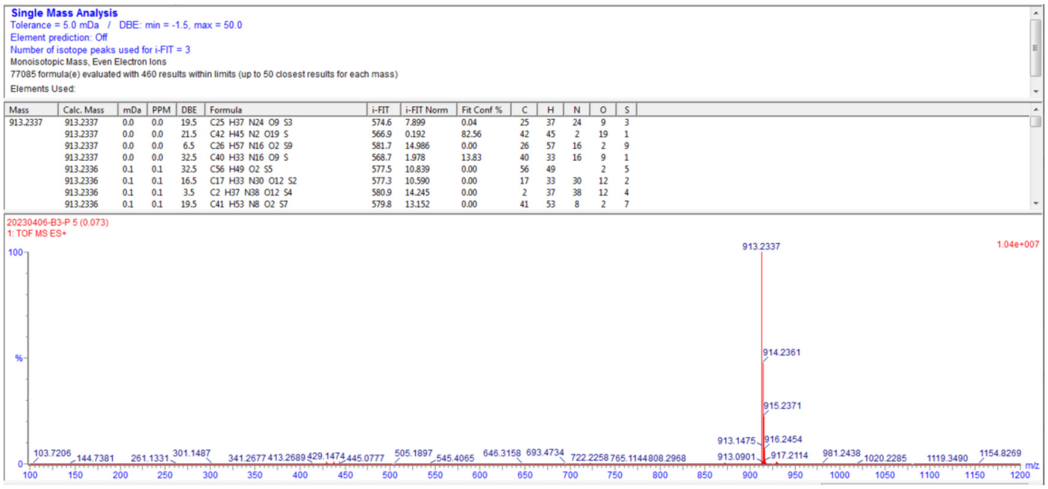

Mass	Calc. Mass	mDa	PPM	DBE	Formula	i-FIT	i-FIT Norm	Fit Conf %	C	H	N	O	S
913.2337	913.2337	0.0	0.0	19.5	C25 H37 N24 O9 S3	574.6	7.899	0.04	25	37	24	9	3
	913.2337	0.0	0.0	21.5	C42 H45 N2 O19 S	566.9	0.192	82.56	42	45	2	19	1
	913.2337	0.0	0.0	6.5	C26 H57 N16 O2 S9	581.7	14.986	0.00	26	57	16	2	9
	913.2337	0.0	0.0	32.5	C40 H33 N16 O9 S	568.7	1.978	13.83	40	33	16	9	1
	913.2336	0.1	0.1	32.5	C56 H49 O2 S5	577.5	10.839	0.00	56	49		2	5
	913.2336	0.1	0.1	16.5	C17 H33 N30 O12 S2	577.3	10.590	0.00	17	33	30	12	2
	913.2336	0.1	0.1	3.5	C2 H37 N38 O12 S4	580.9	14.245	0.00	2	37	38	12	4
	913.2336	0.1	0.1	19.5	C41 H53 N8 O2 S7	579.8	13.152	0.00	41	53	8	2	7

Figure S18. HR-ESIMS of compound **8a.**

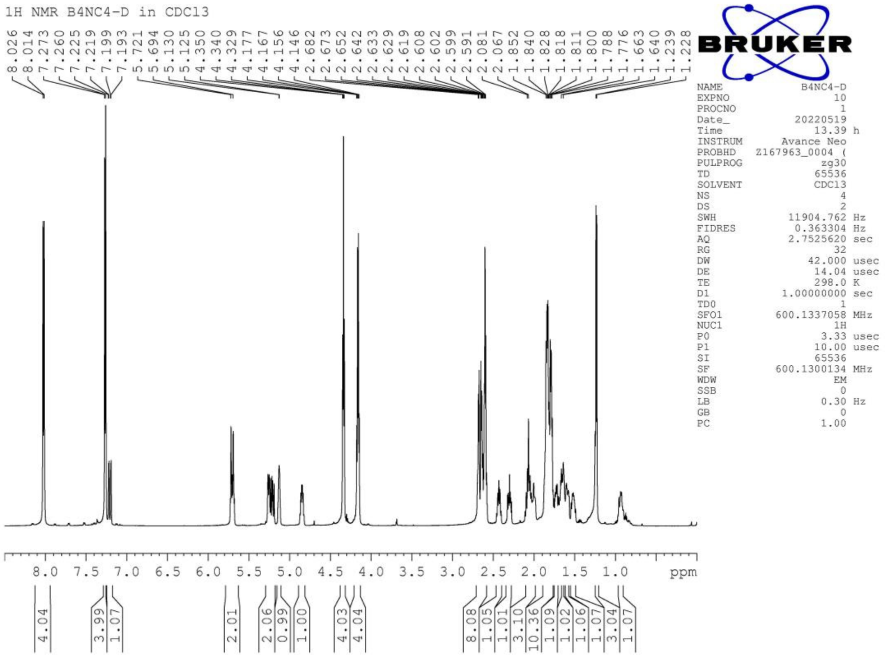

Figure S19. ^{1}H NMR of compound **8b.**

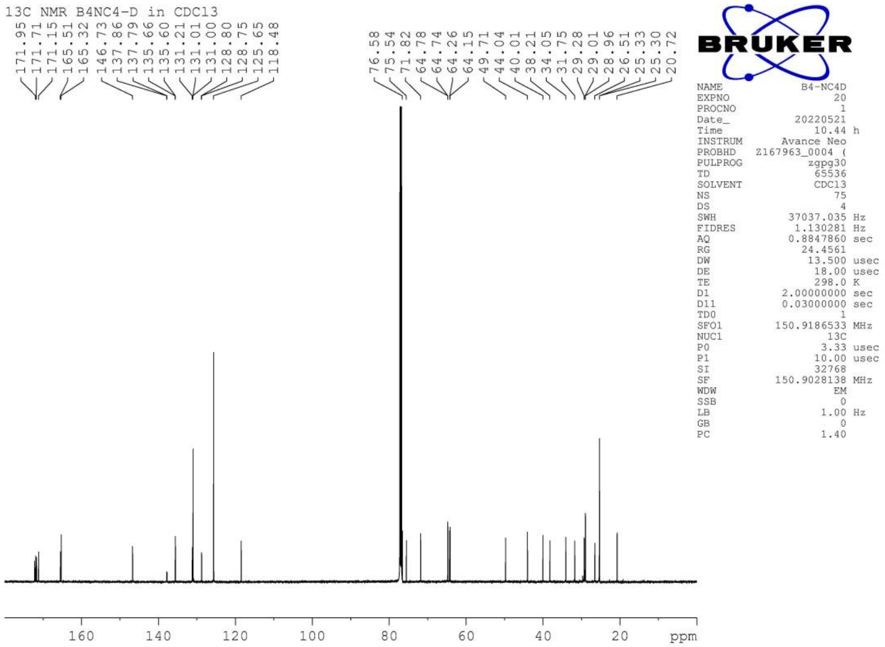

Figure S20. ^{13}C NMR of compound **8b.**

Single Mass Analysis
Tolerance = 5.0 mDa / DBE: min = -1.5, max = 50.0
Element prediction: Off
Number of isotope peaks used for i-FIT = 3
Monoisotopic Mass, Even Electron Ions
91414 formula(e) evaluated with 481 results within limits (up to 50 closest results for each mass)
Elements Used:

Mass	Calc. Mass	mDa	PPM	DBE	Formula	i-FIT	i-FIT Norm	Fit Conf %	C	H	N	O	S
969.2952	969.2952	0.0	0.0	30.5	C51 H49 N6 O10 S2	580.0	4.838	0.79	51	49	6	10	2
	969.2952	0.0	0.0	17.5	C36 H53 N14 O10 S4	584.5	9.289	0.01	36	53	14	10	4
	969.2952	0.0	0.0	4.5	C21 H57 N22 O10 S6	588.1	12.900	0.00	21	57	22	10	6
	969.2952	0.0	0.0	2.5	C4 H49 N44 S8	590.2	14.979	0.00	4	49	44		8
	969.2952	0.0	0.0	28.5	C34 H41 N28 S4	584.1	8.865	0.01	34	41	28		4
	969.2952	0.0	0.0	6.5	C38 H65 O20 S4	584.9	9.735	0.01	38	65		20	4
	969.2952	0.0	0.0	15.5	C19 H45 N36 S6	587.8	12.647	0.00	19	45	36		6
	969.2953	-0.1	-0.1	33.5	C59 H53 O7 S3	583.4	8.233	0.03	59	53		7	3

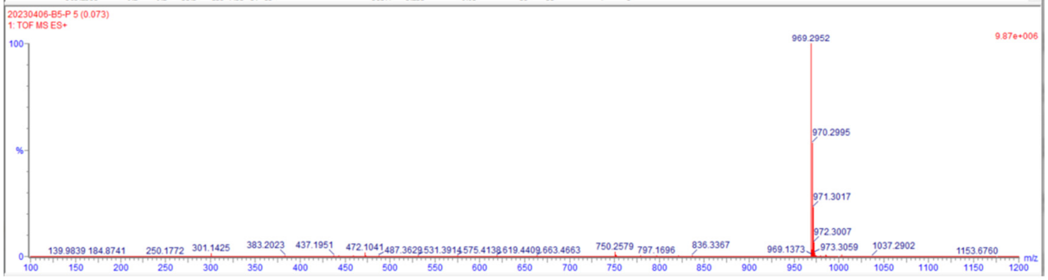

Figure S21. HR-ESIMS of compound **8b.**

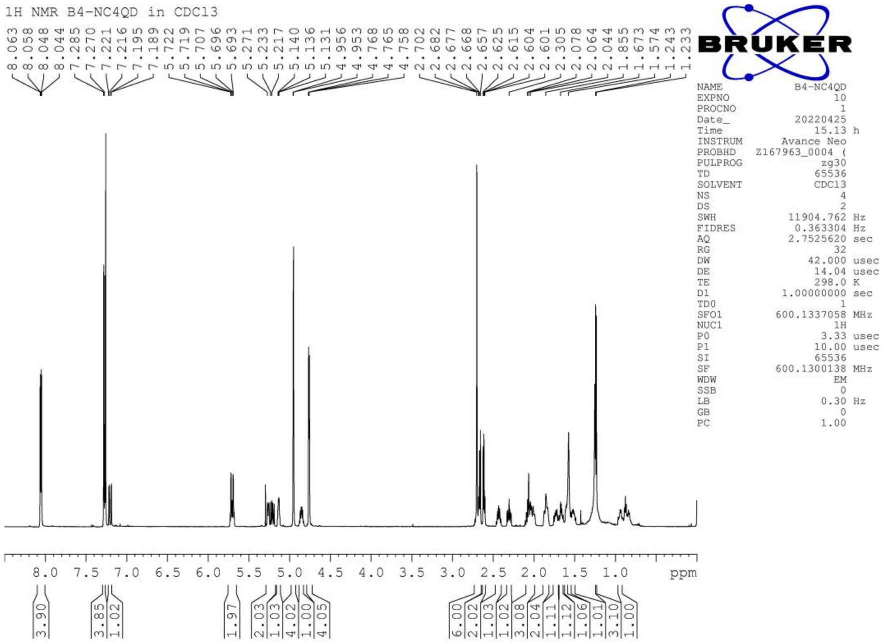

Figure S22. ^{1}H NMR of compound **8c.**

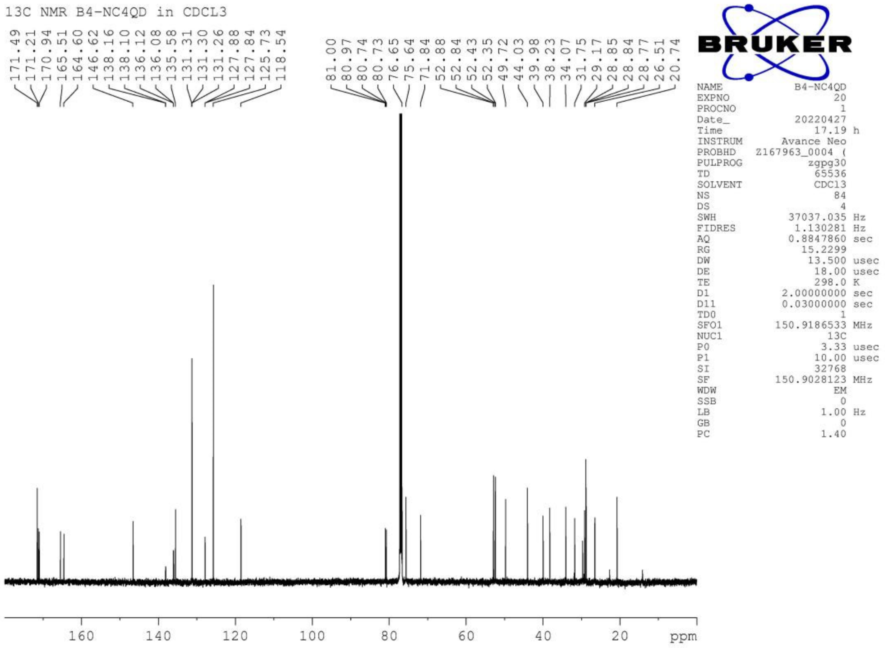

Figure S23. ^{13}C NMR of compound **8c.**

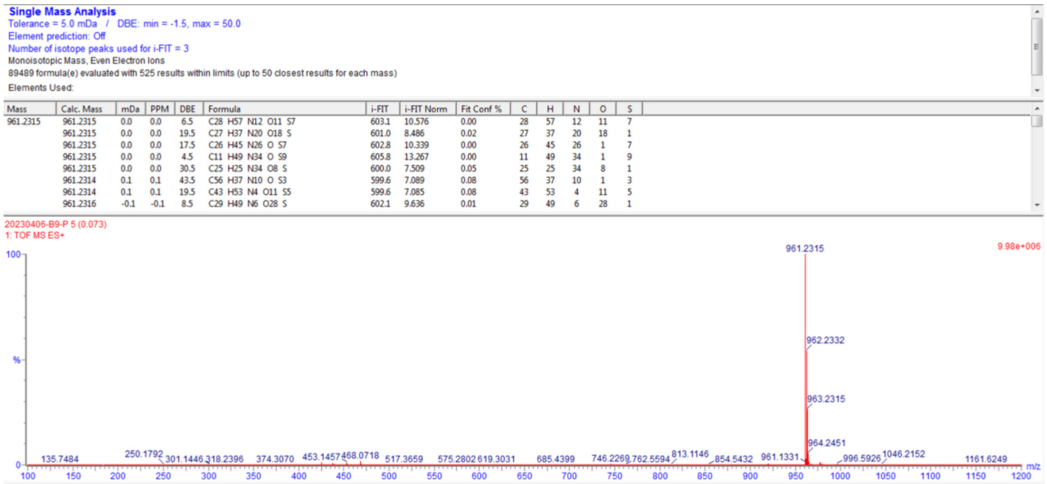

Mass	Calc. Mass	mDa	PPM	DBE	Formula	i-FIT	i-FIT Norm	Fit Conf %	C	H	N	O	S
961.2315	961.2315	0.0	0.0	6.5	C28 H57 N12 O11 S7	603.1	10.576	0.00	28	57	12	11	7
	961.2315	0.0	0.0	19.5	C27 H37 N20 O18 S	601.0	8.486	0.02	27	37	20	18	1
	961.2315	0.0	0.0	17.5	C26 H45 N26 O S7	602.8	10.339	0.00	26	45	26	1	7
	961.2315	0.0	0.0	4.5	C11 H49 N34 O S9	605.8	13.267	0.00	11	49	34	1	9
	961.2315	0.0	0.0	30.5	C25 H25 N34 O8 S	600.0	7.509	0.05	25	25	34	8	1
	961.2314	0.1	0.1	43.5	C56 H37 N10 O S3	599.6	7.089	0.08	56	37	10	1	3
	961.2314	0.1	0.1	19.5	C43 H53 N4 O11 S5	599.6	7.085	0.08	43	53	4	11	5
	961.2316	-0.1	-0.1	8.5	C29 H49 N6 O28 S	602.1	9.636	0.01	29	49	6	28	1

Figure S24. HR-ESIMS of compound **8c.**

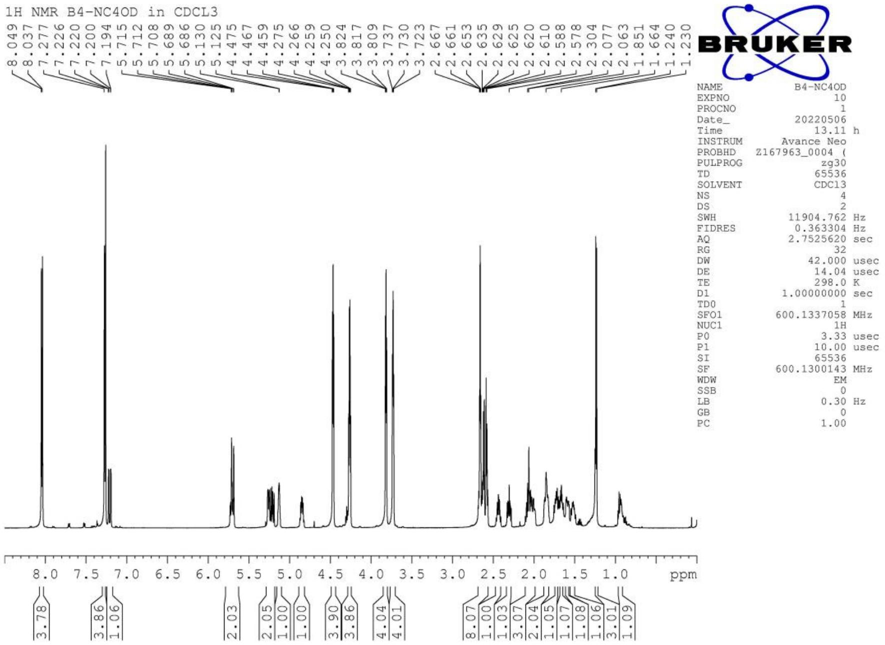

Figure S25. ^{1}H NMR of compound **8d.**

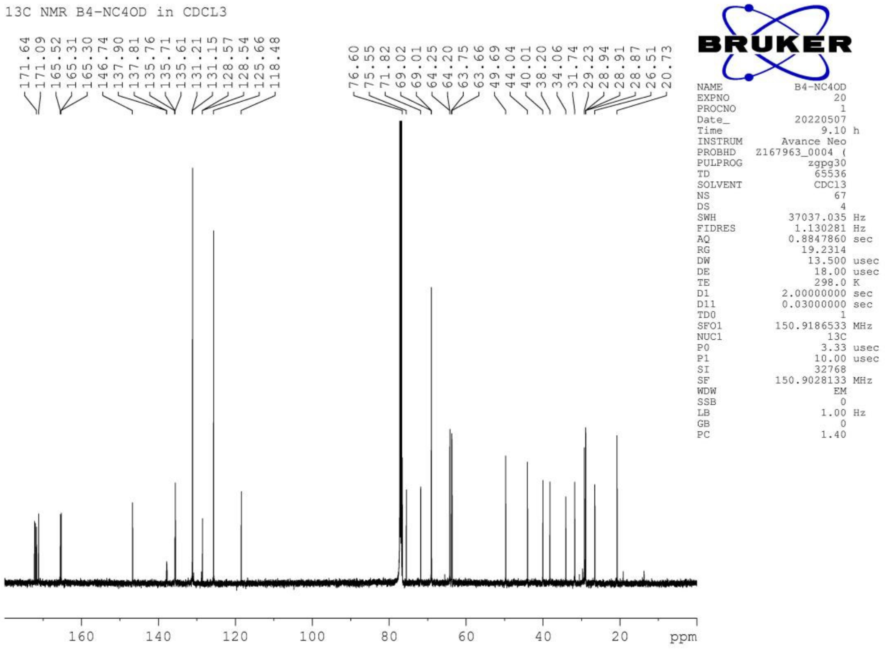

Figure S26. ^{13}C NMR of compound **8d.**

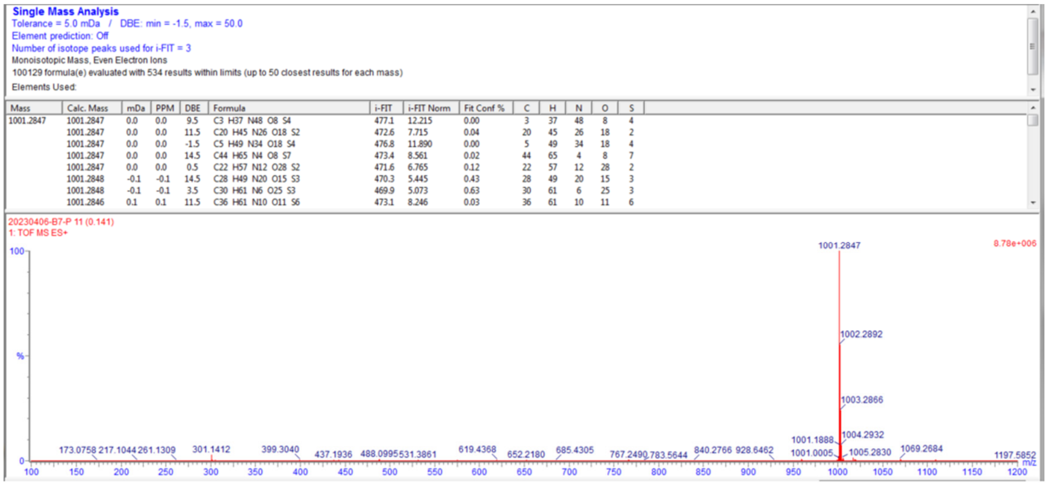

Mass	Calc. Mass	mDa	PPM	DBE	Formula	i-FIT	i-FIT Norm	Fit Conf %	C	H	N	O	S
1001.2847	1001.2847	0.0	0.0	9.5	C3 H37 N48 O8 S4	477.1	12.215	0.00	3	37	48	8	4
	1001.2847	0.0	0.0	11.5	C20 H45 N26 O18 S2	472.6	7.715	0.04	20	45	26	18	2
	1001.2847	0.0	0.0	-1.5	C5 H49 N34 O18 S4	476.8	11.890	0.00	5	49	34	18	4
	1001.2847	0.0	0.0	14.5	C44 H65 N4 O8 S7	473.4	8.561	0.02	44	65	4	8	7
	1001.2847	0.0	0.0	0.5	C22 H57 N12 O28 S2	471.6	6.765	0.12	22	57	12	28	2
	1001.2848	-0.1	-0.1	14.5	C28 H49 N20 O15 S3	470.3	5.445	0.43	28	49	20	15	3
	1001.2848	-0.1	-0.1	3.5	C30 H61 N6 O25 S3	469.9	5.073	0.63	30	61	6	25	3
	1001.2846	0.1	0.1	11.5	C36 H61 N10 O11 S6	473.1	8.246	0.03	36	61	10	11	6

Figure S27. HR-ESIMS of compound **8d.**

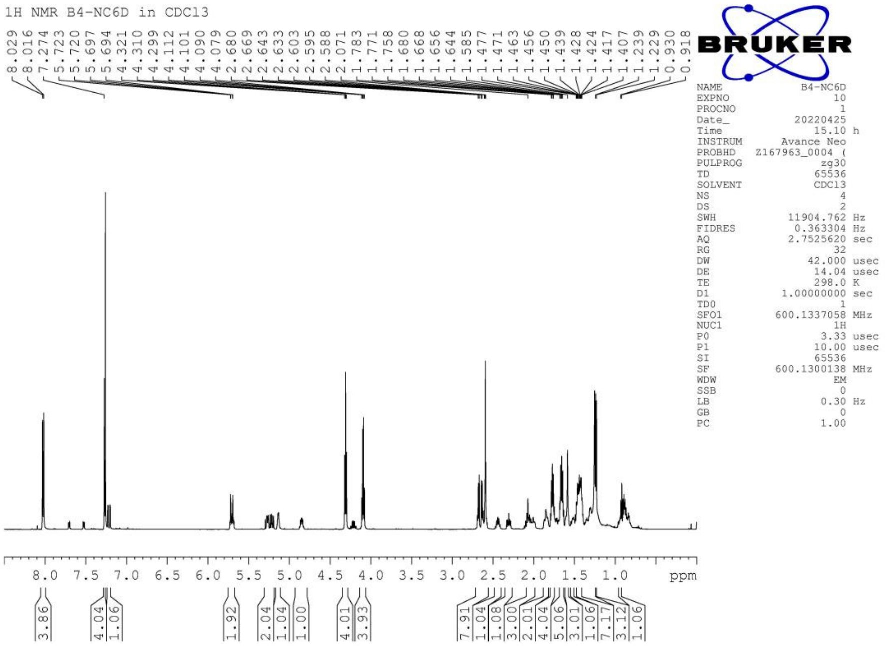

Figure S28. ^{1}H NMR of compound **8e.**

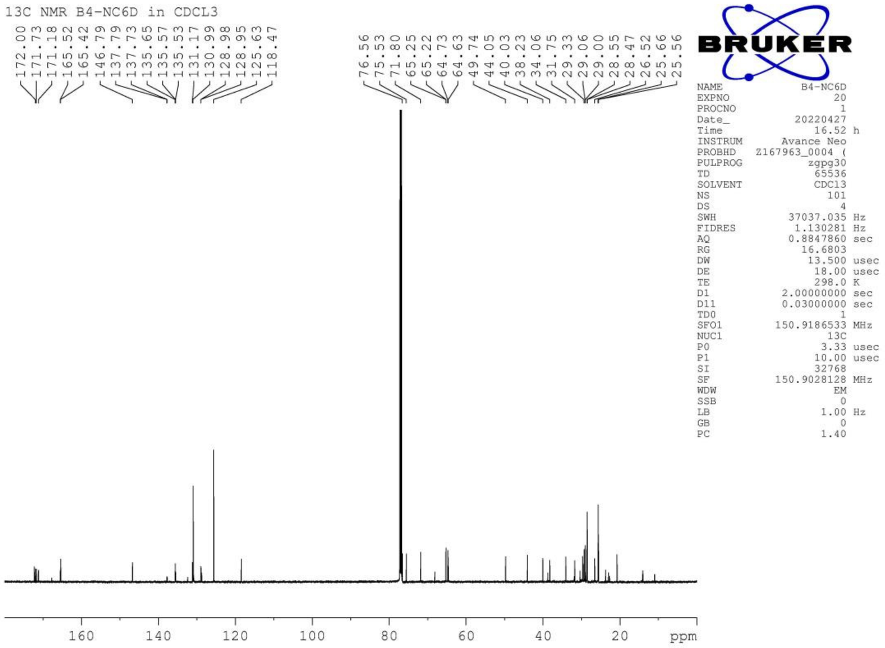

Figure S29. ^{13}C NMR of compound **8e.**

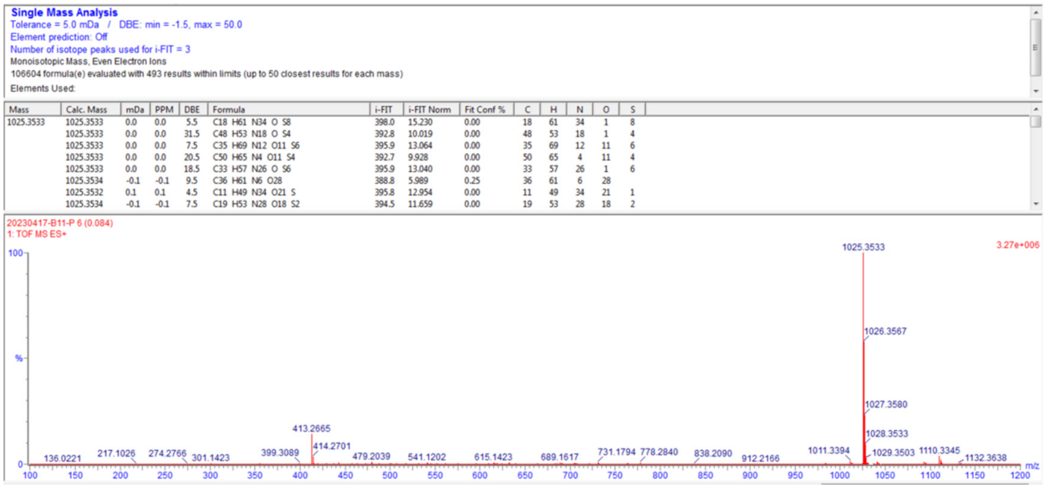

Mass	Calc. Mass	mDa	PPM	DBE	Formula	i-FIT	i-FIT Norm	Fit Conf %	C	H	N	O	S
1025.3533	1025.3533	0.0	0.0	5.5	C18 H61 N34 O S8	398.0	15.230	0.00	18	61	34	1	8
	1025.3533	0.0	0.0	31.5	C48 H53 N18 O S4	392.8	10.019	0.00	48	53	18	1	4
	1025.3533	0.0	0.0	7.5	C35 H69 N12 O11 S6	395.9	13.064	0.00	35	69	12	11	6
	1025.3533	0.0	0.0	20.5	C50 H65 N4 O11 S4	392.7	9.928	0.00	50	65	4	11	4
	1025.3533	0.0	0.0	18.5	C33 H57 N26 O S6	395.9	13.040	0.00	33	57	26	1	6
	1025.3534	-0.1	-0.1	9.5	C36 H61 N6 O28	388.8	5.989	0.25	36	61	6	28	
	1025.3532	0.1	0.1	4.5	C11 H49 N34 O21 S	395.8	12.954	0.00	11	49	34	21	1
	1025.3534	-0.1	-0.1	7.5	C19 H53 N28 O18 S2	394.5	11.659	0.00	19	53	28	18	2

Figure S30. HR-ESIMS of compound **8e.**

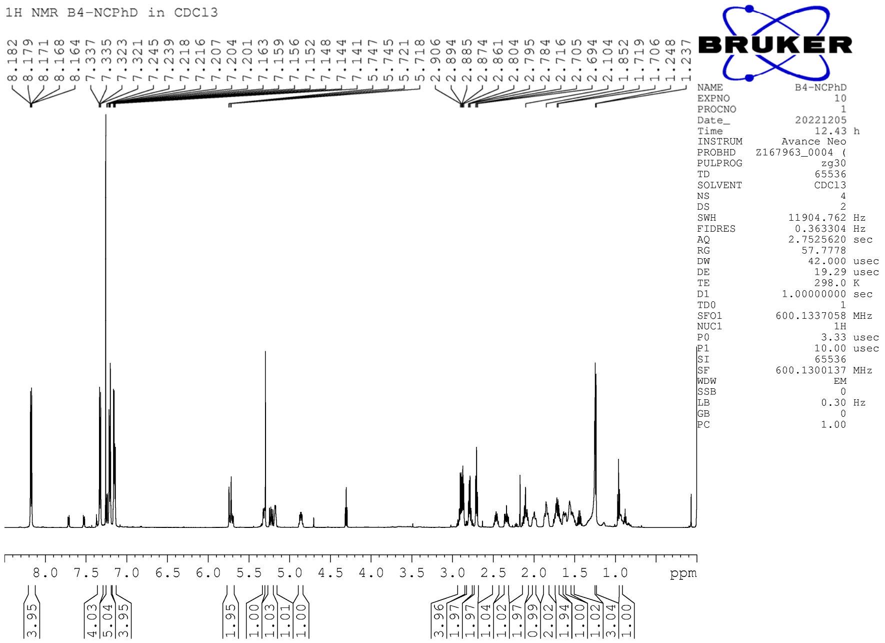

Figure S31. ^{1}H NMR of compound **8f.**

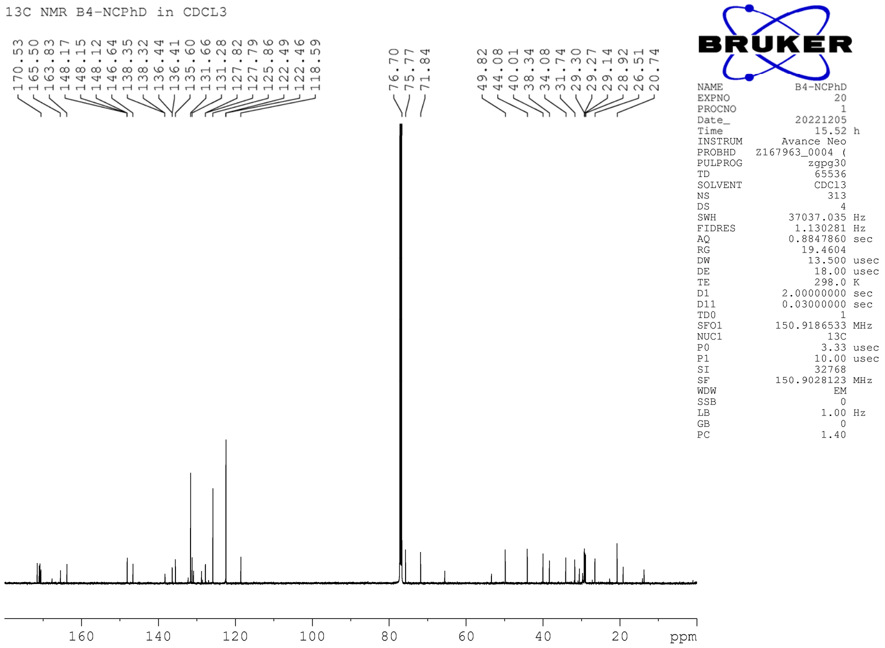

Figure S32. ^{13}C NMR of compound **8f.**

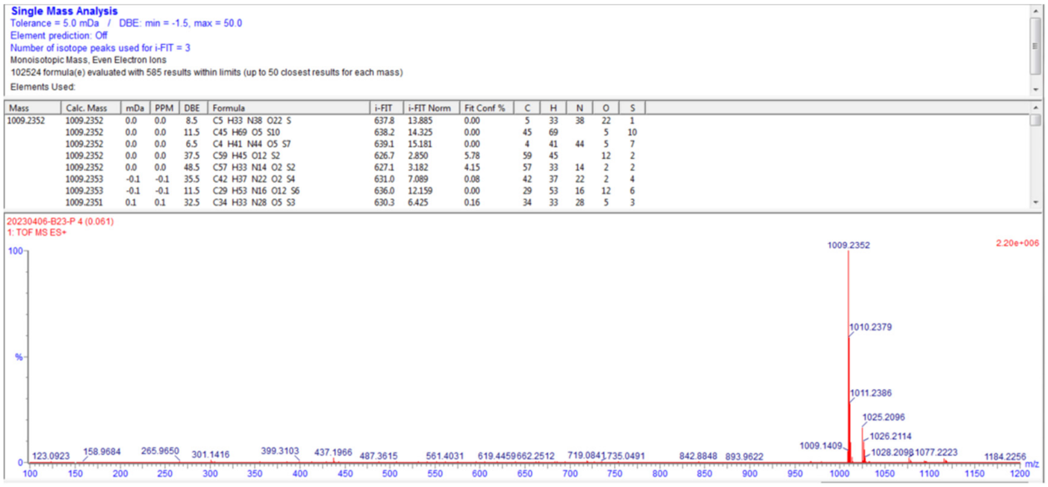

Figure S33. HR-ESIMS of compound **8f.**

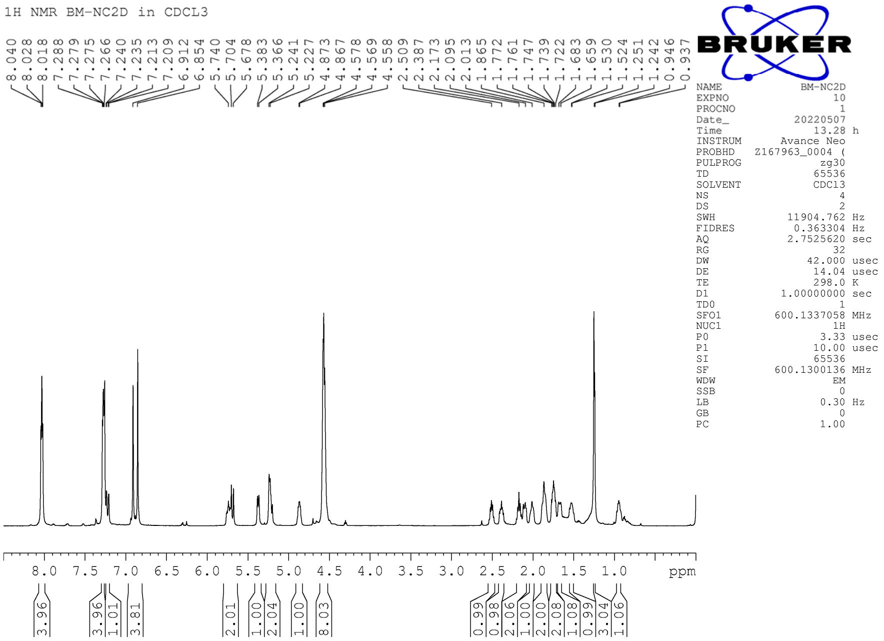

Figure S34. ^{1}H NMR of compound **8g.**

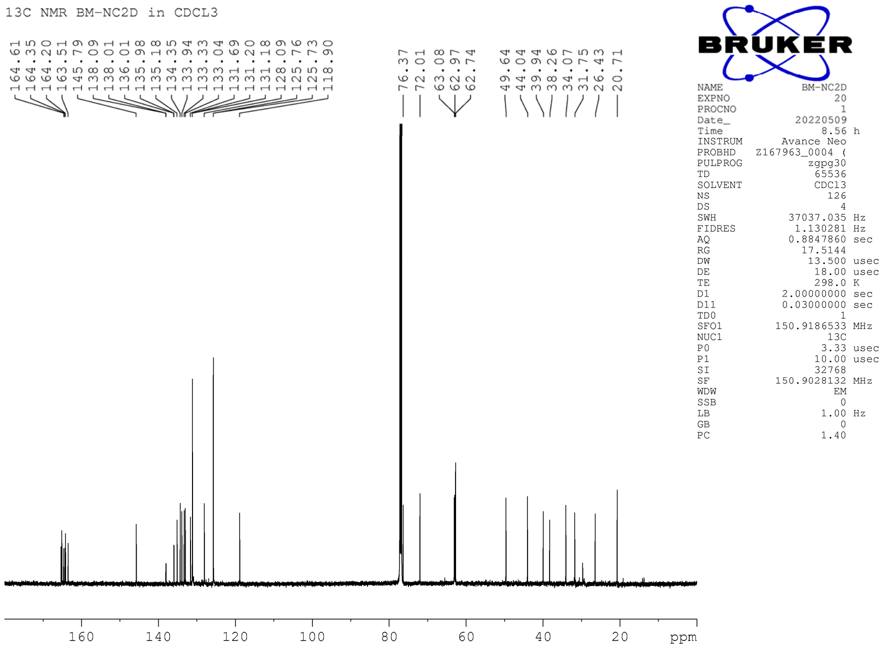

Figure S35. ^{13}C NMR of compound **8g.**

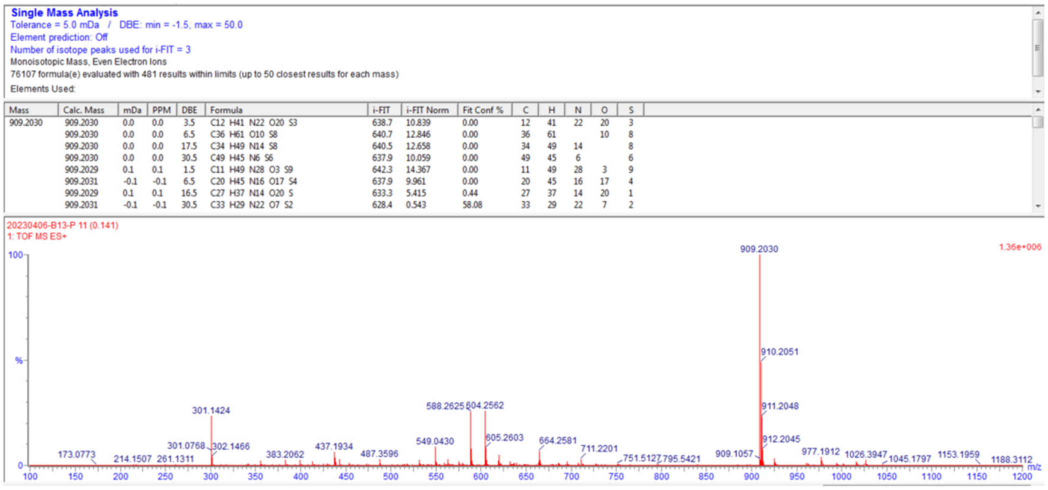

Mass	Calc. Mass	mDa	PPM	DBE	Formula	i-FIT	i-FIT Norm	Fit Conf %	C	H	N	O	S
909.2030	909.2030	0.0	0.0	3.5	C12 H41 N22 O20 S3	638.7	10.839	0.00	12	41	22	20	3
	909.2030	0.0	0.0	6.5	C36 H61 O10 S8	640.7	12.846	0.00	36	61		10	8
	909.2030	0.0	0.0	17.5	C34 H49 N14 S8	640.5	12.658	0.00	34	49	14		8
	909.2030	0.0	0.0	30.5	C49 H45 N6 S6	637.9	10.059	0.00	49	45	6		6
	909.2029	0.1	0.1	1.5	C11 H49 N28 O3 S9	642.3	14.367	0.00	11	49	28	3	9
	909.2031	-0.1	-0.1	6.5	C20 H45 N16 O17 S4	637.9	9.961	0.00	20	45	16	17	4
	909.2029	0.1	0.1	16.5	C27 H37 N14 O20 S	633.3	5.415	0.44	27	37	14	20	1
	909.2031	-0.1	-0.1	30.5	C33 H29 N22 O7 S2	628.4	0.543	58.08	33	29	22	7	2

Figure S36. HR-ESIMS of compound **8g.**

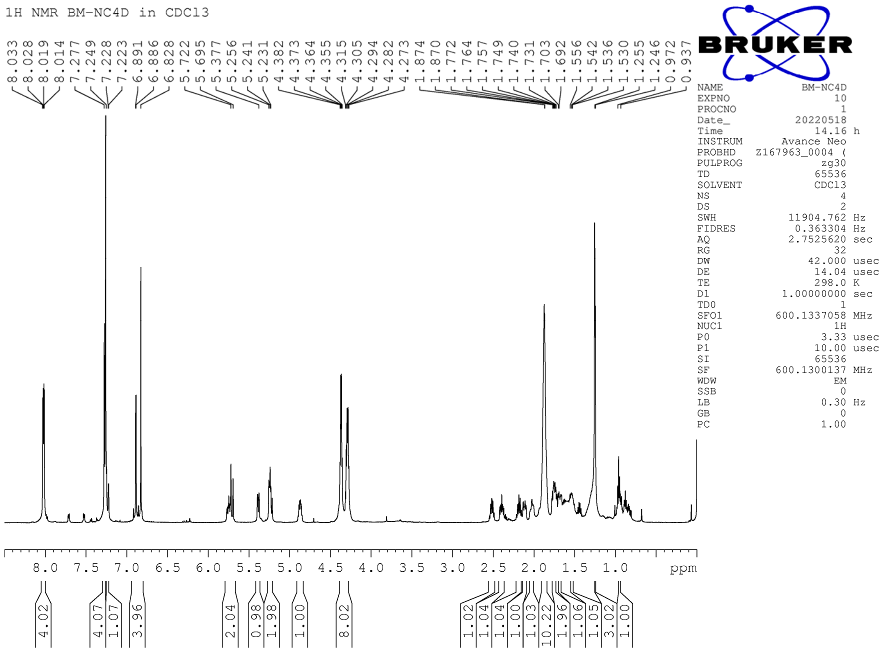

Figure S37. ^{1}H NMR of compound **8h.**

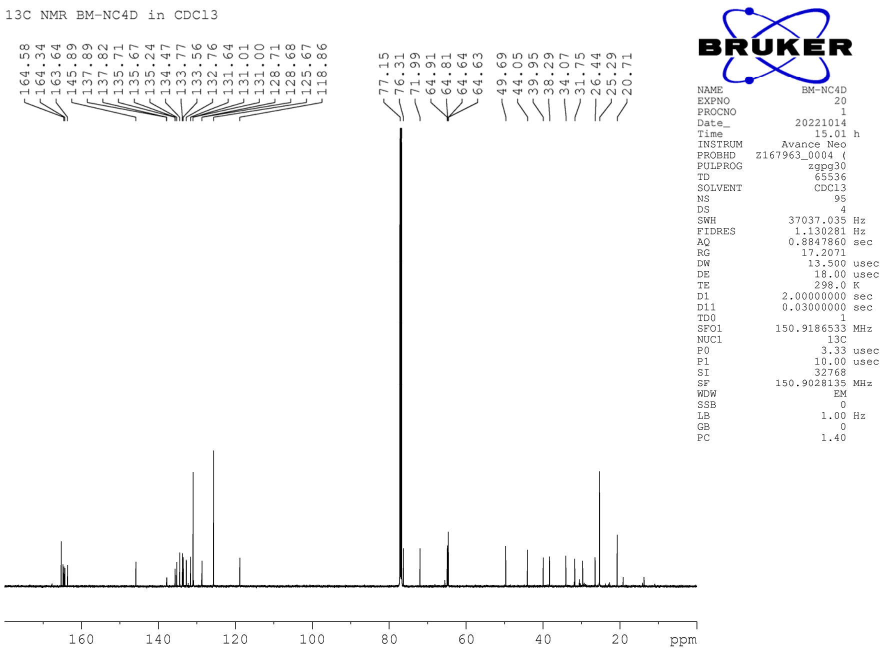

Figure S38. ^{13}C NMR of compound **8h.**

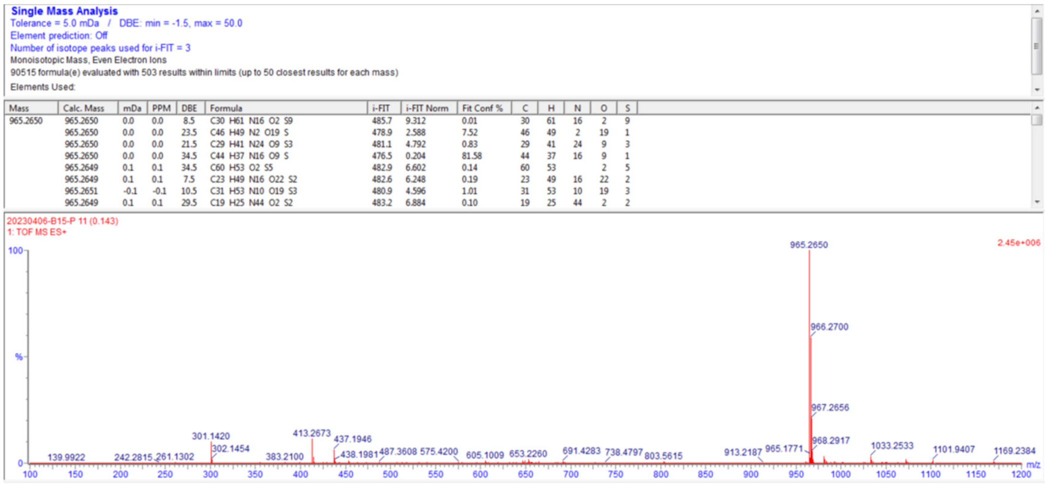

Single Mass Analysis
Tolerance = 5.0 mDa / DBE: min = -1.5, max = 50.0
Element prediction: Off
Number of isotope peaks used for i-FIT = 3
Monoisotopic Mass, Even Electron Ions
90515 formula(e) evaluated with 503 results within limits (up to 50 closest results for each mass)
Elements Used:

Mass	Calc. Mass	mDa	PPM	DBE	Formula	i-FIT	i-FIT Norm	Fit Conf %	C	H	N	O	S
965.2650	965.2650	0.0	0.0	8.5	C30 H61 N16 O2 S9	485.7	9.312	0.01	30	61	16	2	9
	965.2650	0.0	0.0	23.5	C46 H49 N2 O19 S	478.9	2.588	7.52	46	49	2	19	1
	965.2650	0.0	0.0	21.5	C29 H41 N24 O9 S3	481.1	4.792	0.83	29	41	24	9	3
	965.2650	0.0	0.0	34.5	C44 H37 N16 O9 S	476.5	0.204	81.58	44	37	16	9	1
	965.2649	0.1	0.1	34.5	C60 H53 O2 S5	482.9	6.602	0.14	60	53		2	5
	965.2649	0.1	0.1	7.5	C23 H49 N16 O22 S2	482.6	6.248	0.19	23	49	16	22	2
	965.2651	-0.1	-0.1	10.5	C31 H53 N10 O19 S3	480.9	4.596	1.01	31	53	10	19	3
	965.2649	0.1	0.1	29.5	C19 H25 N44 O2 S2	483.2	6.884	0.10	19	25	44	2	2

Figure S39. HR-ESIMS of compound **8h.**

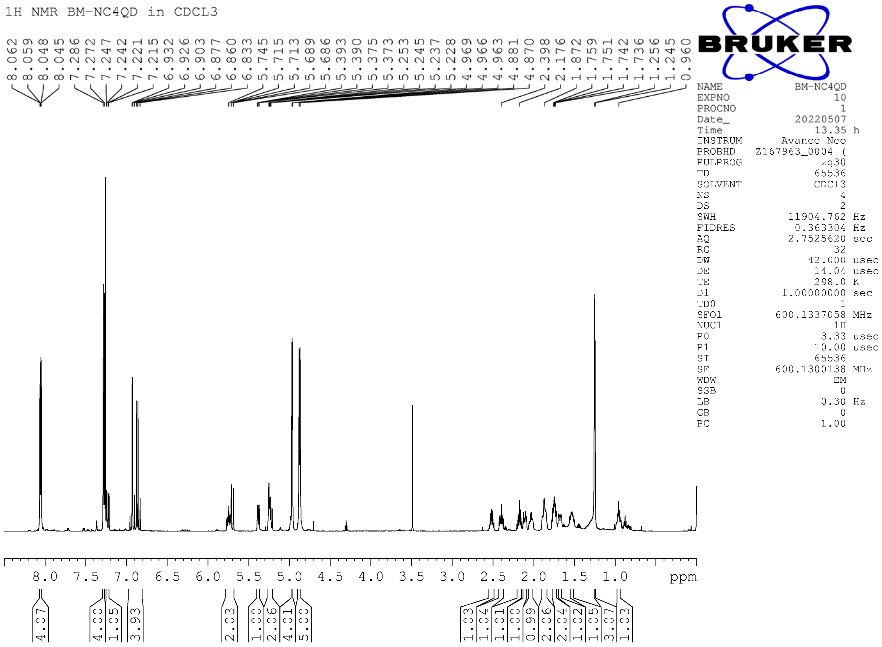

Figure S40. ^{1}H NMR of compound **8i.**

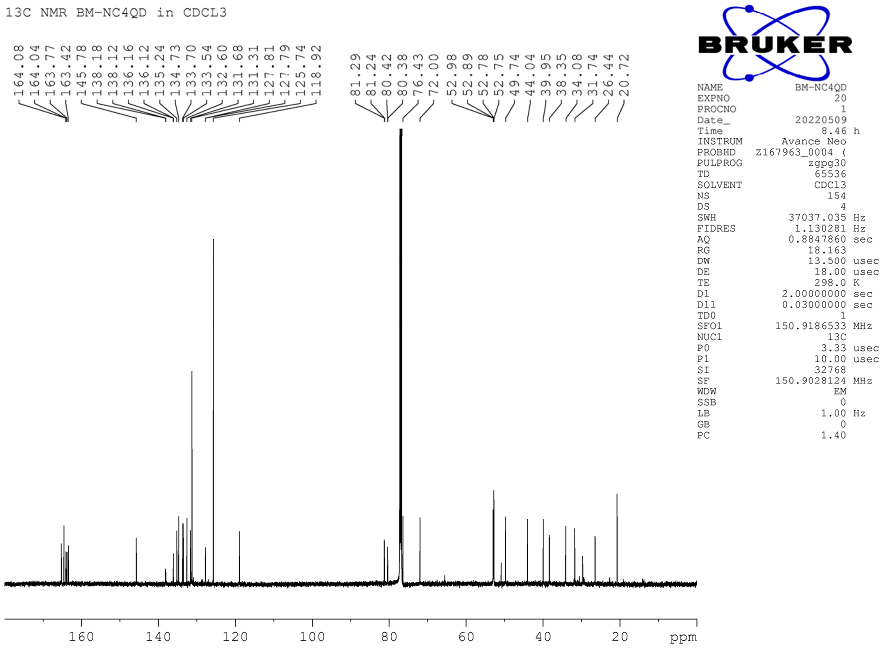

Figure S41. ^{13}C NMR of compound **8i.**

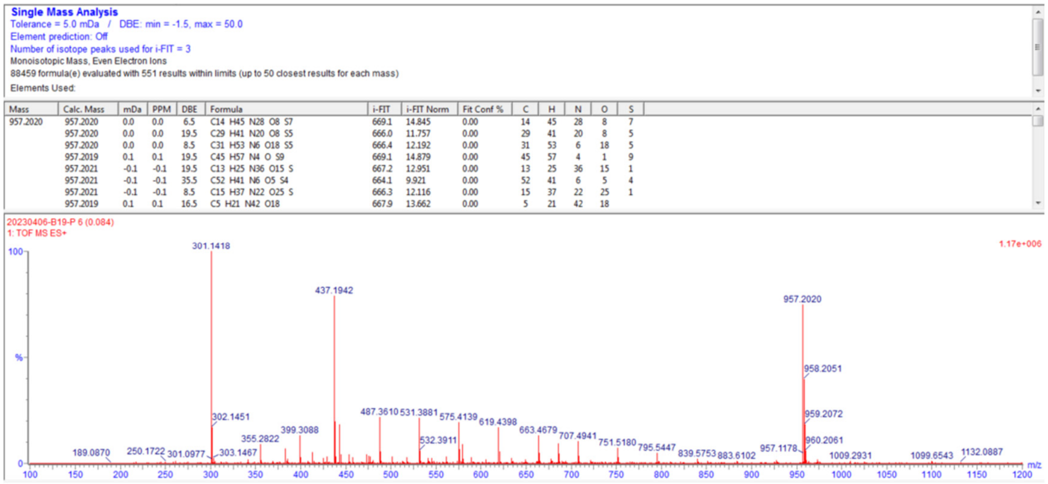

Mass	Calc. Mass	mDa	PPM	DBE	Formula	i-FIT	i-FIT Norm	Fit Conf %	C	H	N	O	S
957.2020	957.2020	0.0	0.0	6.5	C14 H45 N28 O8 S7	669.1	14.845	0.00	14	45	28	8	7
	957.2020	0.0	0.0	19.5	C29 H41 N20 O8 S5	666.0	11.757	0.00	29	41	20	8	5
	957.2020	0.0	0.0	8.5	C31 H53 N6 O18 S5	666.4	12.192	0.00	31	53	6	18	5
	957.2019	0.1	0.1	19.5	C45 H57 N4 O S9	669.1	14.879	0.00	45	57	4	1	9
	957.2021	-0.1	-0.1	19.5	C13 H25 N36 O15 S	667.2	12.951	0.00	13	25	36	15	1
	957.2021	-0.1	-0.1	35.5	C52 H41 N6 O5 S4	664.1	9.921	0.00	52	41	6	5	4
	957.2021	-0.1	-0.1	8.5	C15 H37 N22 O25 S	666.3	12.116	0.00	15	37	22	25	1
	957.2019	0.1	0.1	16.5	C5 H21 N42 O18	667.9	13.662	0.00	5	21	42	18	

Figure S42. HR-ESIMS of compound **8i.**

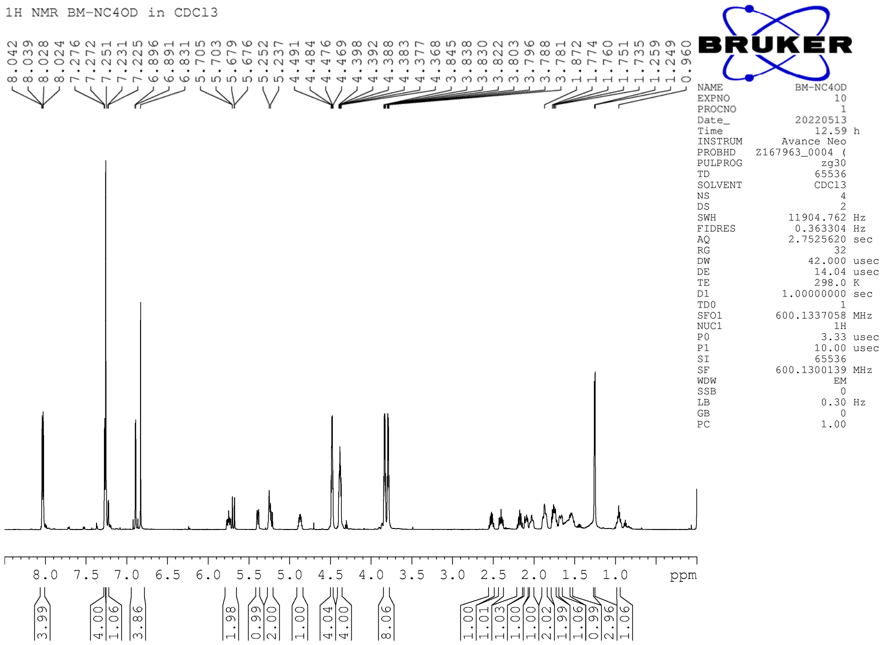

Figure S43. ^{1}H NMR of compound **8j.**

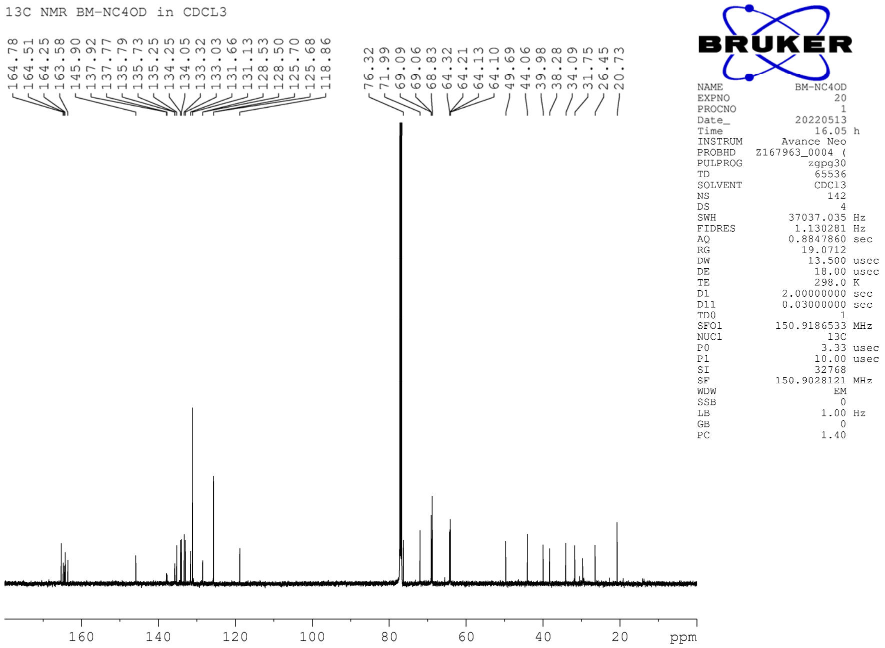

Figure S44. ^{13}C NMR of compound **8j.**

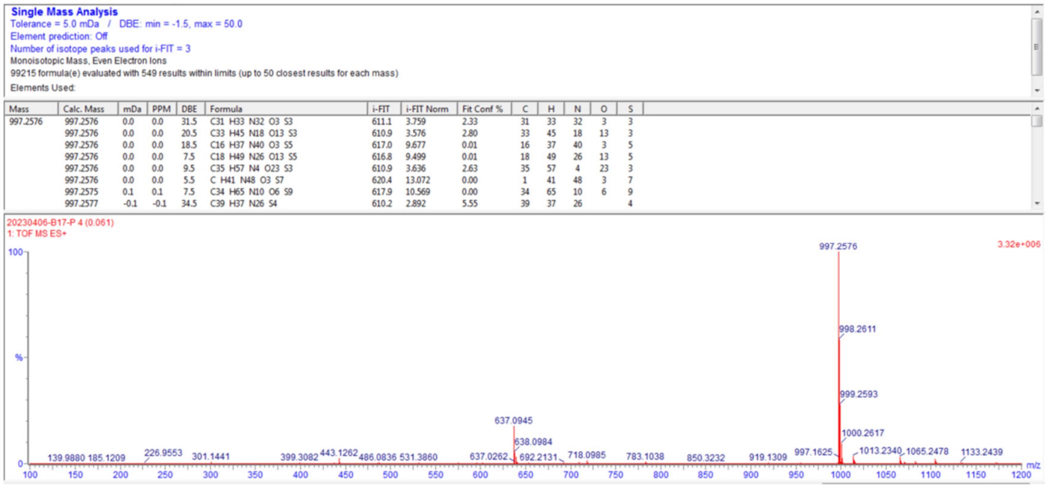

Mass	Calc. Mass	mDa	PPM	DBE	Formula	i-FIT	i-FIT Norm	Fit Conf %	C	H	N	O	S
997.2576	997.2576	0.0	0.0	31.5	C31 H33 N32 O3 S3	611.1	3.759	2.33	31	33	32	3	3
	997.2576	0.0	0.0	20.5	C33 H45 N18 O13 S3	610.9	3.576	2.80	33	45	18	13	3
	997.2576	0.0	0.0	18.5	C16 H37 N40 O3 S5	617.0	9.677	0.01	16	37	40	3	5
	997.2576	0.0	0.0	7.5	C18 H49 N26 O13 S5	616.8	9.499	0.01	18	49	26	13	5
	997.2576	0.0	0.0	9.5	C35 H57 N4 O23 S3	610.9	3.636	2.63	35	57	4	23	3
	997.2576	0.0	0.0	5.5	C H41 N48 O3 S7	620.4	13.072	0.00	1	41	48	3	7
	997.2575	0.1	0.1	7.5	C34 H65 N10 O6 S9	617.9	10.569	0.00	34	65	10	6	9
	997.2577	-0.1	-0.1	34.5	C39 H37 N26 S4	610.2	2.892	5.55	39	37	26		4

Figure S45. HR-ESIMS of compound **8j.**

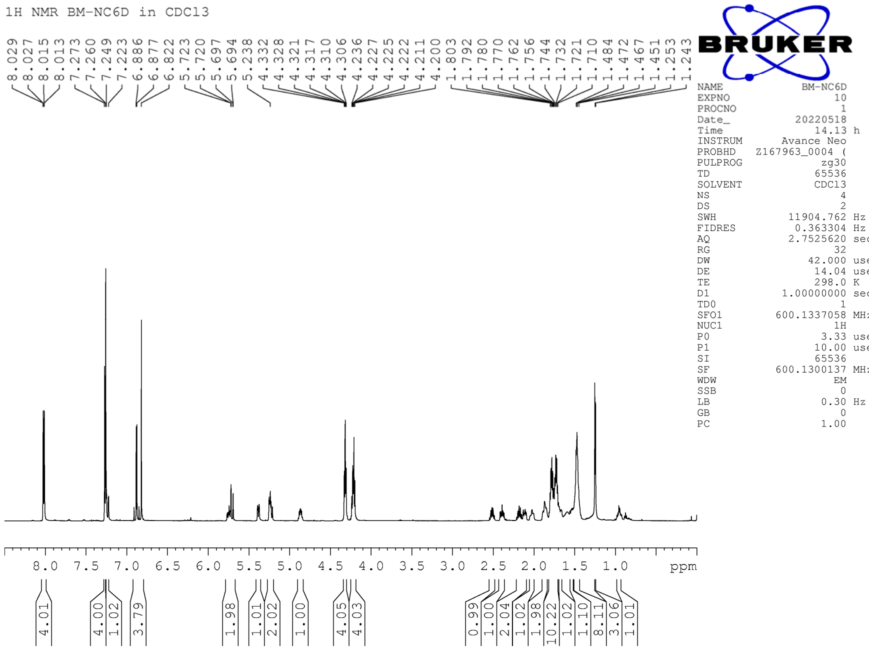

Figure S46. ^{1}H NMR of compound **8k.**

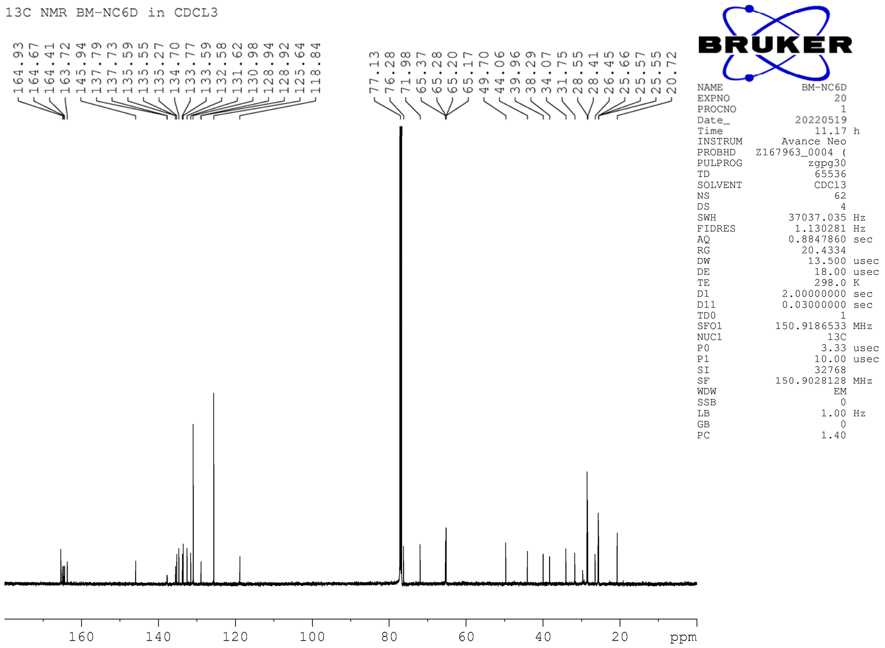

Figure S47. ^{13}C NMR of compound **8k.**

Single Mass Analysis
Tolerance = 5.0 mDa / DBE: min = -1.5, max = 50.0
Element prediction: Off
Number of isotope peaks used for i-FIT = 3
Monoisotopic Mass, Even Electron Ions
105491 formula(e) evaluated with 514 results within limits (up to 50 closest results for each mass)
Elements Used:

Mass	Calc. Mass	mDa	PPM	DBE	Formula	i-FIT	i-FIT Norm	Fit Conf %	C	H	N	O	S
1021.3292	1021.3292	0.0	0.0	29.5	C42 H49 N22 O2 S4	556.5	6.372	0.17	42	49	22	2	4
	1021.3292	0.0	0.0	18.5	C44 H61 N8 O12 S4	556.7	6.573	0.14	44	61	8	12	4
	1021.3292	0.0	0.0	3.5	C12 H57 N38 O2 S8	565.0	14.935	0.00	12	57	38	2	8
	1021.3292	0.0	0.0	16.5	C27 H53 N30 O2 S6	562.0	11.845	0.00	27	53	30	2	6
	1021.3292	0.0	0.0	5.5	C29 H65 N16 O12 S6	562.1	12.009	0.00	29	65	16	12	6
	1021.3291	0.1	0.1	2.5	C5 H45 N38 O22 S	564.7	14.599	0.00	5	45	38	22	1
	1021.3293	-0.1	-0.1	7.5	C30 H57 N10 O29	561.0	10.894	0.00	30	57	10	29	
	1021.3291	0.1	0.1	5.5	C45 H81 O5 S10	564.3	14.230	0.00	45	81		5	10

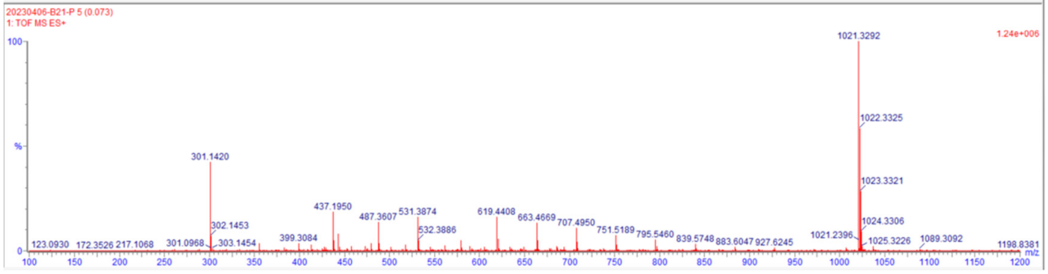

Figure S48. HR-ESIMS of compound **8k.**

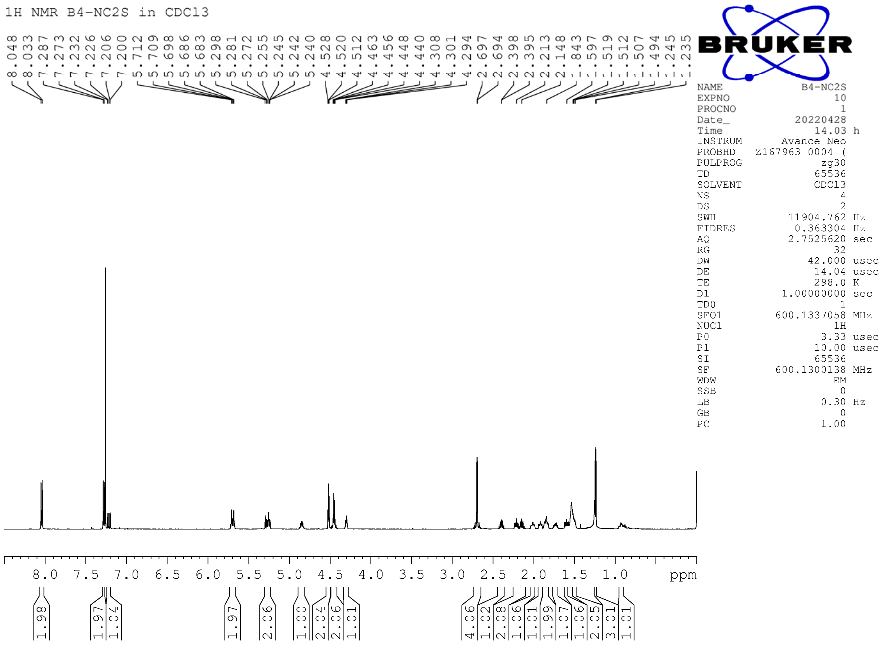

Figure S49. ^{1}H NMR of compound **9a.**

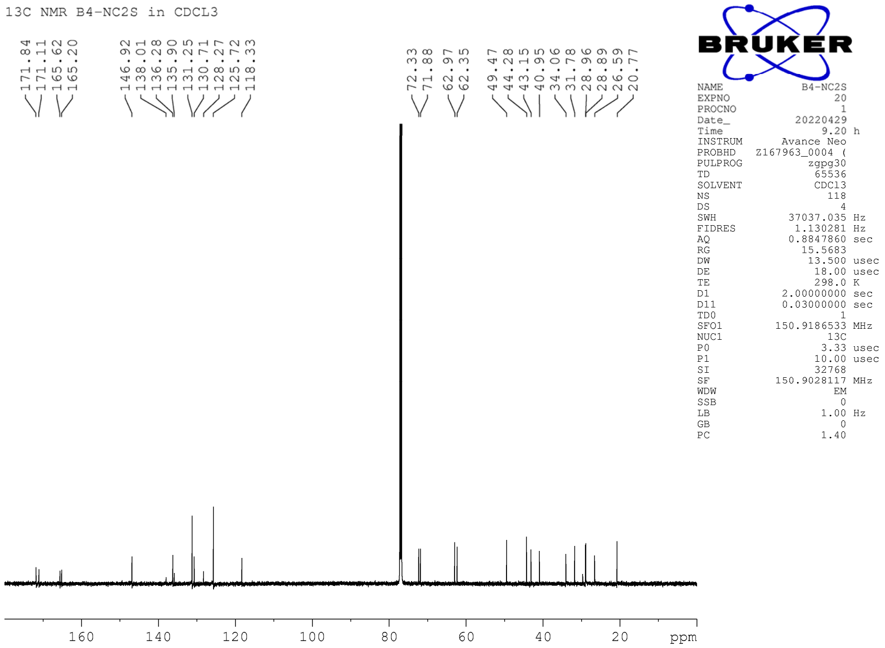

Figure S50. ^{13}C NMR of compound **9a.**

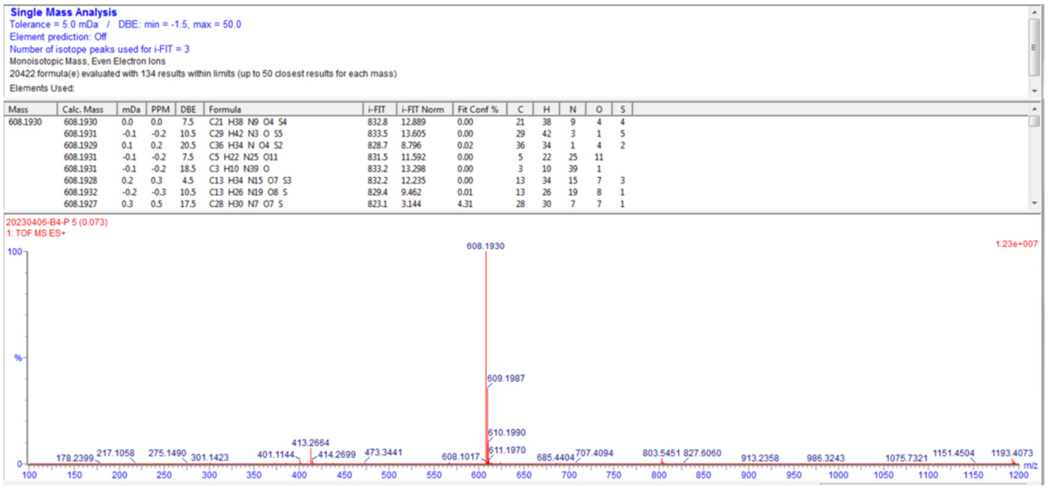

Figure S51. HR-ESIMS of compound **9a.**

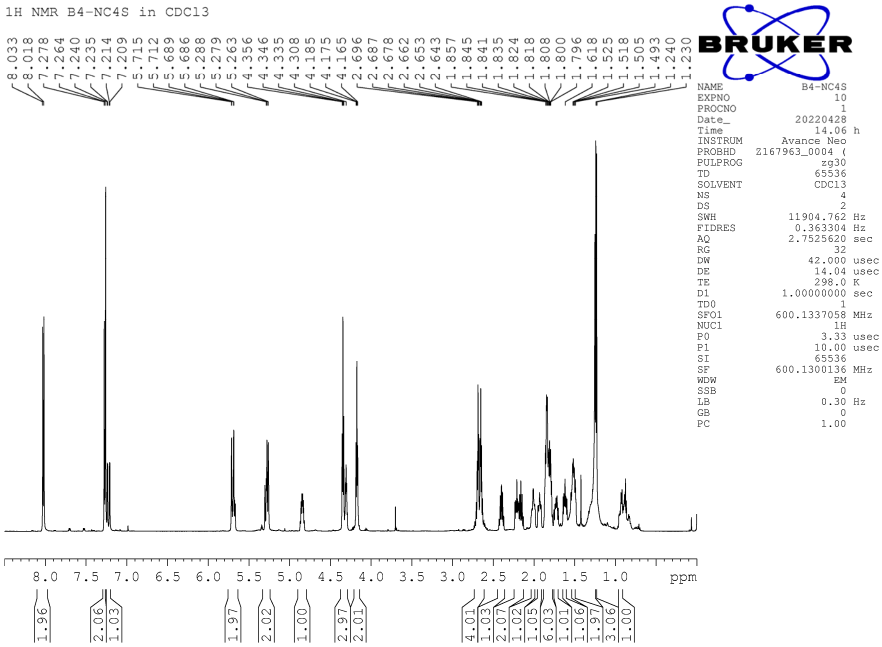

Figure S52. ^{1}H NMR of compound **9b.**

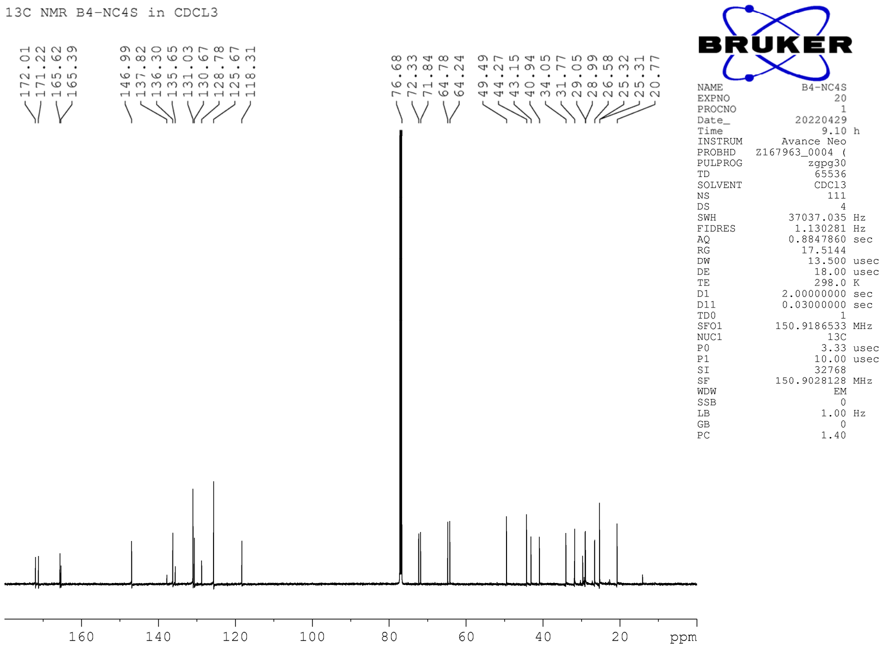

Figure S53. ^{13}C NMR of compound **9b.**

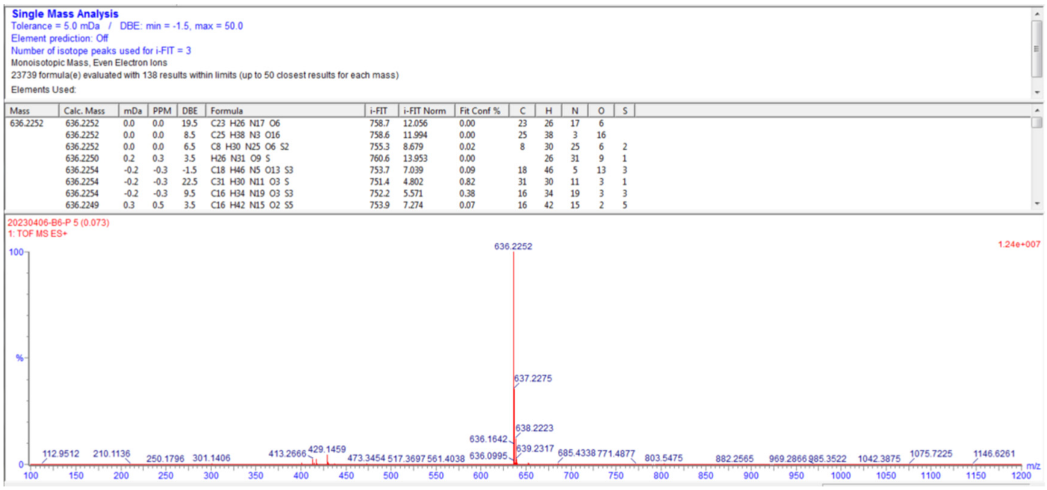

Mass	Calc. Mass	mDa	PPM	DBE	Formula	i-FIT	i-FIT Norm	Fit Conf %	C	H	N	O	S
636.2252	636.2252	0.0	0.0	19.5	C23 H26 N17 O6	758.7	12.056	0.00	23	26	17	6	
	636.2252	0.0	0.0	8.5	C25 H38 N3 O16	758.6	11.994	0.00	25	38	3	16	
	636.2252	0.0	0.0	6.5	C8 H30 N25 O6 S2	755.3	8.679	0.02	8	30	25	6	2
	636.2250	0.2	0.3	3.5	H26 N31 O9 S	760.6	13.953	0.00		26	31	9	1
	636.2254	-0.2	-0.3	-1.5	C18 H46 N5 O13 S3	753.7	7.039	0.09	18	46	5	13	3
	636.2254	-0.2	-0.3	22.5	C31 H30 N11 O3 S	751.4	4.802	0.82	31	30	11	3	1
	636.2254	-0.2	-0.3	9.5	C16 H34 N19 O3 S3	752.2	5.571	0.38	16	34	19	3	3
	636.2249	0.3	0.5	3.5	C16 H42 N15 O2 S5	753.9	7.274	0.07	16	42	15	2	5

Figure S54. HR-ESIMS of compound **9b.**

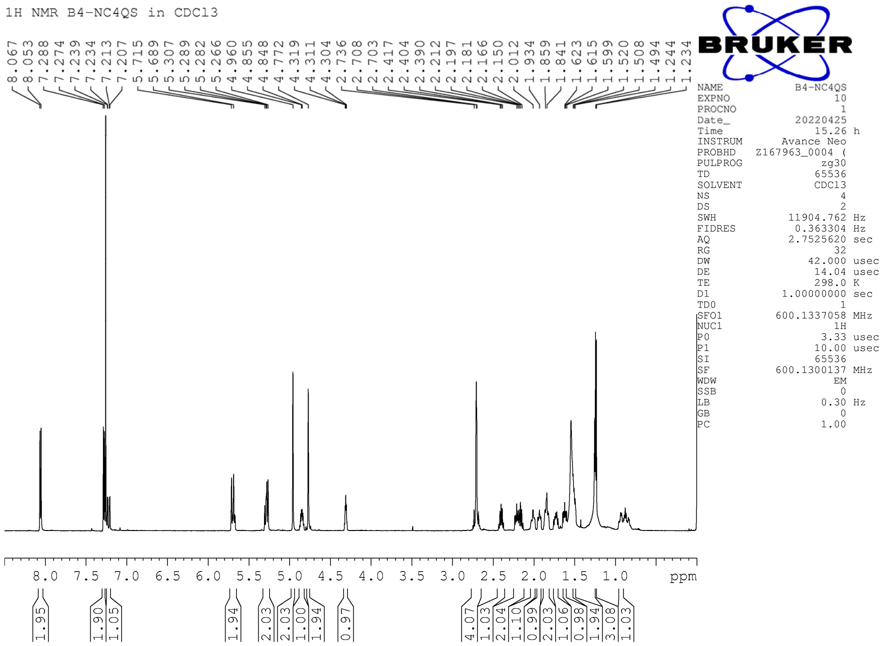

Figure S55. ^{1}H NMR of compound **9c.**

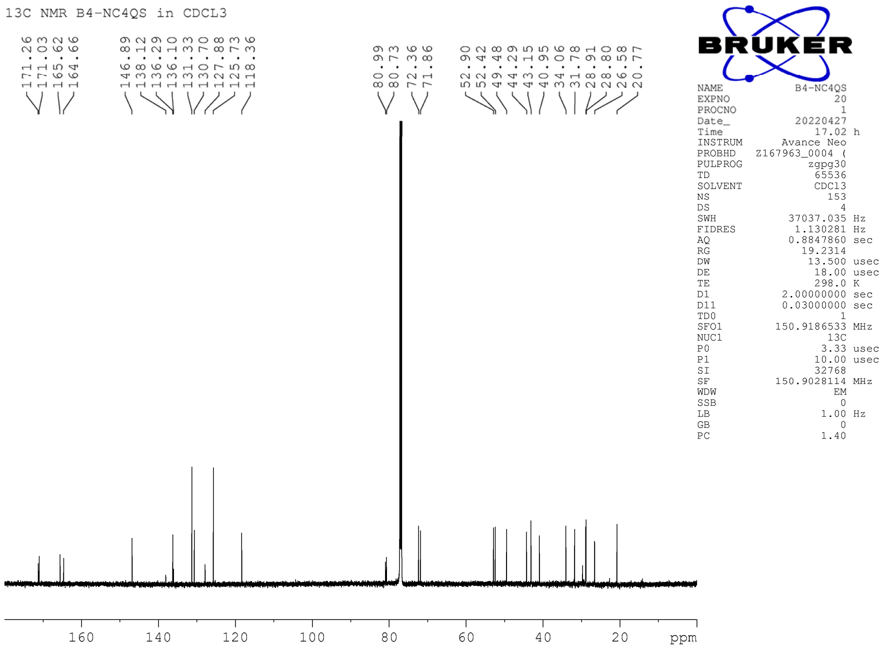

Figure S56. ^{13}C NMR of compound **9c.**

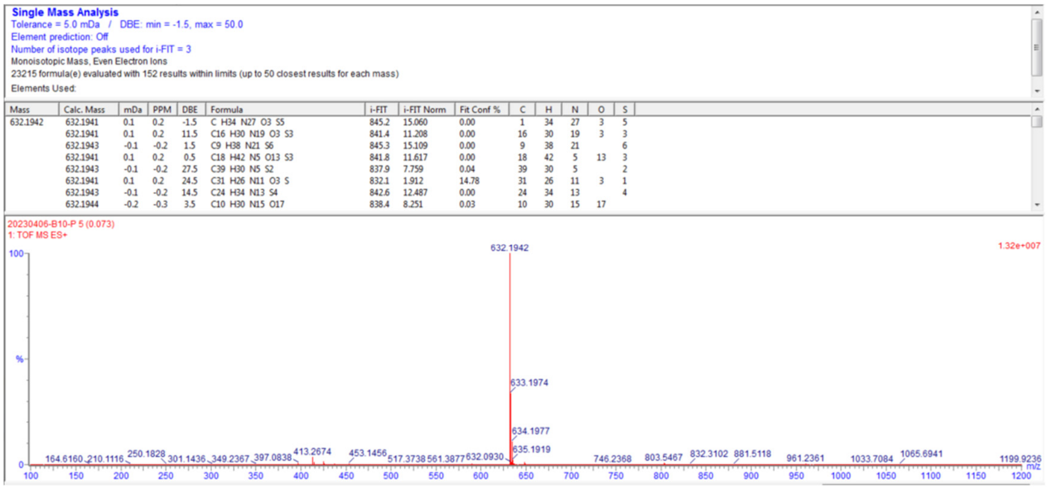

Single Mass Analysis
Tolerance = 5.0 mDa / DBE: min = -1.5, max = 50.0
Element prediction: Off
Number of isotope peaks used for i-FIT = 3
Monoisotopic Mass, Even Electron Ions
23215 formula(e) evaluated with 152 results within limits (up to 50 closest results for each mass)
Elements Used:

Mass	Calc. Mass	mDa	PPM	DBE	Formula	i-FIT	i-FIT Norm	Fit Conf %	C	H	N	O	S
632.1942	632.1941	0.1	0.2	-1.5	C H34 N27 O3 S5	845.2	15.060	0.00	1	34	27	3	5
	632.1941	0.1	0.2	11.5	C16 H30 N19 O3 S3	841.4	11.208	0.00	16	30	19	3	3
	632.1943	-0.1	-0.2	1.5	C9 H38 N21 S6	845.3	15.109	0.00	9	38	21		6
	632.1941	0.1	0.2	0.5	C18 H42 N5 O13 S3	841.8	11.617	0.00	18	42	5	13	3
	632.1943	-0.1	-0.2	27.5	C39 H30 N5 S2	837.9	7.759	0.04	39	30	5		2
	632.1941	0.1	0.2	24.5	C31 H26 N11 O3 S	832.1	1.912	14.78	31	26	11	3	1
	632.1943	-0.1	-0.2	14.5	C24 H34 N13 S4	842.6	12.487	0.00	24	34	13		4
	632.1944	-0.2	-0.3	3.5	C10 H30 N15 O17	838.4	8.251	0.03	10	30	15	17	

Figure S57. HR-ESIMS of compound **9c.**

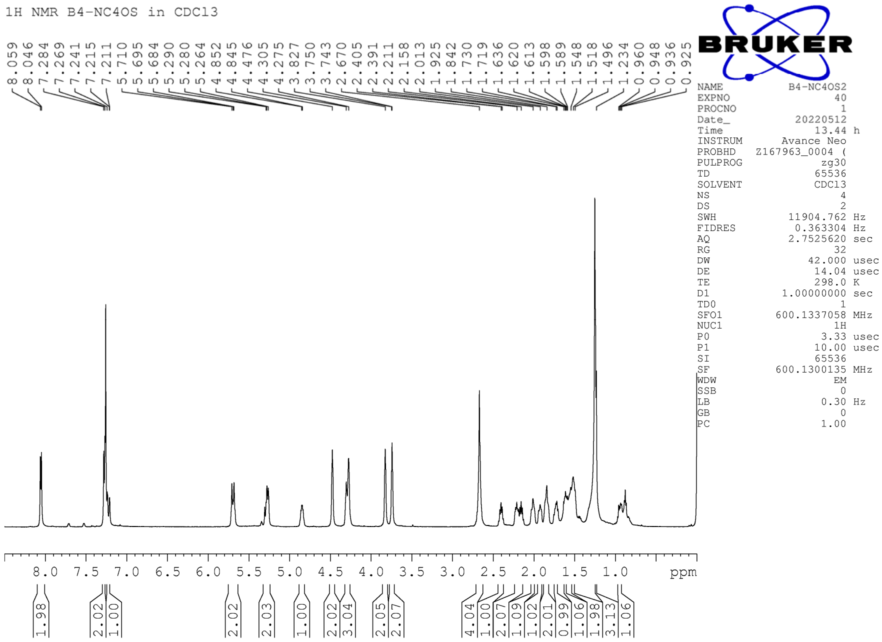

Figure S58. ^{1}H NMR of compound **9d.**

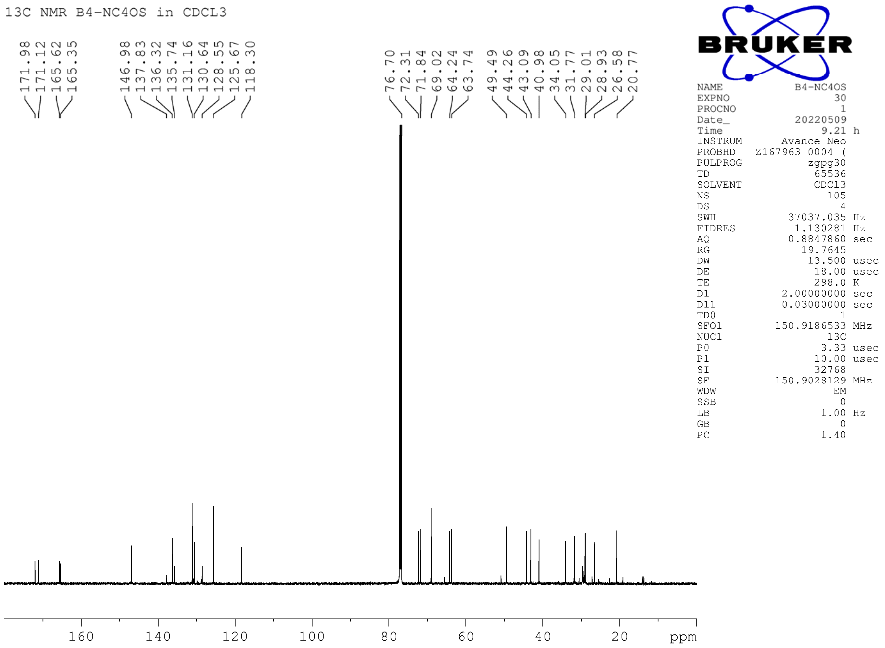

Figure S59. ^{13}C NMR of compound **9d.**

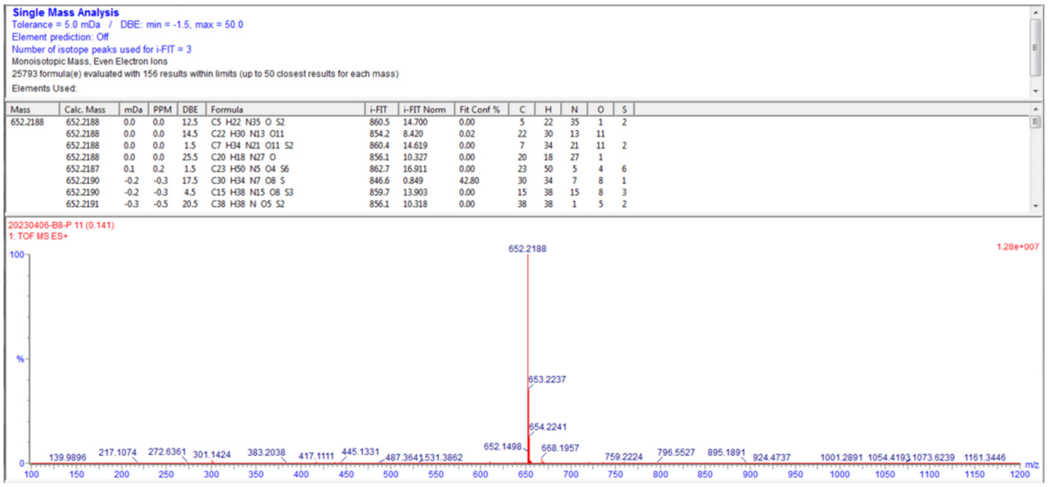

Single Mass Analysis
Tolerance = 5.0 mDa / DBE: min = -1.5, max = 50.0
Element prediction: Off
Number of isotope peaks used for i-FIT = 3
Monoisotopic Mass, Even Electron Ions
25793 formula(e) evaluated with 156 results within limits (up to 50 closest results for each mass)
Elements Used:

Mass	Calc. Mass	mDa	PPM	DBE	Formula	i-FIT	i-FIT Norm	Fit Conf %	C	H	N	O	S
652.2188	652.2188	0.0	0.0	12.5	C5 H22 N35 O S2	860.5	14.700	0.00	5	22	35	1	2
	652.2188	0.0	0.0	14.5	C22 H30 N13 O11	854.2	8.420	0.02	22	30	13	11	
	652.2188	0.0	0.0	1.5	C7 H34 N21 O11 S2	860.4	14.619	0.00	7	34	21	11	2
	652.2188	0.0	0.0	25.5	C20 H18 N27 O	856.1	10.327	0.00	20	18	27	1	
	652.2187	0.1	0.2	1.5	C23 H50 N5 O4 S6	862.7	16.911	0.00	23	50	5	4	6
	652.2190	-0.2	-0.3	17.5	C30 H34 N7 O8 S	846.6	0.849	42.80	30	34	7	8	1
	652.2190	-0.2	-0.3	4.5	C15 H38 N15 O8 S3	859.7	13.903	0.00	15	38	15	8	3
	652.2191	-0.3	-0.5	20.5	C38 H38 N O5 S2	856.1	10.318	0.00	38	38	1	5	2

Figure S60. HR-ESIMS of compound **9d.**

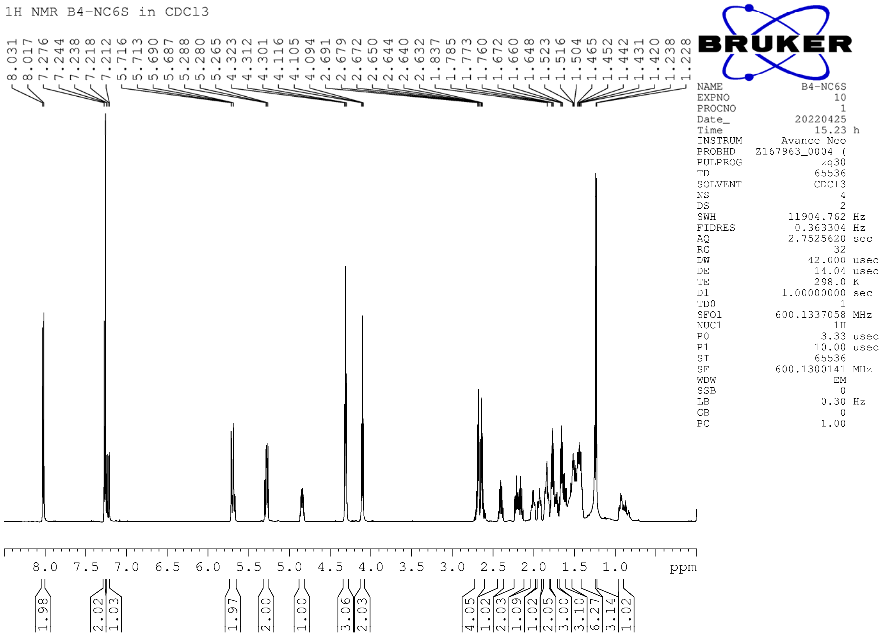

Figure S61. ^{1}H NMR of compound **9e.**

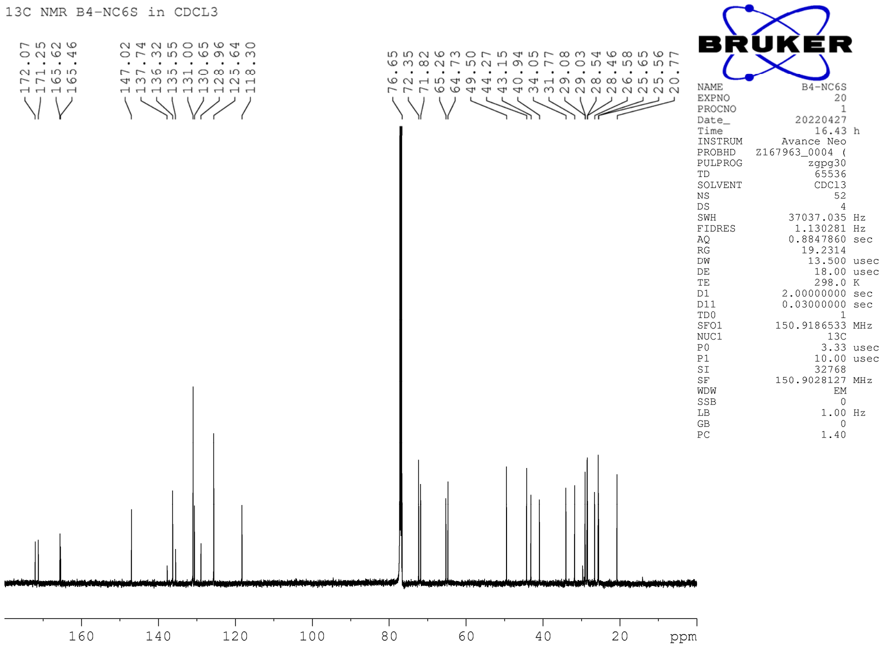

Figure S62. ^{13}C NMR of compound **9e.**

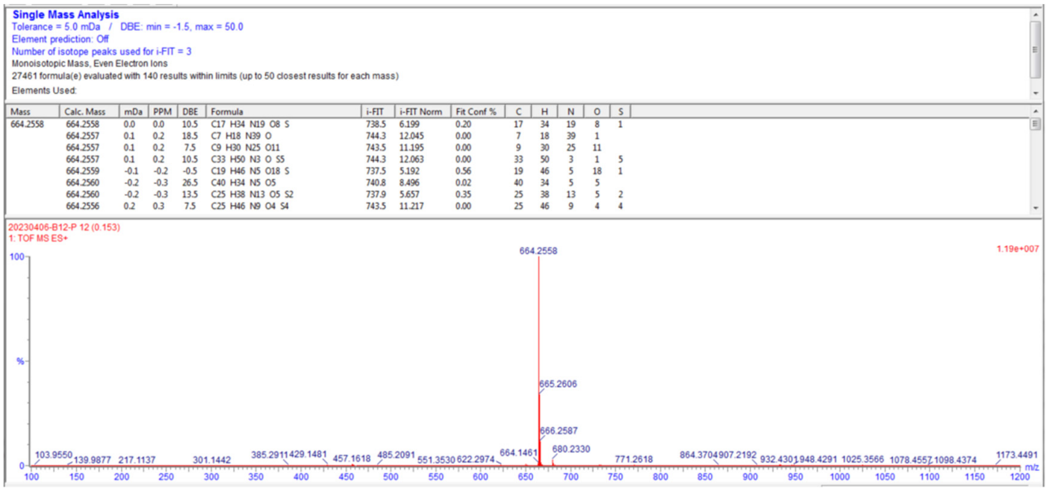

Figure S63. HR-ESIMS of compound **9e.**

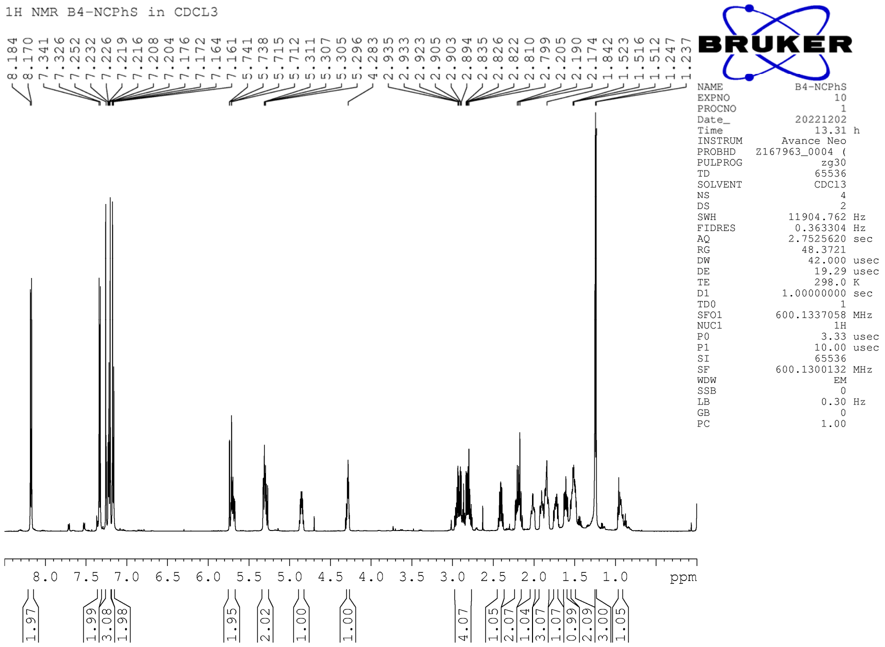

Figure S64. ^{1}H NMR of compound **9f.**

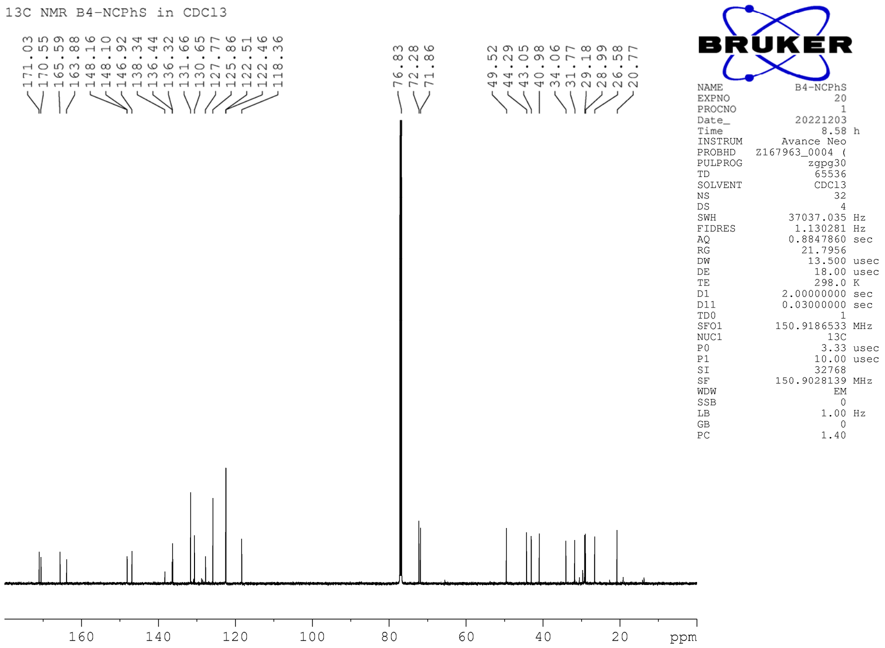

Figure S65. ^{13}C NMR of compound **9f.**

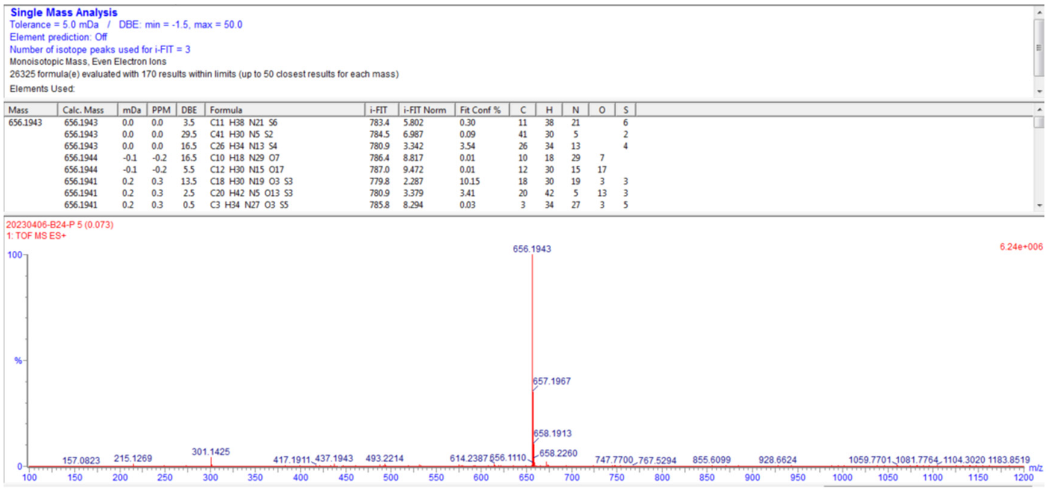

Single Mass Analysis
Tolerance = 5.0 mDa / DBE: min = -1.5, max = 50.0
Element prediction: Off
Number of isotope peaks used for i-FIT = 3
Monoisotopic Mass, Even Electron Ions
26325 formula(e) evaluated with 170 results within limits (up to 50 closest results for each mass)
Elements Used:

Mass	Calc. Mass	mDa	PPM	DBE	Formula	i-FIT	i-FIT Norm	Fit Conf %	C	H	N	O	S
656.1943	656.1943	0.0	0.0	3.5	C11 H38 N21 S6	783.4	5.802	0.30	11	38	21		6
	656.1943	0.0	0.0	29.5	C41 H30 N5 S2	784.5	6.987	0.09	41	30	5		2
	656.1943	0.0	0.0	16.5	C26 H34 N13 S4	780.9	3.342	3.54	26	34	13		4
	656.1944	-0.1	-0.2	16.5	C10 H18 N29 O7	786.4	8.817	0.01	10	18	29	7	
	656.1944	-0.1	-0.2	5.5	C12 H30 N15 O17	787.0	9.472	0.01	12	30	15	17	
	656.1941	0.2	0.3	13.5	C18 H30 N19 O3 S3	779.8	2.287	10.15	18	30	19	3	3
	656.1941	0.2	0.3	2.5	C20 H42 N5 O13 S3	780.9	3.379	3.41	20	42	5	13	3
	656.1941	0.2	0.3	0.5	C3 H34 N27 O3 S5	785.8	8.294	0.03	3	34	27	3	5

Figure S66. HR-ESIMS of compound **9f.**

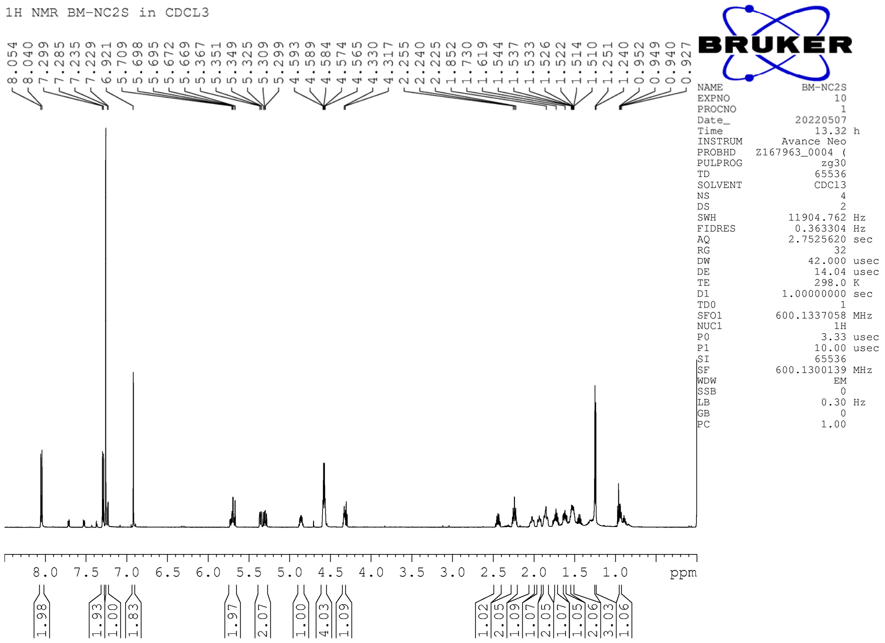

Figure S67. ^{1}H NMR of compound **9g.**

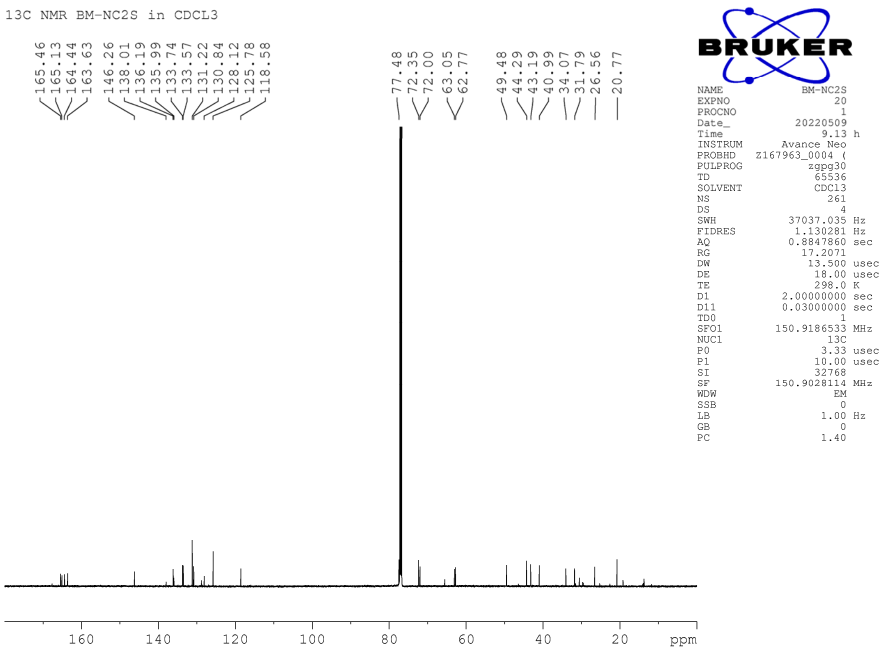

Figure S68. ^{13}C NMR of compound **9g.**

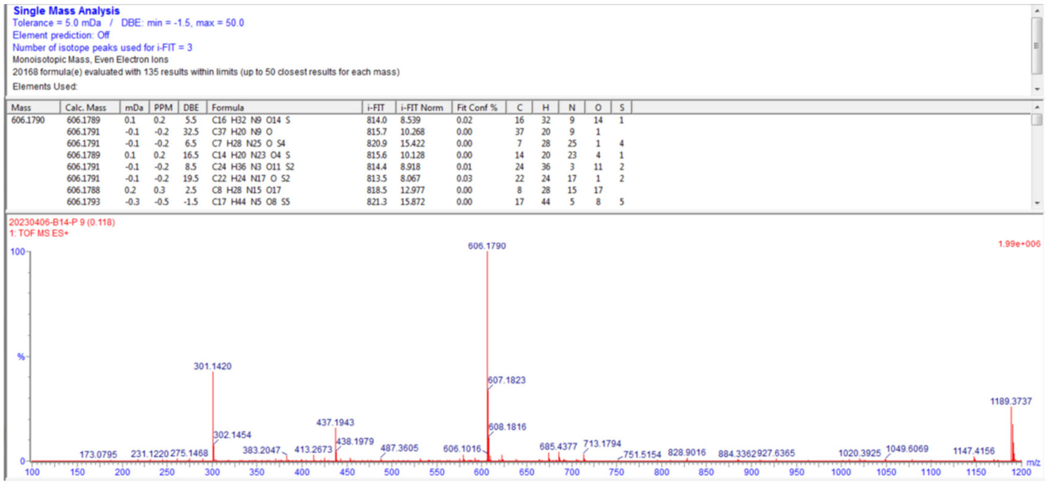

Mass	Calc. Mass	mDa	PPM	DBE	Formula	i-FIT	i-FIT Norm	Fit Conf %	C	H	N	O	S
606.1790	606.1789	0.1	0.2	5.5	C16 H32 N9 O14 S	814.0	8.539	0.02	16	32	9	14	1
	606.1791	-0.1	-0.2	32.5	C37 H20 N9 O	815.7	10.268	0.00	37	20	9	1	
	606.1791	-0.1	-0.2	6.5	C7 H28 N25 O S4	820.9	15.422	0.00	7	28	25	1	4
	606.1789	0.1	0.2	16.5	C14 H20 N23 O4 S	815.6	10.128	0.00	14	20	23	4	1
	606.1791	-0.1	-0.2	8.5	C24 H36 N3 O11 S2	814.4	8.918	0.01	24	36	3	11	2
	606.1791	-0.1	-0.2	19.5	C22 H24 N17 O S2	813.5	8.067	0.03	22	24	17	1	2
	606.1788	0.2	0.3	2.5	C8 H28 N15 O17	818.5	12.977	0.00	8	28	15	17	
	606.1793	-0.3	-0.5	-1.5	C17 H44 N5 O8 S5	821.3	15.872	0.00	17	44	5	8	5

Figure S69. HR-ESIMS of compound **9g.**

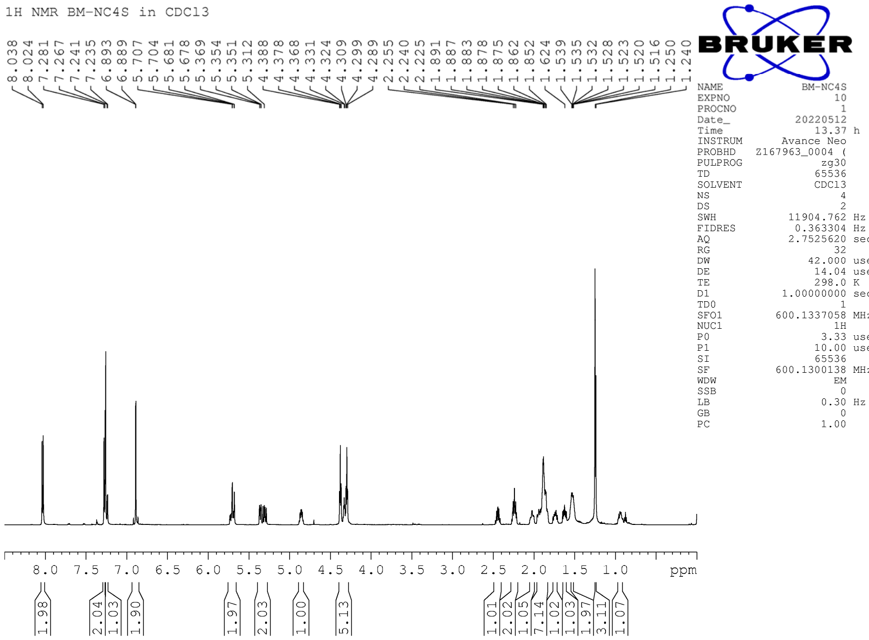

Figure S70. ^{1}H NMR of compound **9h.**

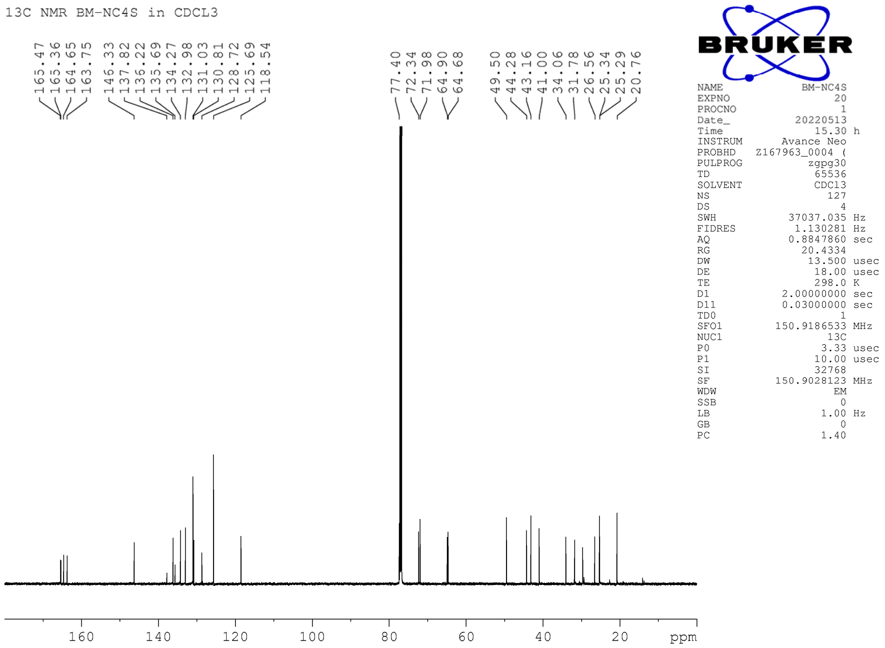

Figure S71. ^{13}C NMR of compound **9h.**

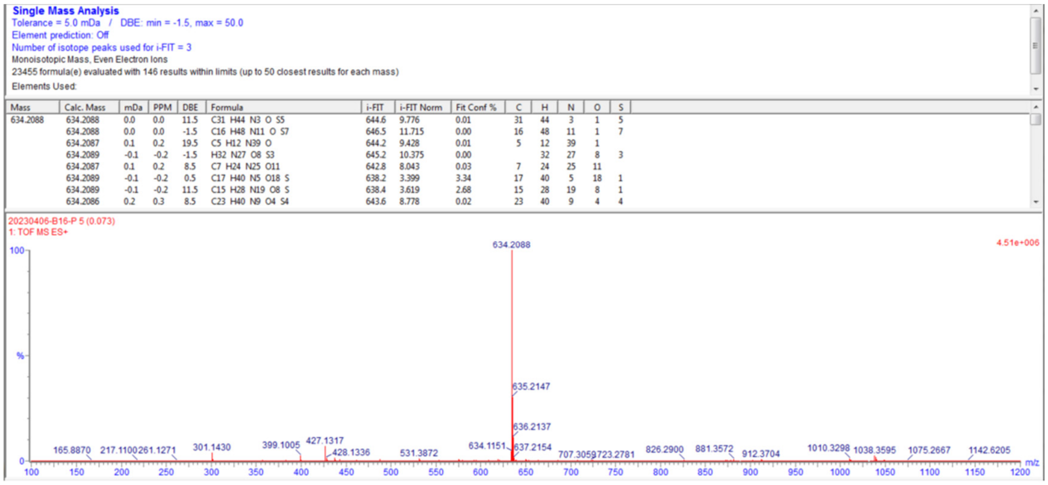
Single Mass Analysis
Tolerance = 5.0 mDa / DBE: min = -1.5, max = 50.0
Element prediction: Off
Number of isotope peaks used for i-FIT = 3
Monoisotopic Mass, Even Electron Ions
23455 formula(e) evaluated with 146 results within limits (up to 50 closest results for each mass)
Elements Used:

Mass	Calc. Mass	mDa	PPM	DBE	Formula	i-FIT	i-FIT Norm	Fit Conf %	C	H	N	O	S
634.2088	634.2088	0.0	0.0	11.5	C31 H44 N3 O S5	644.6	9.776	0.01	31	44	3	1	5
	634.2088	0.0	0.0	-1.5	C16 H48 N11 O S7	646.5	11.715	0.00	16	48	11	1	7
	634.2087	0.1	0.2	19.5	C5 H12 N39 O	644.2	9.428	0.01	5	12	39	1	
	634.2089	-0.1	-0.2	-1.5	H32 N27 O8 S3	645.2	10.375	0.00		32	27	8	3
	634.2087	0.1	0.2	8.5	C7 H24 N25 O11	642.8	8.043	0.03	7	24	25	11	
	634.2089	-0.1	-0.2	0.5	C17 H40 N5 O18 S	638.2	3.399	3.34	17	40	5	18	1
	634.2089	-0.1	-0.2	11.5	C15 H28 N19 O8 S	638.4	3.619	2.68	15	28	19	8	1
	634.2086	0.2	0.3	8.5	C23 H40 N9 O4 S4	643.6	8.778	0.02	23	40	9	4	4

Figure S72. HR-ESIMS of compound **9h.**

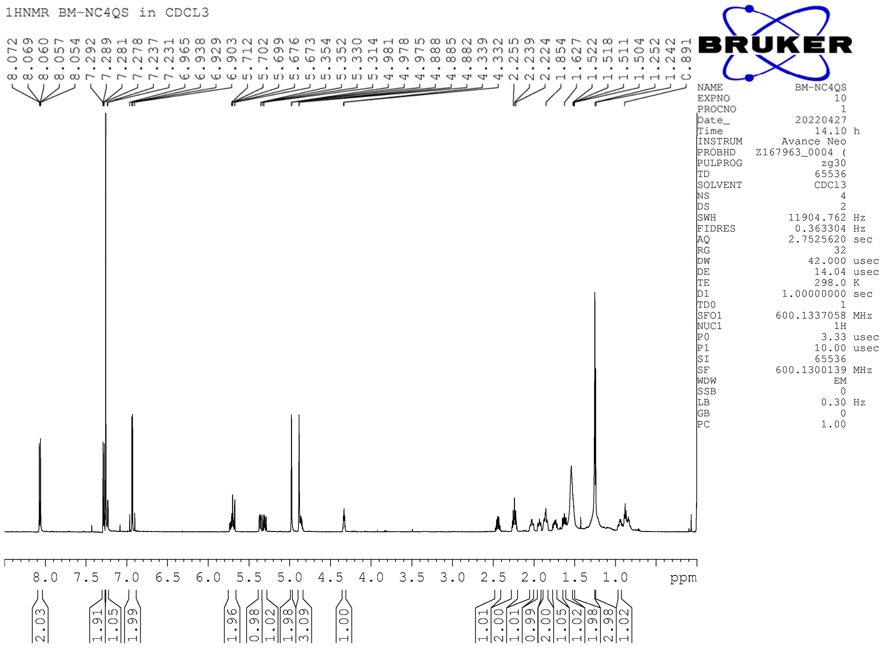

Figure S73. ^{1}H NMR of compound **9i.**

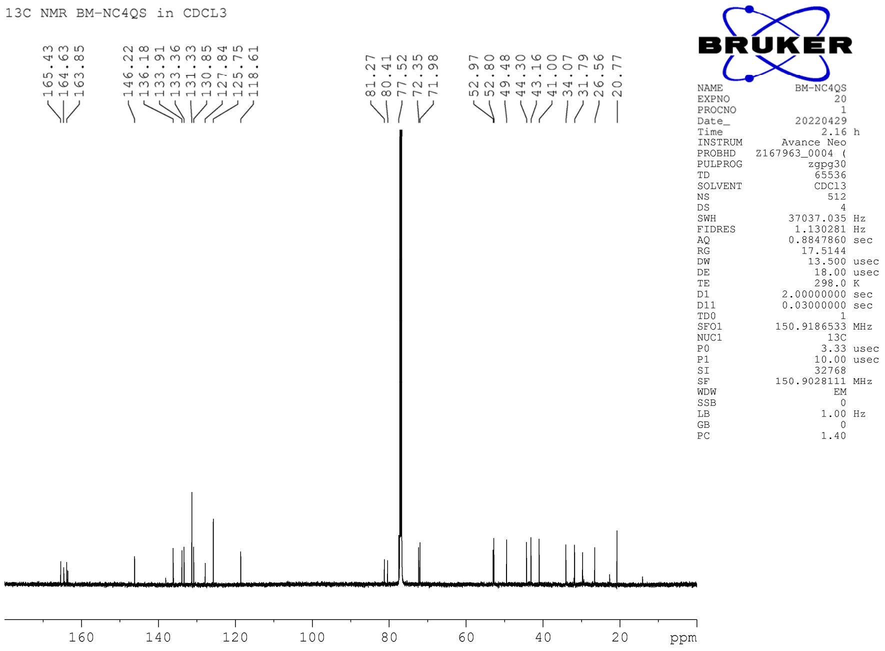

Figure S74. ^{13}C NMR of compound **9i.**

Single Mass Analysis
Tolerance = 5.0 mDa / DBE: min = -1.5, max = 50.0
Element prediction: Off
Number of isotope peaks used for i-FIT = 3
Monoisotopic Mass, Even Electron Ions
22948 formula(e) evaluated with 154 results within limits (up to 50 closest results for each mass)
Elements Used:

Mass	Calc. Mass	mDa	PPM	DBE	Formula	i-FIT	i-FIT Norm	Fit Conf %	C	H	N	O	S
630.1790	630.1790	0.0	0.0	5.5	C H24 N31 O4 S3	663.3	9.100	0.01	1	24	31	4	3
	630.1791	-0.1	-0.2	8.5	C9 H28 N25 O S4	662.6	8.373	0.02	9	28	25	1	4
	630.1789	0.1	0.2	18.5	C16 H20 N23 O4 S	657.2	2.963	5.17	16	20	23	4	1
	630.1791	-0.1	-0.2	21.5	C24 H24 N17 O S2	660.2	5.986	0.25	24	24	17	1	2
	630.1791	-0.1	-0.2	10.5	C26 H36 N3 O11 S2	661.4	7.162	0.08	26	36	3	11	2
	630.1789	0.1	0.2	7.5	C18 H32 N9 O14 S	659.8	5.558	0.39	18	32	9	14	1
	630.1791	-0.1	-0.2	34.5	C39 H20 N9 O	663.6	9.353	0.01	39	20	9	1	
	630.1788	0.2	0.3	4.5	C10 H28 N15 O17	664.4	10.207	0.00	10	28	15	17	

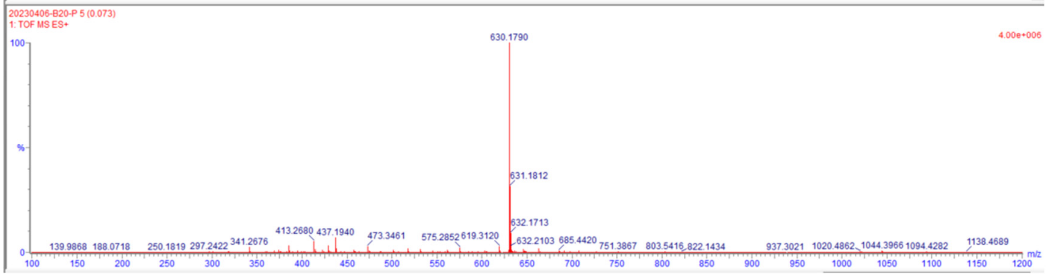

Figure S75. HR-ESIMS of compound **9i.**

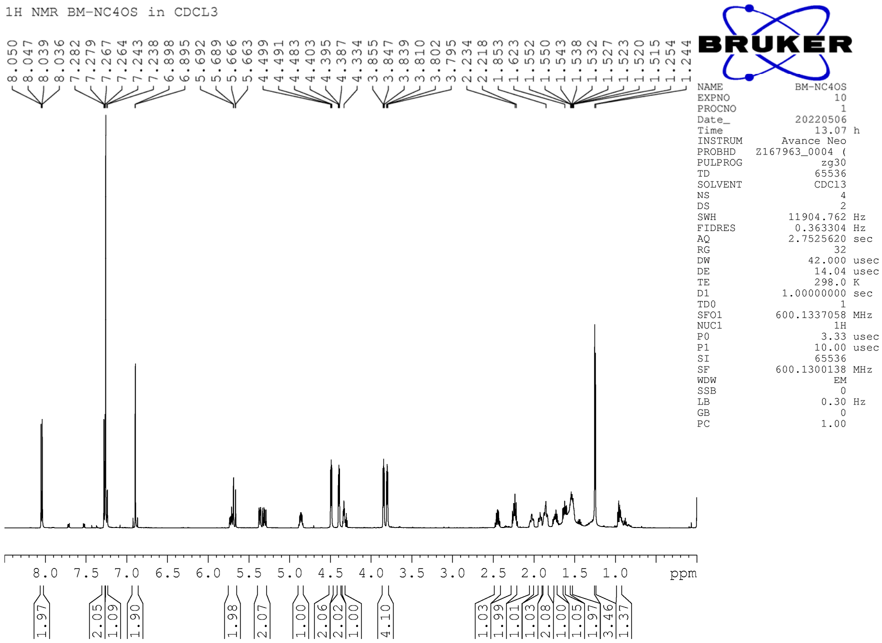

Figure S76. ^{1}H NMR of compound **9j.**

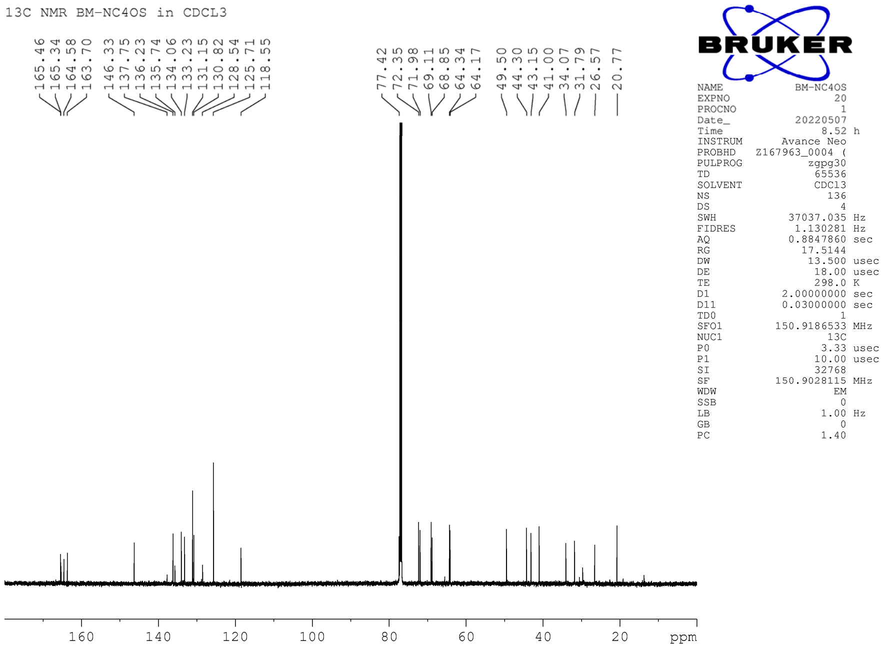

Figure S77. ^{13}C NMR of compound **9j.**

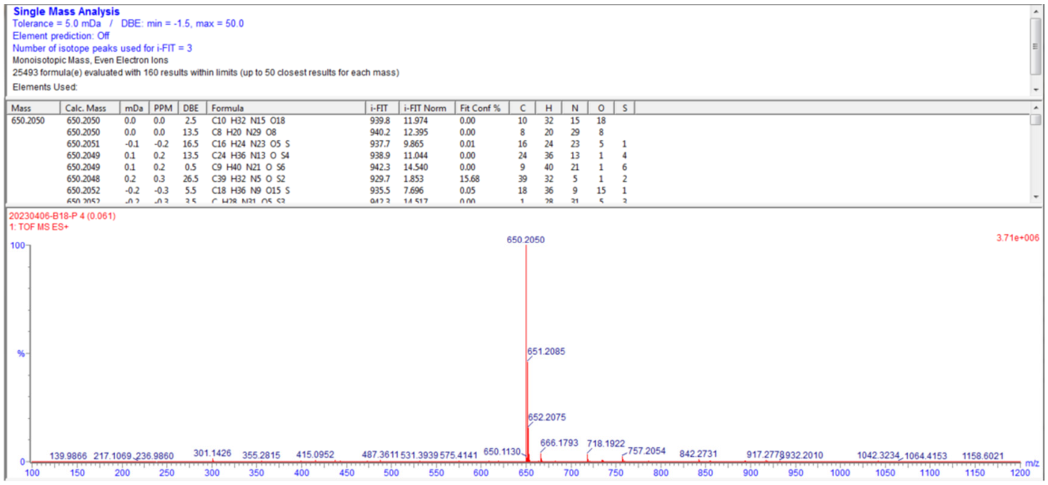

Mass	Calc. Mass	mDa	PPM	DBE	Formula	i-FIT	i-FIT Norm	Fit Conf %	C	H	N	O	S
650.2050	650.2050	0.0	0.0	2.5	C10 H32 N15 O18	939.8	11.974	0.00	10	32	15	18	
	650.2050	0.0	0.0	13.5	C8 H20 N29 O8	940.2	12.395	0.00	8	20	29	8	
	650.2051	-0.1	-0.2	16.5	C16 H24 N23 O5 S	937.7	9.865	0.01	16	24	23	5	1
	650.2049	0.1	0.2	13.5	C24 H36 N13 O S4	938.9	11.044	0.00	24	36	13	1	4
	650.2049	0.1	0.2	0.5	C9 H40 N21 O S6	942.3	14.540	0.00	9	40	21	1	6
	650.2048	0.2	0.3	26.5	C39 H32 N5 O S2	929.7	1.853	15.68	39	32	5	1	2
	650.2052	-0.2	-0.3	5.5	C18 H36 N9 O15 S	935.5	7.696	0.05	18	36	9	15	1

Figure S78. HR-ESIMS of compound **9j.**

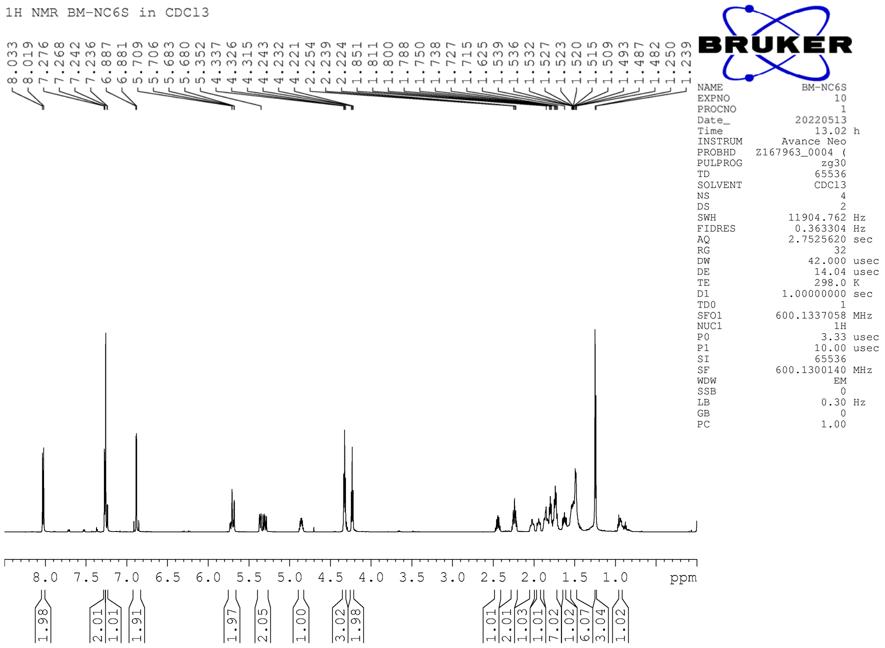

Figure S79. ^{1}H NMR of compound **9k.**

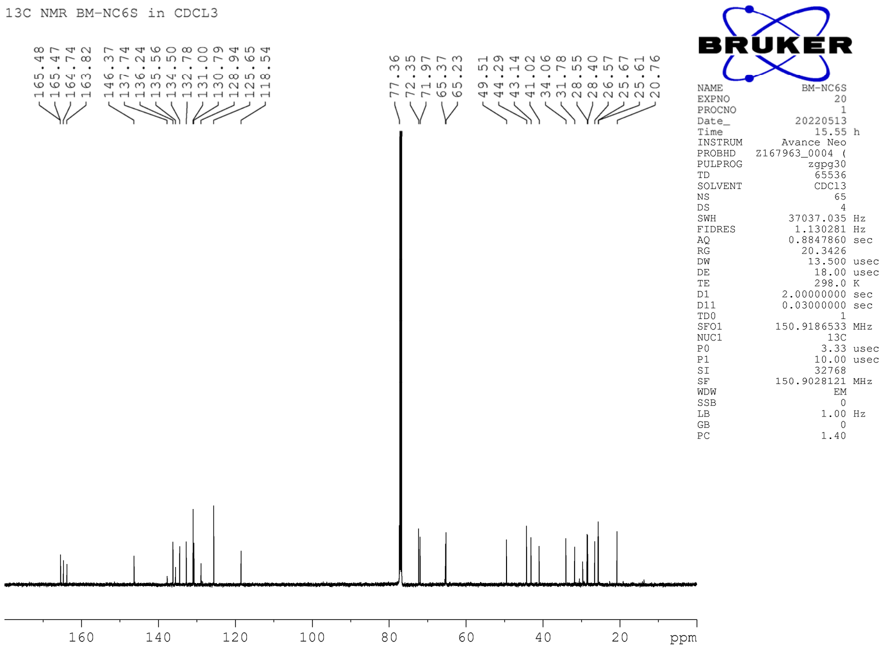

Figure S80. ^{13}C NMR of compound **9k.**

Single Mass Analysis
Tolerance = 5.0 mDa / DBE: min = -1.5, max = 50.0
Element prediction: Off
Number of isotope peaks used for i-FIT = 3
Monoisotopic Mass, Even Electron Ions
27160 formula(e) evaluated with 151 results within limits (up to 50 closest results for each mass)
Elements Used:

Mass	Calc. Mass	mDa	PPM	DBE	Formula	i-FIT	i-FIT Norm	Fit Conf %	C	H	N	O	S
662.2433	662.2433	0.0	0.0	3.5	C22 H48 N9 O4 S5	744.1	9.938	0.00	22	48	9	4	5
	662.2432	0.1	0.2	16.5	C37 H44 N O4 S3	741.7	7.607	0.05	37	44	1	4	3
	662.2434	-0.1	-0.2	3.5	C6 H32 N25 O11 S	741.3	7.141	0.08	6	32	25	11	1
	662.2434	-0.1	-0.2	14.5	C4 H20 N39 O S	741.9	7.718	0.04	4	20	39	1	1
	662.2431	0.2	0.3	0.5	C14 H44 N15 O7 S4	743.4	9.243	0.01	14	44	15	7	4
	662.2431	0.2	0.3	13.5	C29 H40 N7 O7 S2	739.3	5.197	0.55	29	40	7	7	2
	662.2435	-0.2	-0.3	6.5	C30 H52 N3 O S6	744.8	10.659	0.00	30	52	3	1	6
	662.2435	-0.2	-0.3	19.5	C29 H32 N11 O8	738.1	3.951	1.92	29	32	11	8	

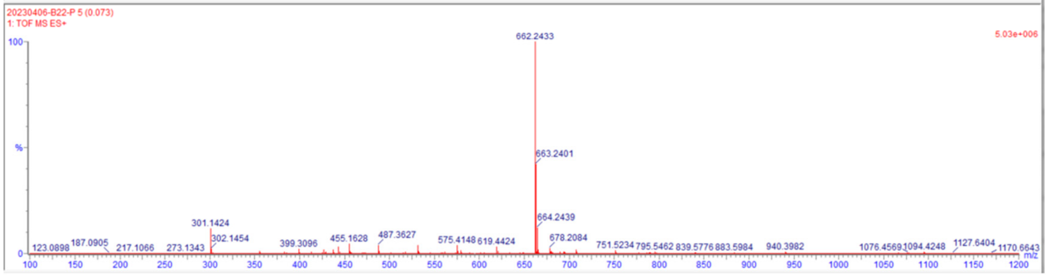

Figure S81. HR-ESIMS of compound **9k.**
